# Supplementary material for: Use of a Rubric to Improve the Quality of Internal Medicine Resident Event Reporting
Source: MedEdPORTAL. 2021 Oct 11;17:11189. doi: 10.15766/mep_2374-8265.11189 (PMC8502786; doi:10.15766/mep_2374-8265.11189)
Supplement: Supplementary file 1 — Pretraining Survey.docxPosttraining Survey.docxResident Training Module.pptxInstructor Guide.docxResident Training Module Script.docxI-SAFEST Scoring Sheet.docx [file mep_2374-8265.11189-s001.zip › C. Resident Training Module.pptx]

## Slide 1
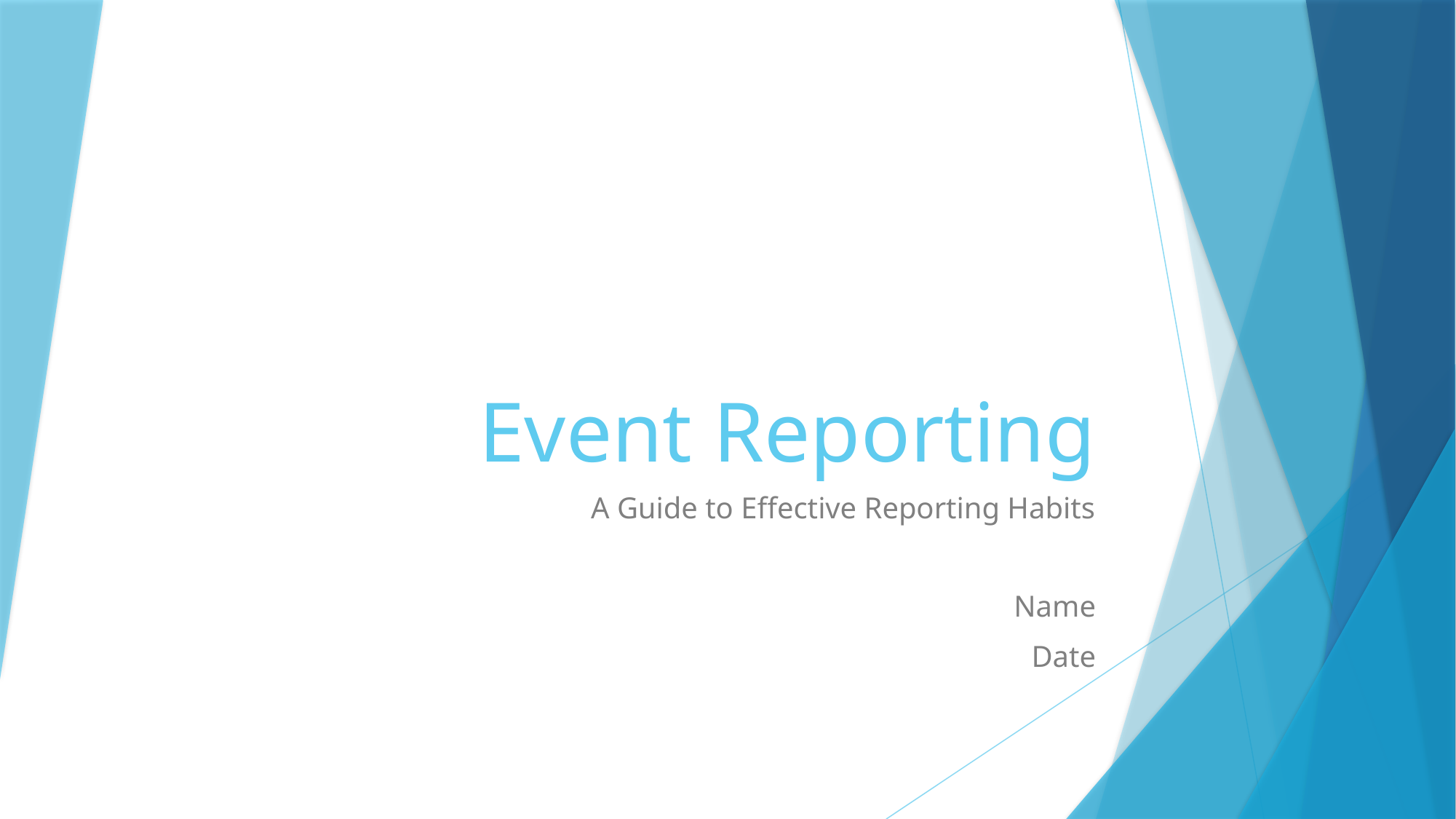

# Event Reporting
A Guide to Effective Reporting Habits
Name
Date

## Slide 2
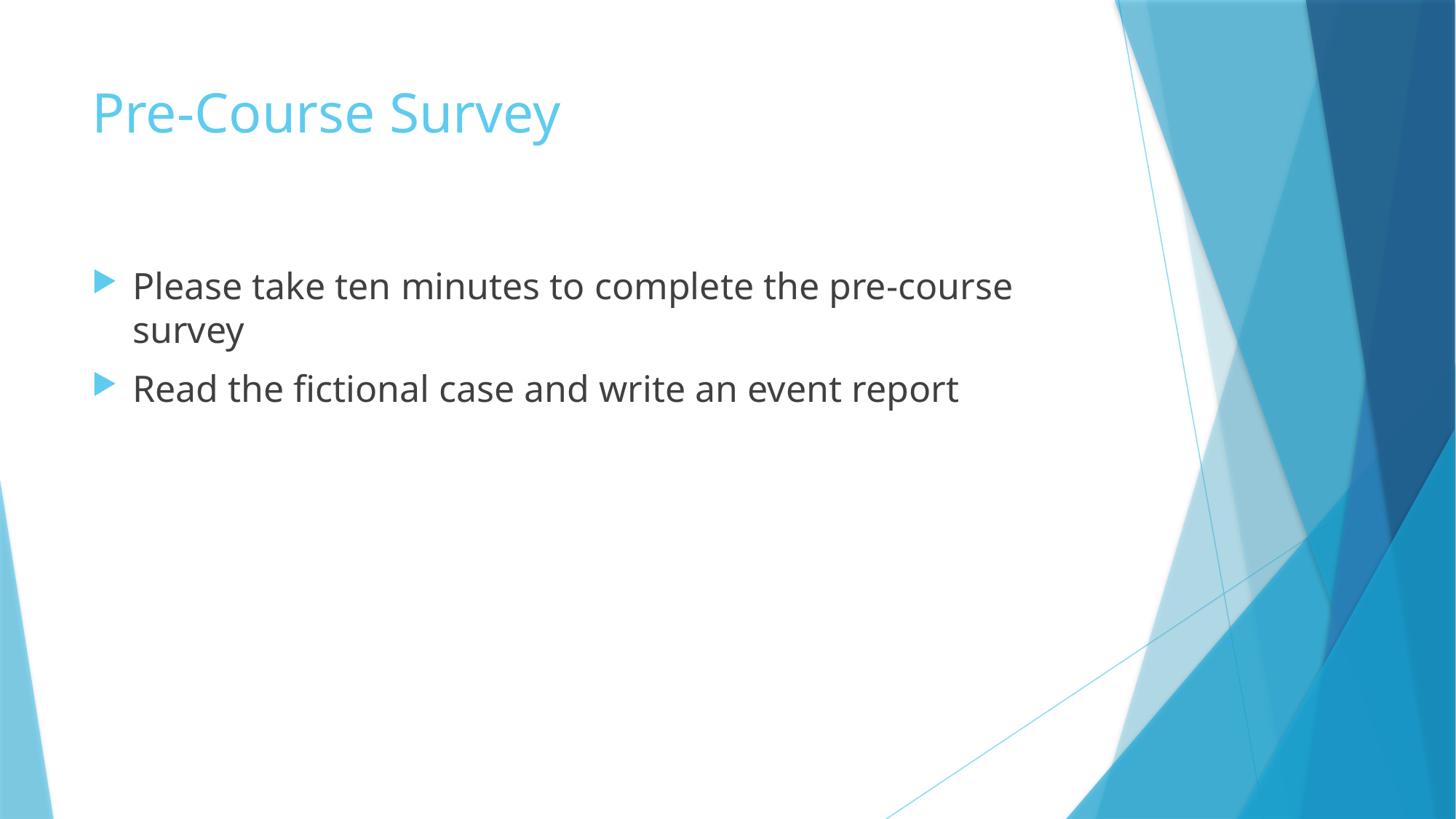

# Pre-Course Survey
Please take ten minutes to complete the pre-course survey
Read the fictional case and write an event report

## Slide 3
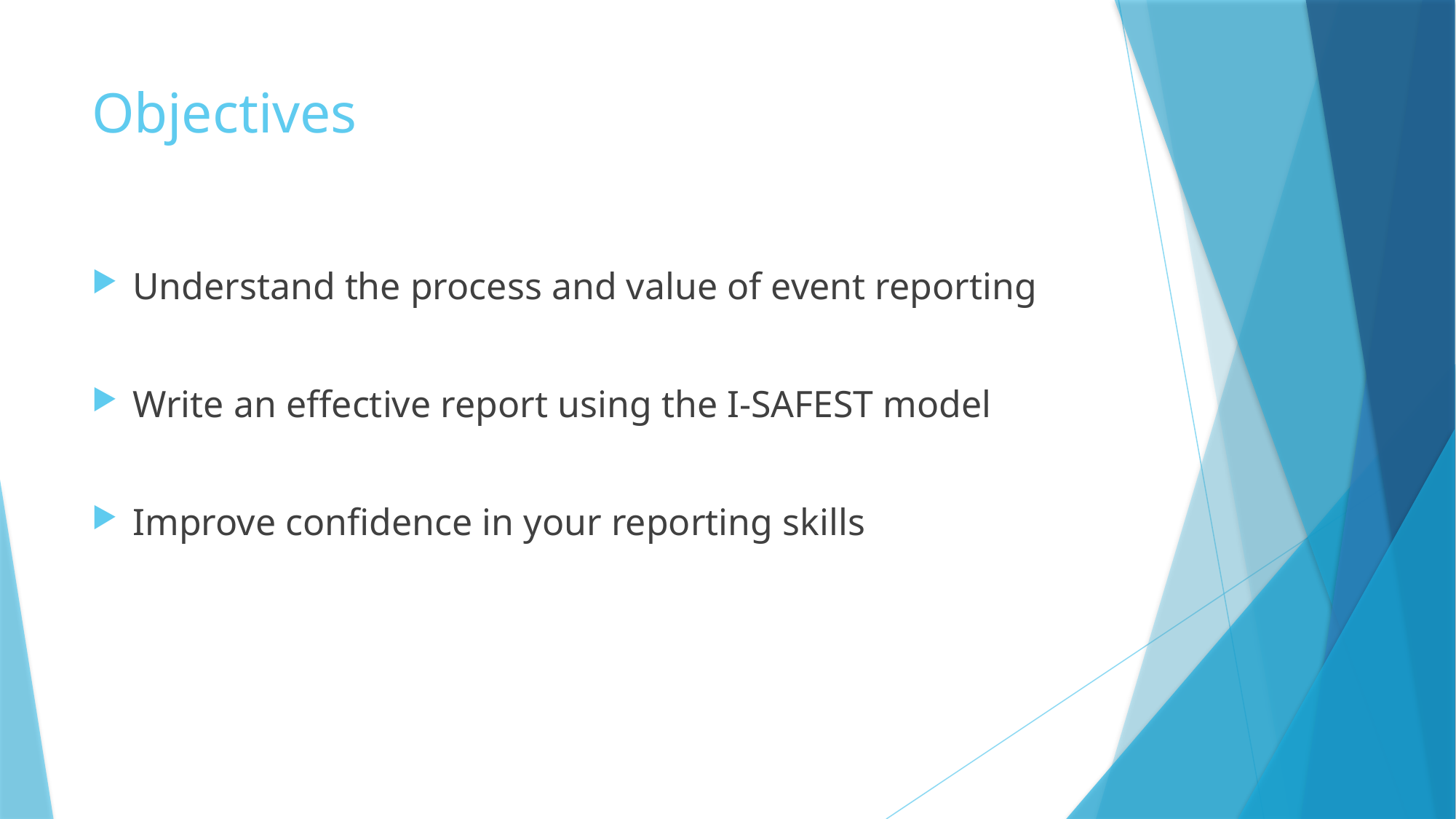

# Objectives
Understand the process and value of event reporting
Write an effective report using the I-SAFEST model
Improve confidence in your reporting skills

## Slide 4
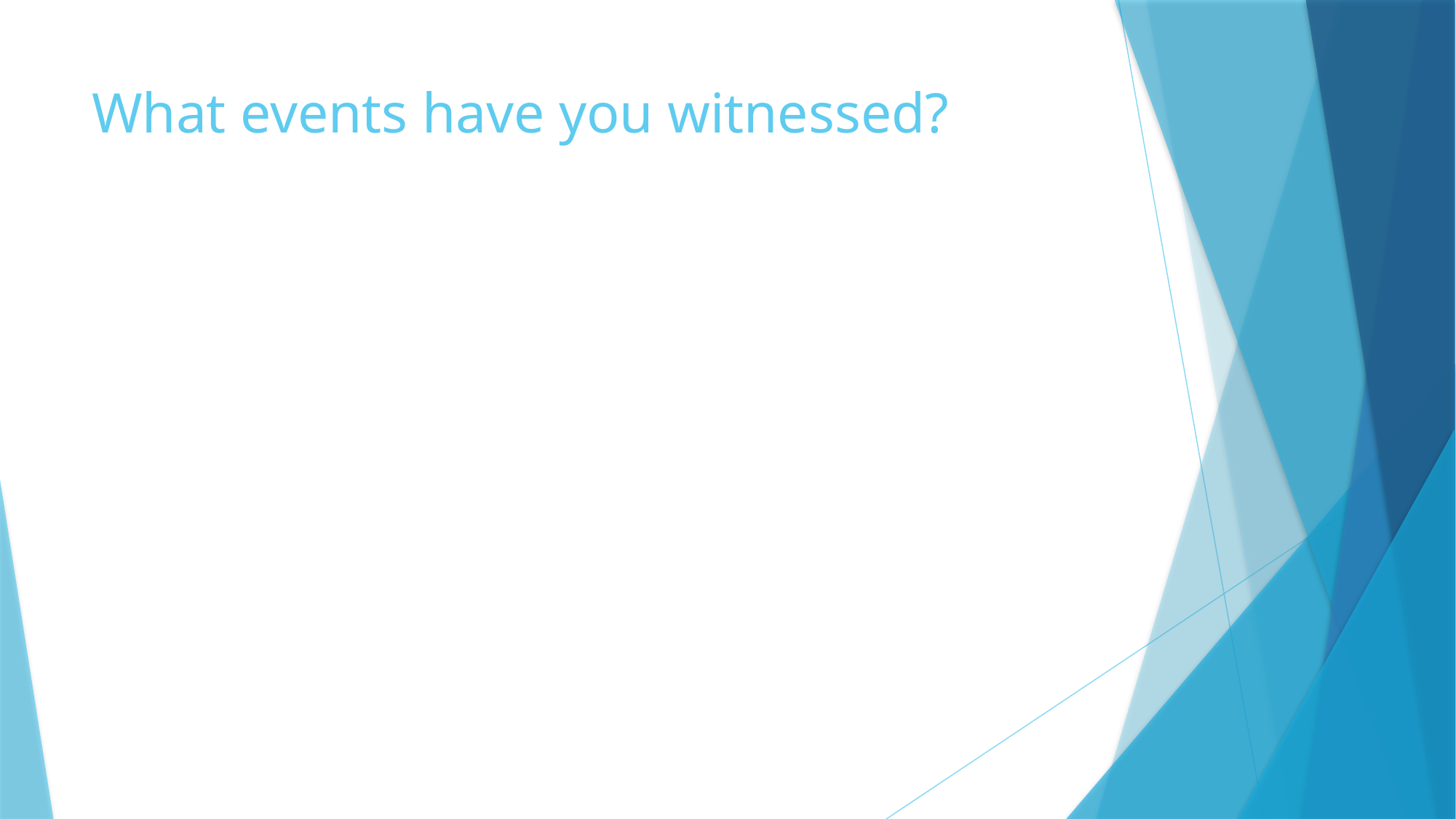

# What events have you witnessed?

## Slide 5
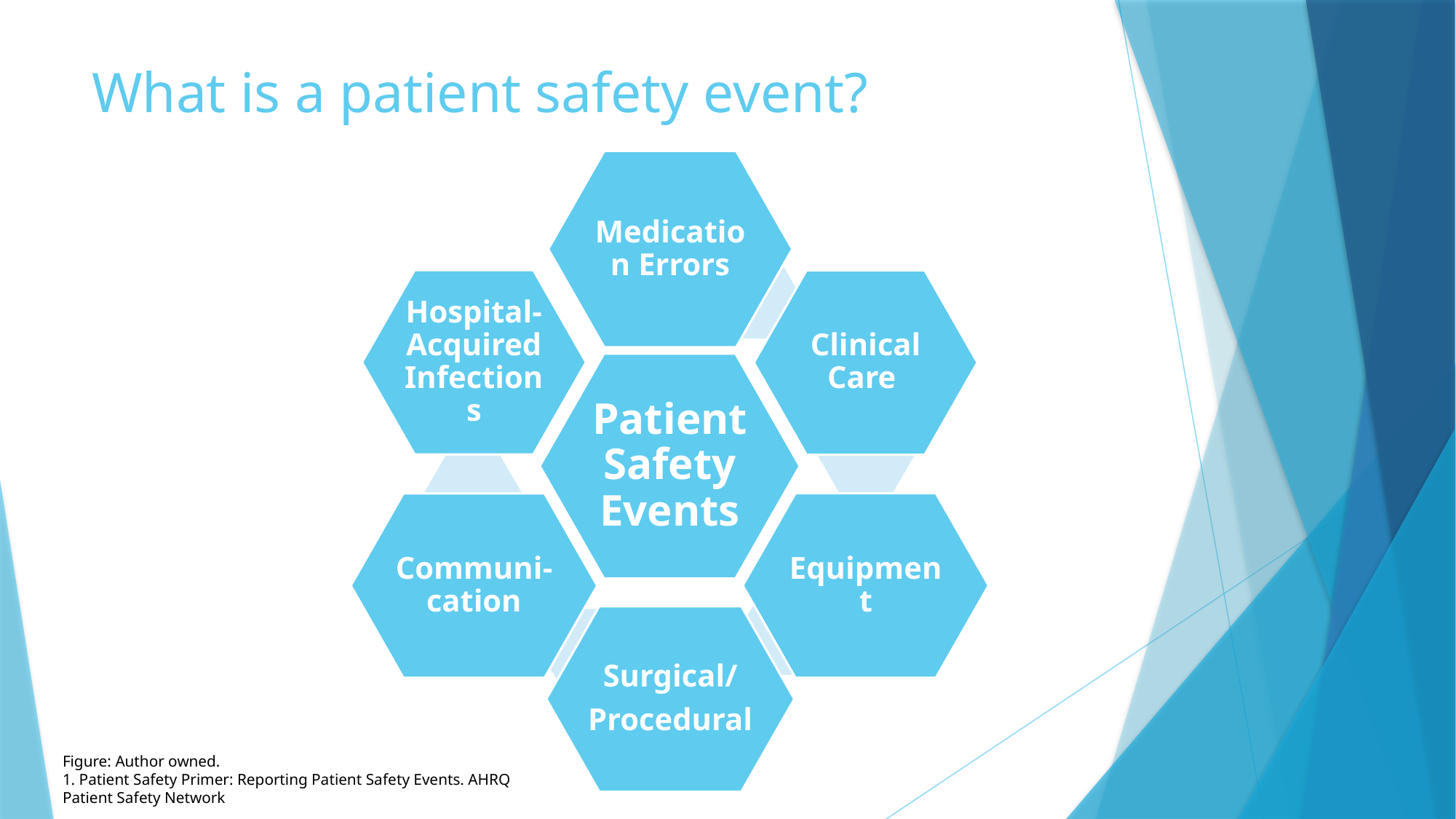

# What is a patient safety event?
Figure: Author owned.
1. Patient Safety Primer: Reporting Patient Safety Events. AHRQ Patient Safety Network

## Slide 6
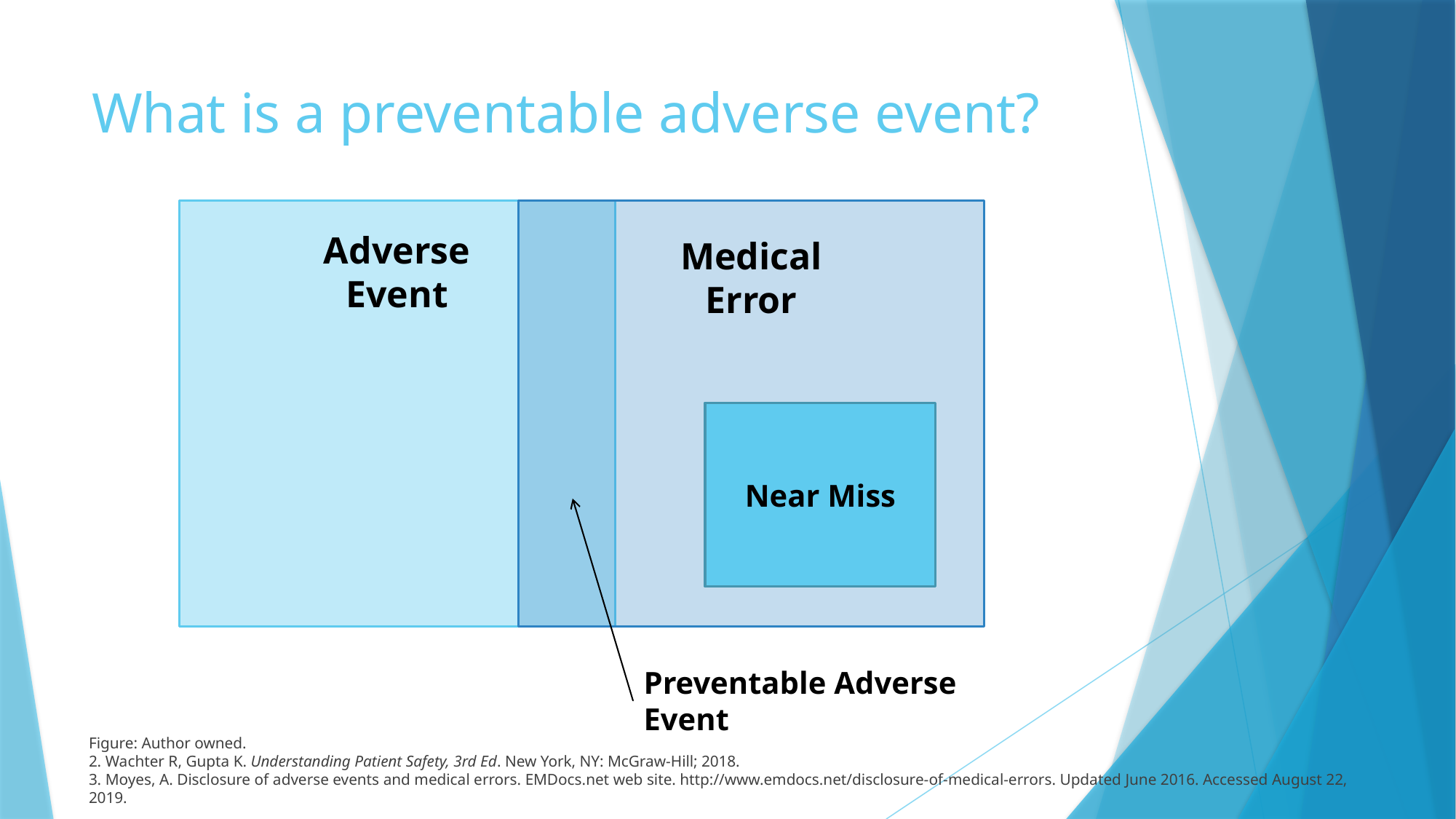

# What is a preventable adverse event?
Adverse
Event
Medical
Error
Near Miss
Preventable Adverse Event
Figure: Author owned.
2. Wachter R, Gupta K. Understanding Patient Safety, 3rd Ed. New York, NY: McGraw-Hill; 2018.
3. Moyes, A. Disclosure of adverse events and medical errors. EMDocs.net web site. http://www.emdocs.net/disclosure-of-medical-errors. Updated June 2016. Accessed August 22, 2019.

## Slide 7
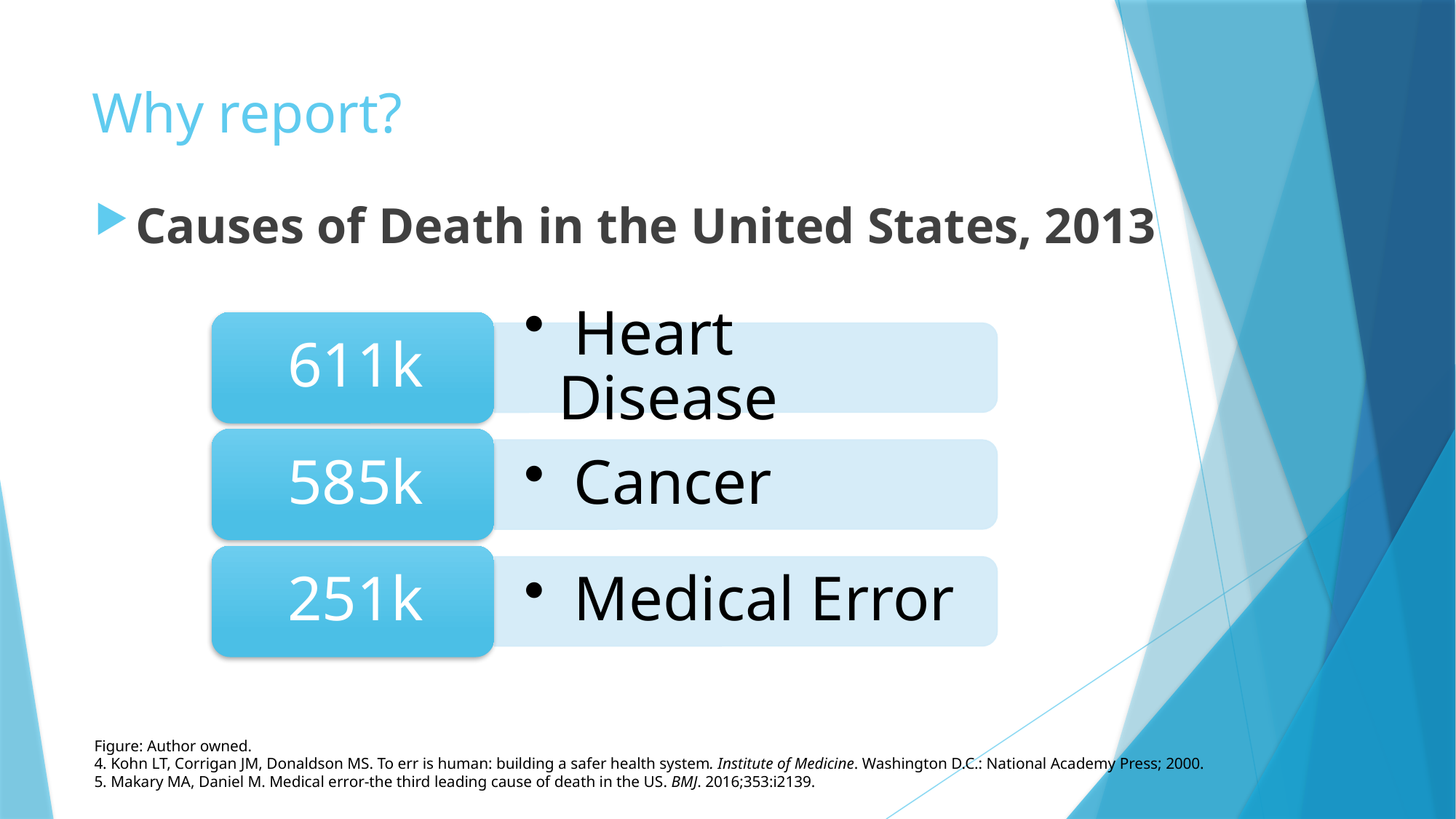

# Why report?
Causes of Death in the United States, 2013
Figure: Author owned.
4. Kohn LT, Corrigan JM, Donaldson MS. To err is human: building a safer health system. Institute of Medicine. Washington D.C.: National Academy Press; 2000.
5. Makary MA, Daniel M. Medical error-the third leading cause of death in the US. BMJ. 2016;353:i2139.

## Slide 8
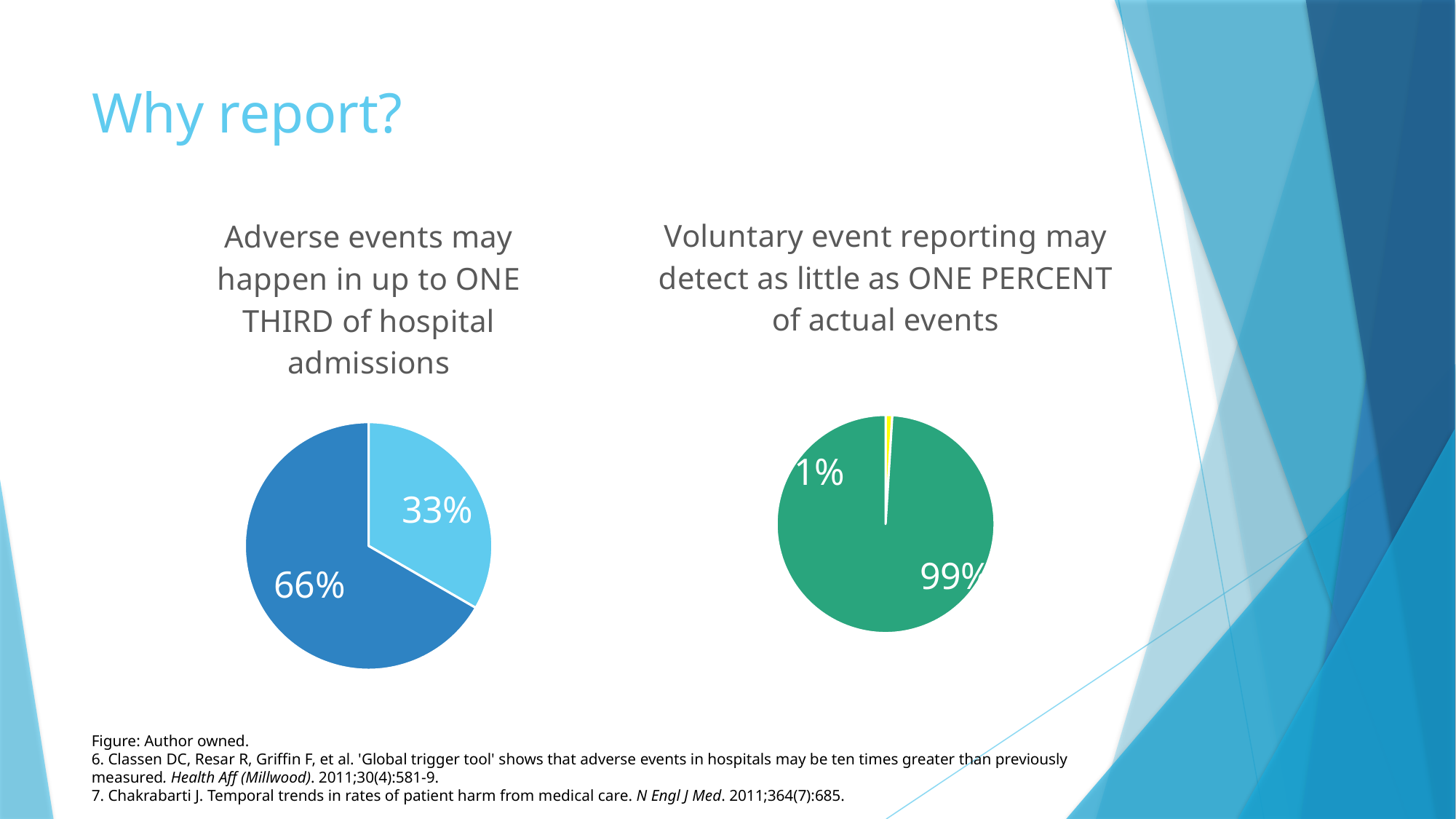

# Why report?
### Chart: Voluntary event reporting may detect as little as ONE PERCENT of actual events
| Category | Voluntary event reporting may detect as little as one percent of actual events |
|---|---|
| Reported event | 0.01 |
| Unreported event | 0.99 |
### Chart: Adverse events may happen in up to ONE THIRD of hospital admissions
| Category | Estimated adverse events per hospital admission |
|---|---|
| Adverse event | 0.33 |
| No event | 0.66 |Figure: Author owned.
6. Classen DC, Resar R, Griffin F, et al. 'Global trigger tool' shows that adverse events in hospitals may be ten times greater than previously measured. Health Aff (Millwood). 2011;30(4):581-9.
7. Chakrabarti J. Temporal trends in rates of patient harm from medical care. N Engl J Med. 2011;364(7):685.

## Slide 9
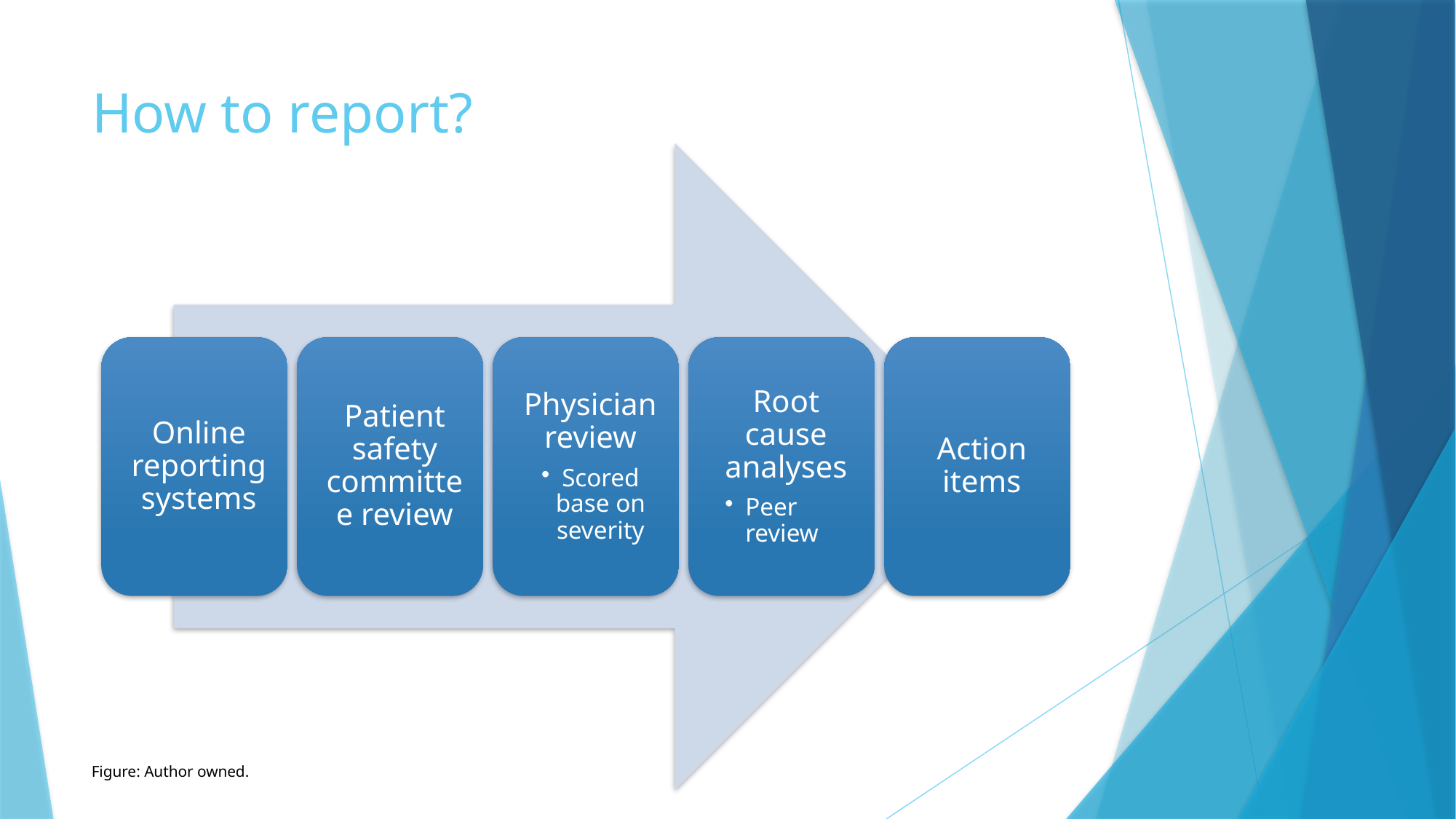

# How to report?
Figure: Author owned.

## Slide 10
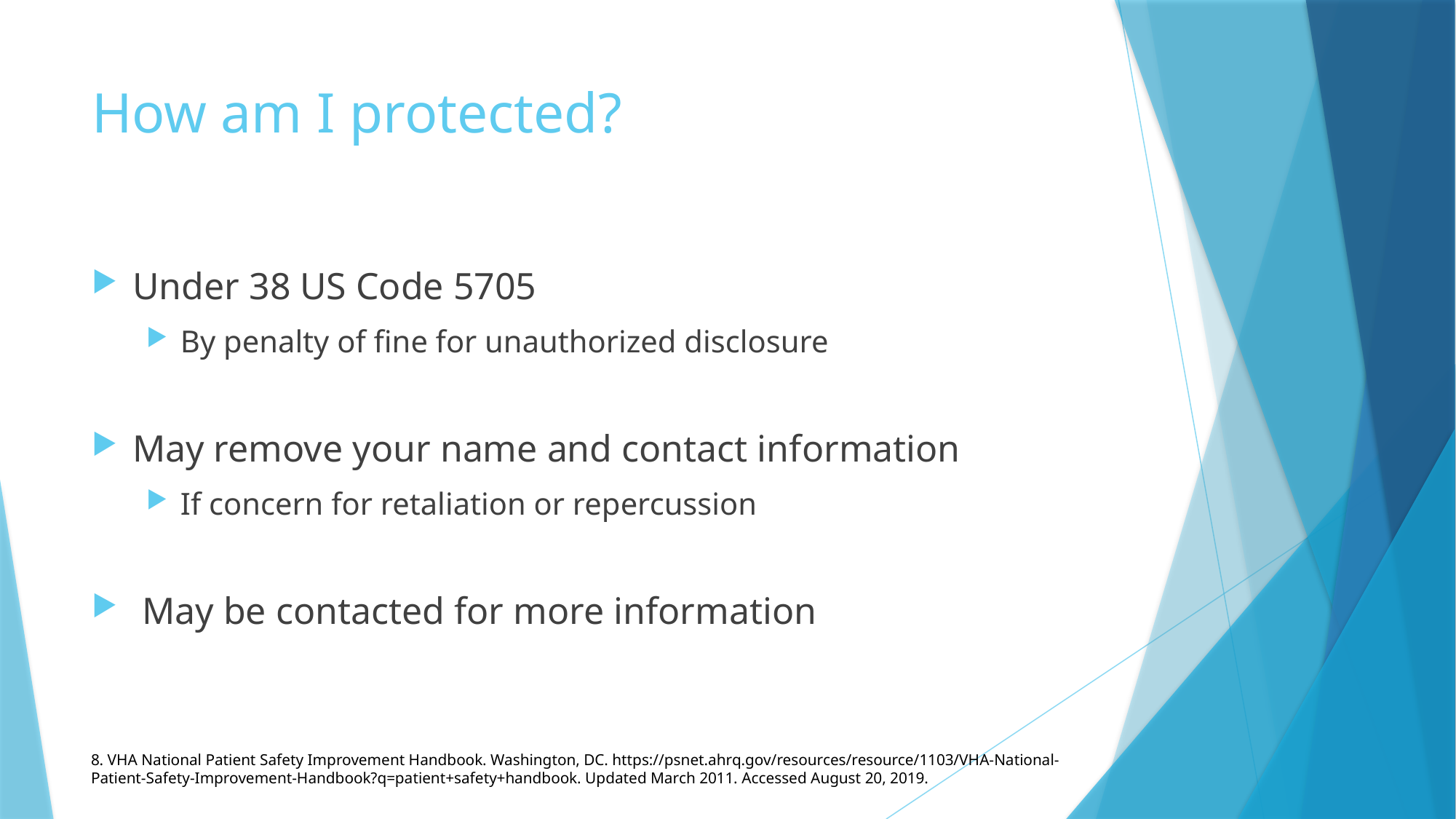

# How am I protected?
Under 38 US Code 5705
By penalty of fine for unauthorized disclosure
May remove your name and contact information
If concern for retaliation or repercussion
 May be contacted for more information
8. VHA National Patient Safety Improvement Handbook. Washington, DC. https://psnet.ahrq.gov/resources/resource/1103/VHA-National-Patient-Safety-Improvement-Handbook?q=patient+safety+handbook. Updated March 2011. Accessed August 20, 2019.

## Slide 11
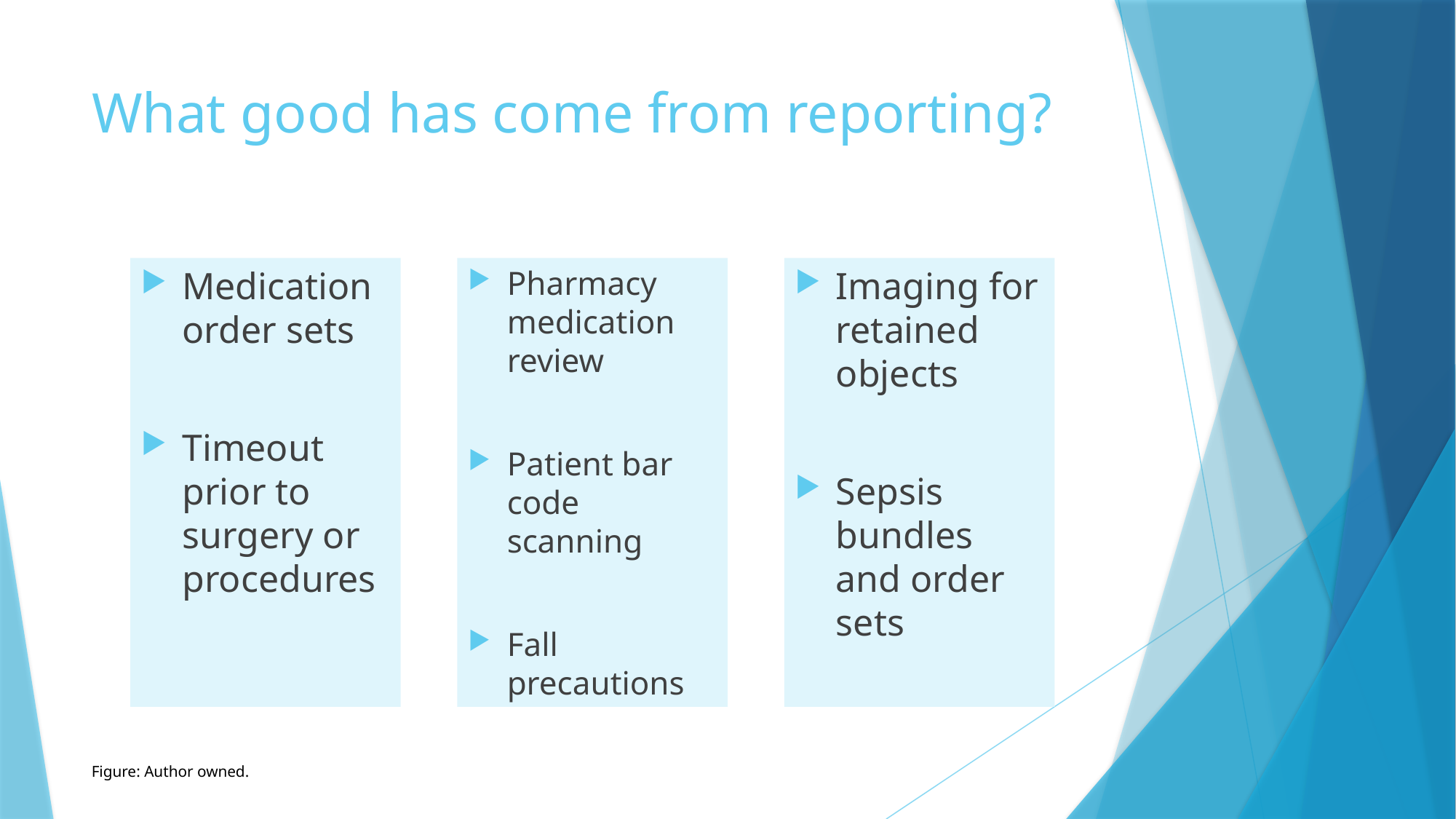

# What good has come from reporting?
Medication order sets
Timeout prior to surgery or procedures
Pharmacy medication review
Patient bar code scanning
Fall precautions
Imaging for retained objects
Sepsis bundles and order sets
Figure: Author owned.

## Slide 12
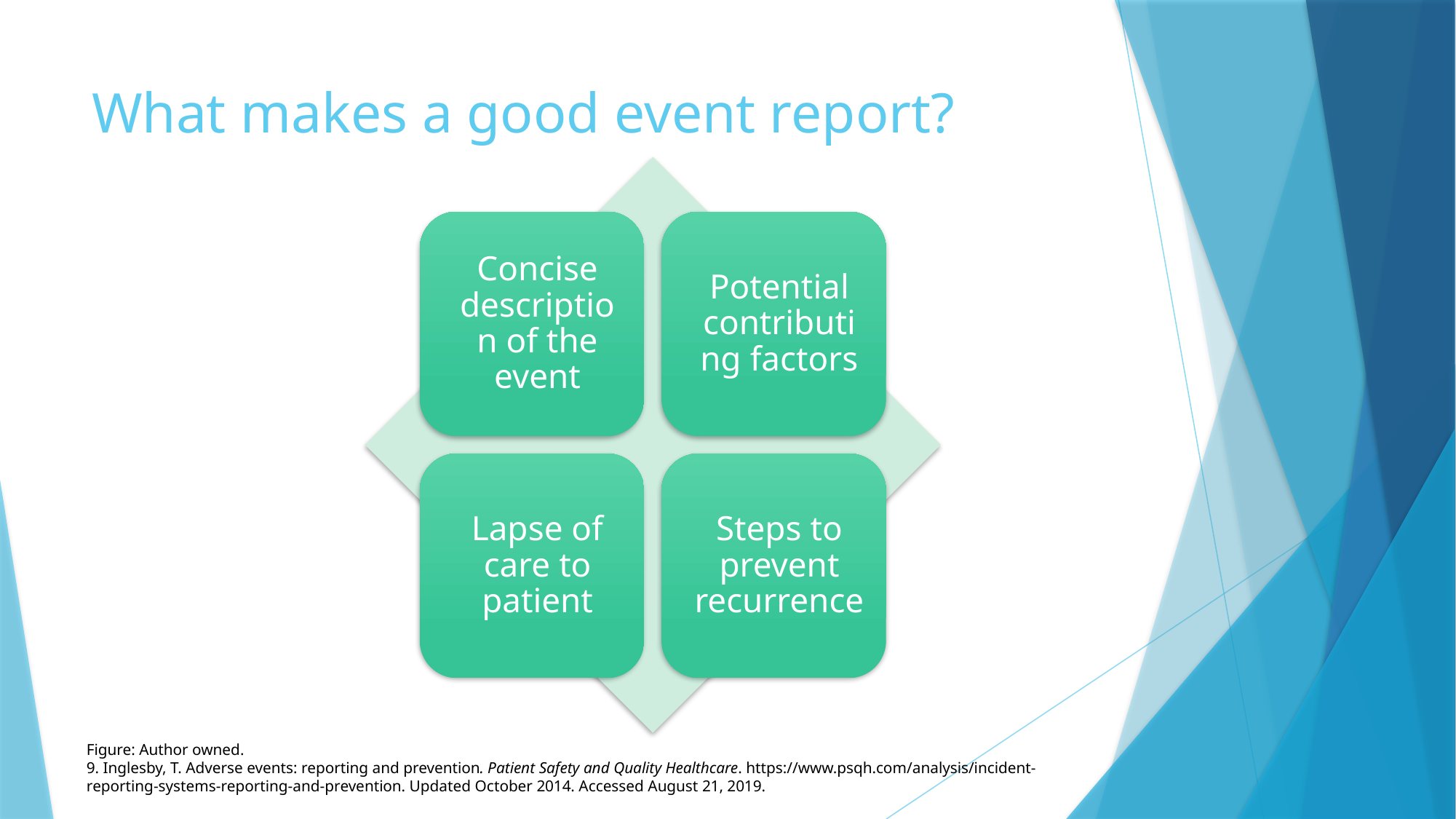

# What makes a good event report?
Figure: Author owned.
9. Inglesby, T. Adverse events: reporting and prevention. Patient Safety and Quality Healthcare. https://www.psqh.com/analysis/incident-
reporting-systems-reporting-and-prevention. Updated October 2014. Accessed August 21, 2019.

## Slide 13
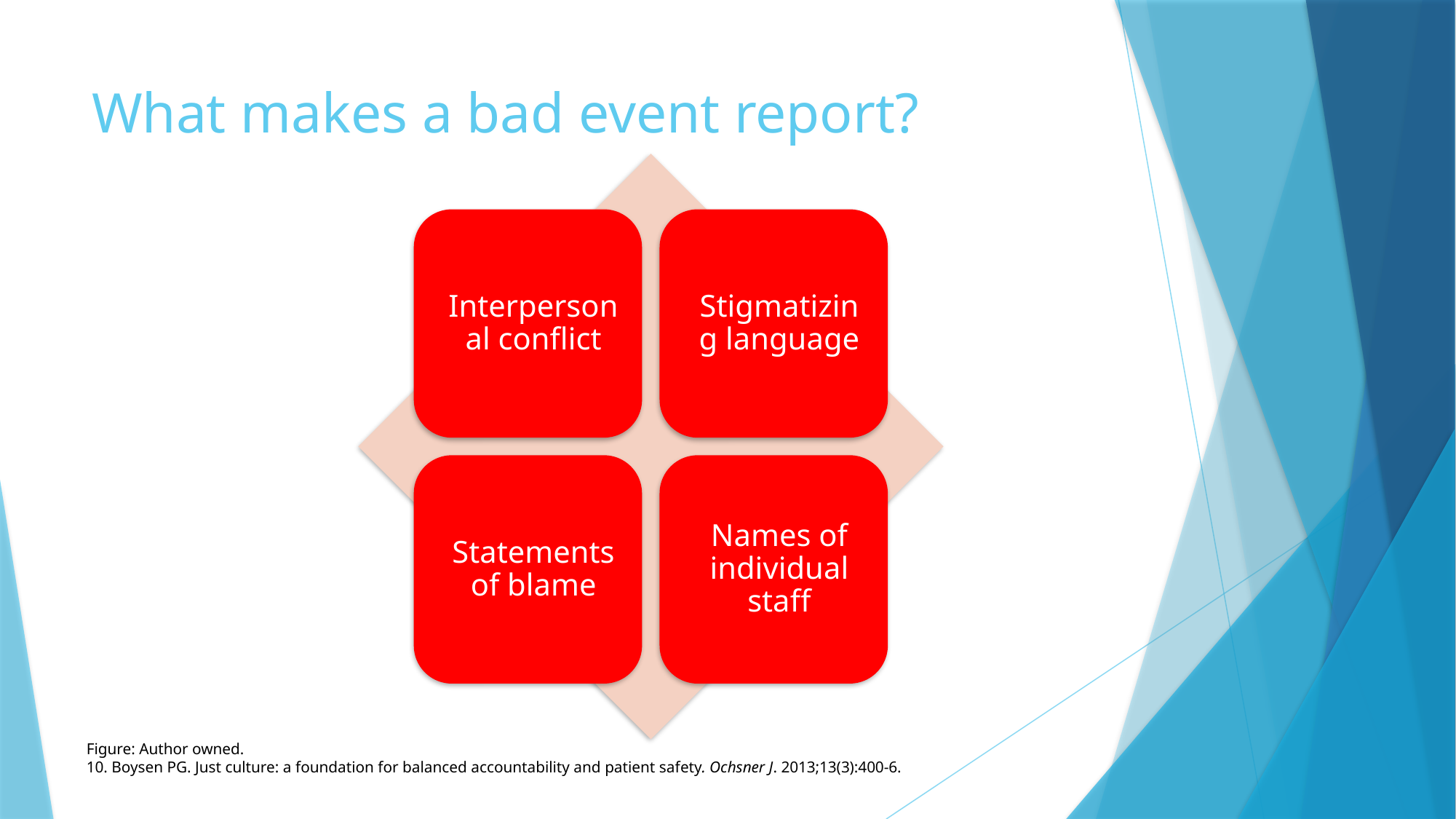

# What makes a bad event report?
Figure: Author owned.
10. Boysen PG. Just culture: a foundation for balanced accountability and patient safety. Ochsner J. 2013;13(3):400-6.

## Slide 14
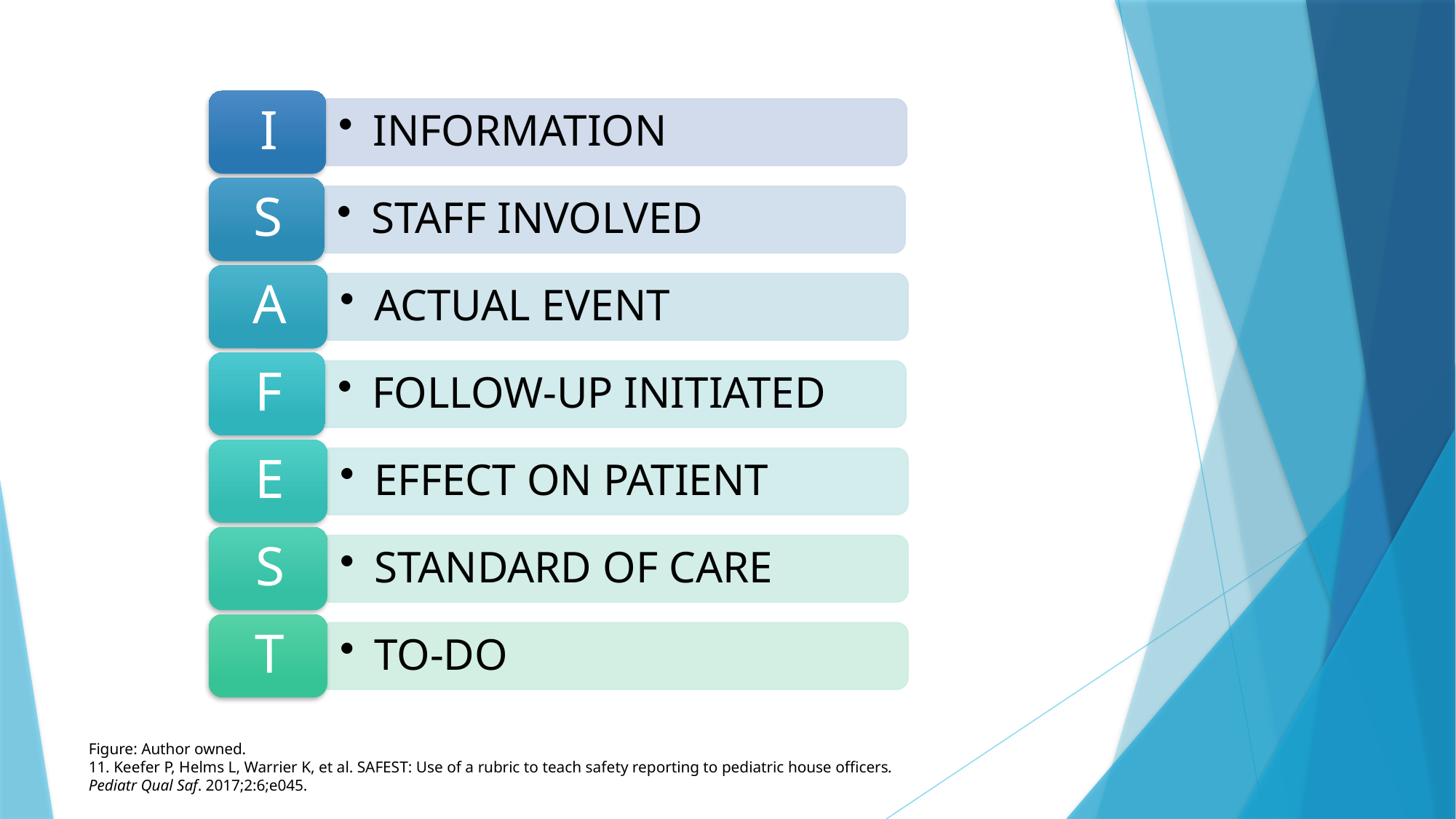

Figure: Author owned.
11. Keefer P, Helms L, Warrier K, et al. SAFEST: Use of a rubric to teach safety reporting to pediatric house officers.
Pediatr Qual Saf. 2017;2:6;e045.

## Slide 15
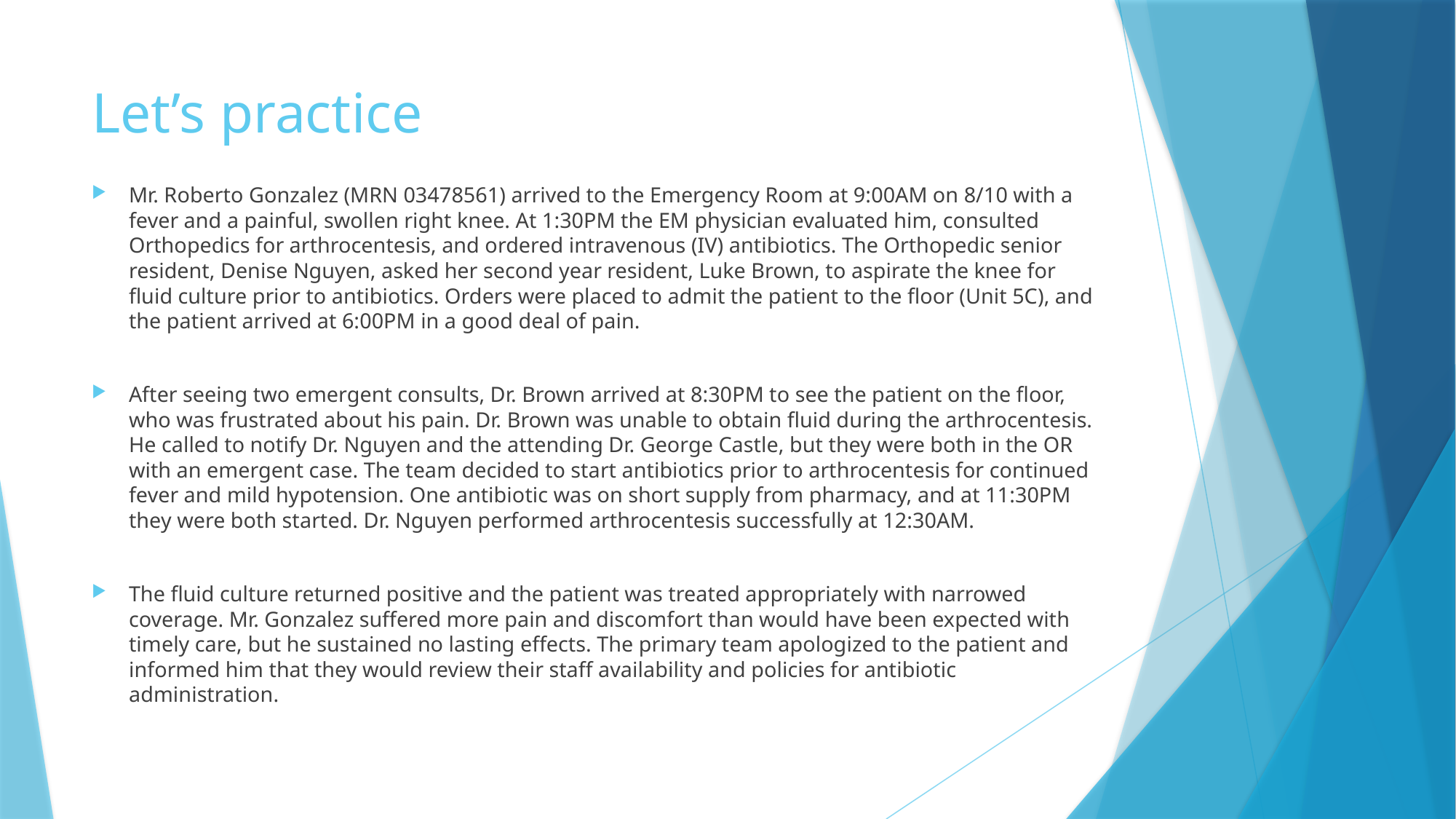

# Let’s practice
Mr. Roberto Gonzalez (MRN 03478561) arrived to the Emergency Room at 9:00AM on 8/10 with a fever and a painful, swollen right knee. At 1:30PM the EM physician evaluated him, consulted Orthopedics for arthrocentesis, and ordered intravenous (IV) antibiotics. The Orthopedic senior resident, Denise Nguyen, asked her second year resident, Luke Brown, to aspirate the knee for fluid culture prior to antibiotics. Orders were placed to admit the patient to the floor (Unit 5C), and the patient arrived at 6:00PM in a good deal of pain.
After seeing two emergent consults, Dr. Brown arrived at 8:30PM to see the patient on the floor, who was frustrated about his pain. Dr. Brown was unable to obtain fluid during the arthrocentesis. He called to notify Dr. Nguyen and the attending Dr. George Castle, but they were both in the OR with an emergent case. The team decided to start antibiotics prior to arthrocentesis for continued fever and mild hypotension. One antibiotic was on short supply from pharmacy, and at 11:30PM they were both started. Dr. Nguyen performed arthrocentesis successfully at 12:30AM.
The fluid culture returned positive and the patient was treated appropriately with narrowed coverage. Mr. Gonzalez suffered more pain and discomfort than would have been expected with timely care, but he sustained no lasting effects. The primary team apologized to the patient and informed him that they would review their staff availability and policies for antibiotic administration.

## Slide 16
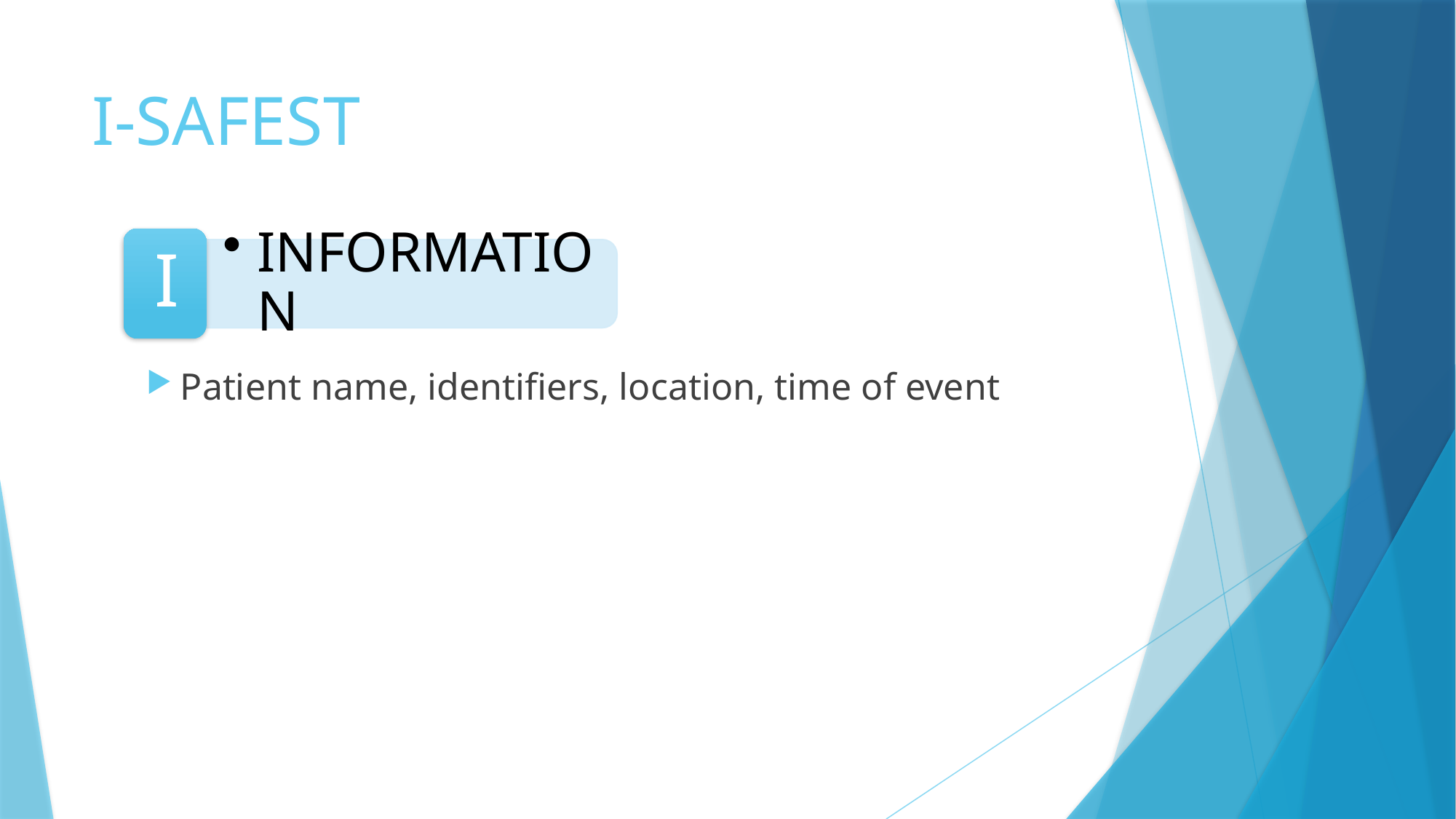

# I-SAFEST
Patient name, identifiers, location, time of event

## Slide 17
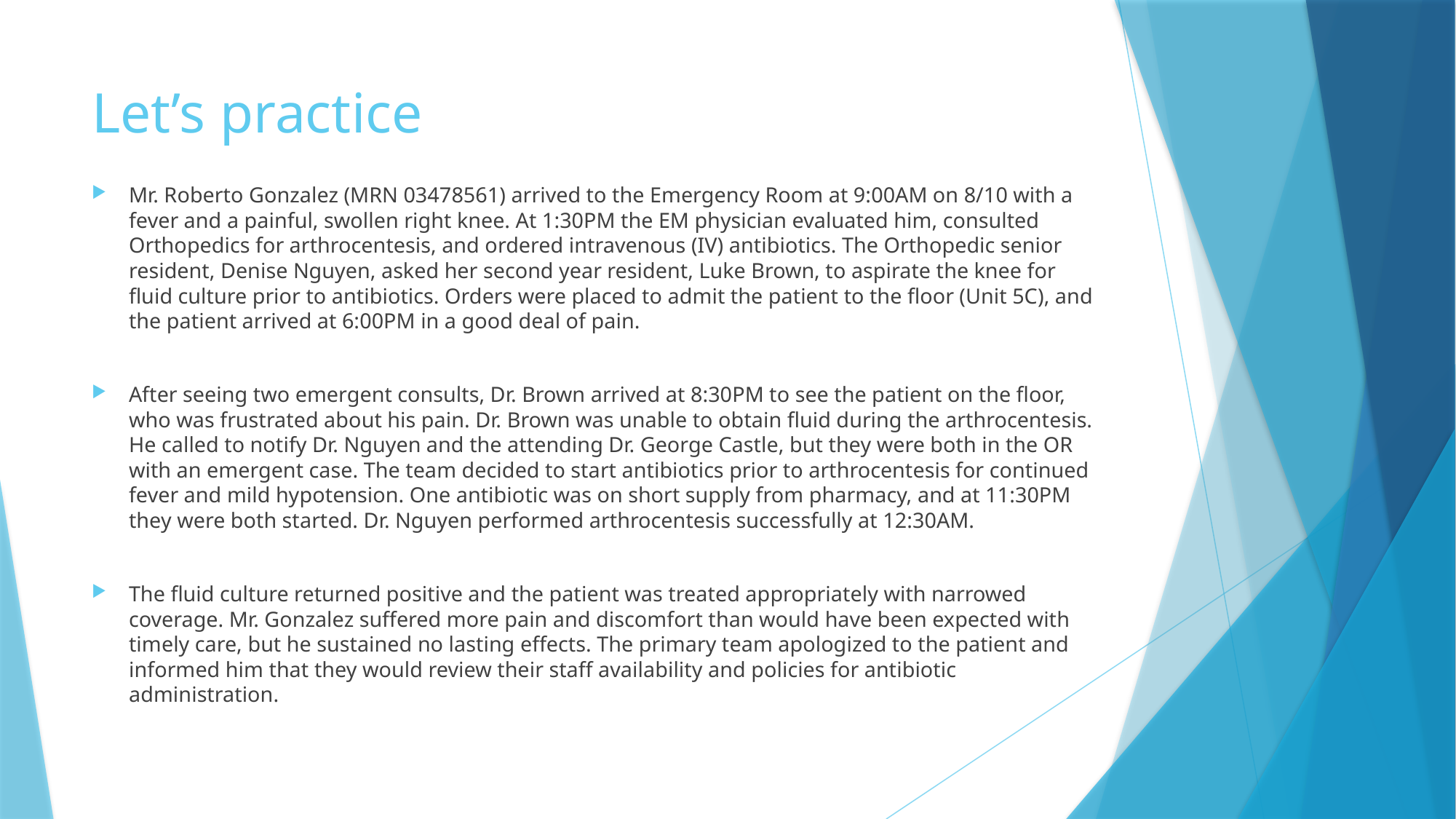

# Let’s practice
Mr. Roberto Gonzalez (MRN 03478561) arrived to the Emergency Room at 9:00AM on 8/10 with a fever and a painful, swollen right knee. At 1:30PM the EM physician evaluated him, consulted Orthopedics for arthrocentesis, and ordered intravenous (IV) antibiotics. The Orthopedic senior resident, Denise Nguyen, asked her second year resident, Luke Brown, to aspirate the knee for fluid culture prior to antibiotics. Orders were placed to admit the patient to the floor (Unit 5C), and the patient arrived at 6:00PM in a good deal of pain.
After seeing two emergent consults, Dr. Brown arrived at 8:30PM to see the patient on the floor, who was frustrated about his pain. Dr. Brown was unable to obtain fluid during the arthrocentesis. He called to notify Dr. Nguyen and the attending Dr. George Castle, but they were both in the OR with an emergent case. The team decided to start antibiotics prior to arthrocentesis for continued fever and mild hypotension. One antibiotic was on short supply from pharmacy, and at 11:30PM they were both started. Dr. Nguyen performed arthrocentesis successfully at 12:30AM.
The fluid culture returned positive and the patient was treated appropriately with narrowed coverage. Mr. Gonzalez suffered more pain and discomfort than would have been expected with timely care, but he sustained no lasting effects. The primary team apologized to the patient and informed him that they would review their staff availability and policies for antibiotic administration.

## Slide 18
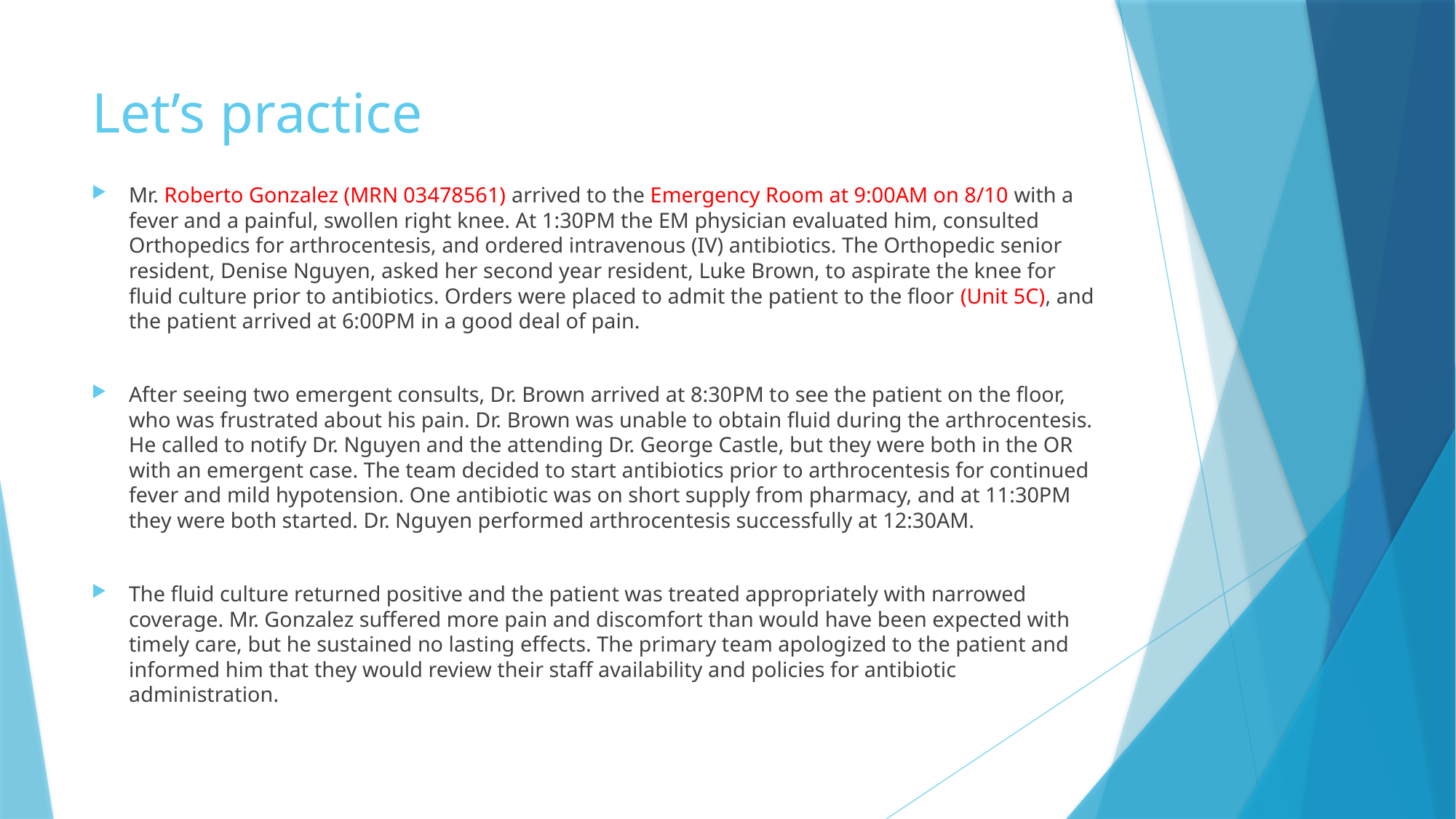

# Let’s practice
Mr. Roberto Gonzalez (MRN 03478561) arrived to the Emergency Room at 9:00AM on 8/10 with a fever and a painful, swollen right knee. At 1:30PM the EM physician evaluated him, consulted Orthopedics for arthrocentesis, and ordered intravenous (IV) antibiotics. The Orthopedic senior resident, Denise Nguyen, asked her second year resident, Luke Brown, to aspirate the knee for fluid culture prior to antibiotics. Orders were placed to admit the patient to the floor (Unit 5C), and the patient arrived at 6:00PM in a good deal of pain.
After seeing two emergent consults, Dr. Brown arrived at 8:30PM to see the patient on the floor, who was frustrated about his pain. Dr. Brown was unable to obtain fluid during the arthrocentesis. He called to notify Dr. Nguyen and the attending Dr. George Castle, but they were both in the OR with an emergent case. The team decided to start antibiotics prior to arthrocentesis for continued fever and mild hypotension. One antibiotic was on short supply from pharmacy, and at 11:30PM they were both started. Dr. Nguyen performed arthrocentesis successfully at 12:30AM.
The fluid culture returned positive and the patient was treated appropriately with narrowed coverage. Mr. Gonzalez suffered more pain and discomfort than would have been expected with timely care, but he sustained no lasting effects. The primary team apologized to the patient and informed him that they would review their staff availability and policies for antibiotic administration.

## Slide 19
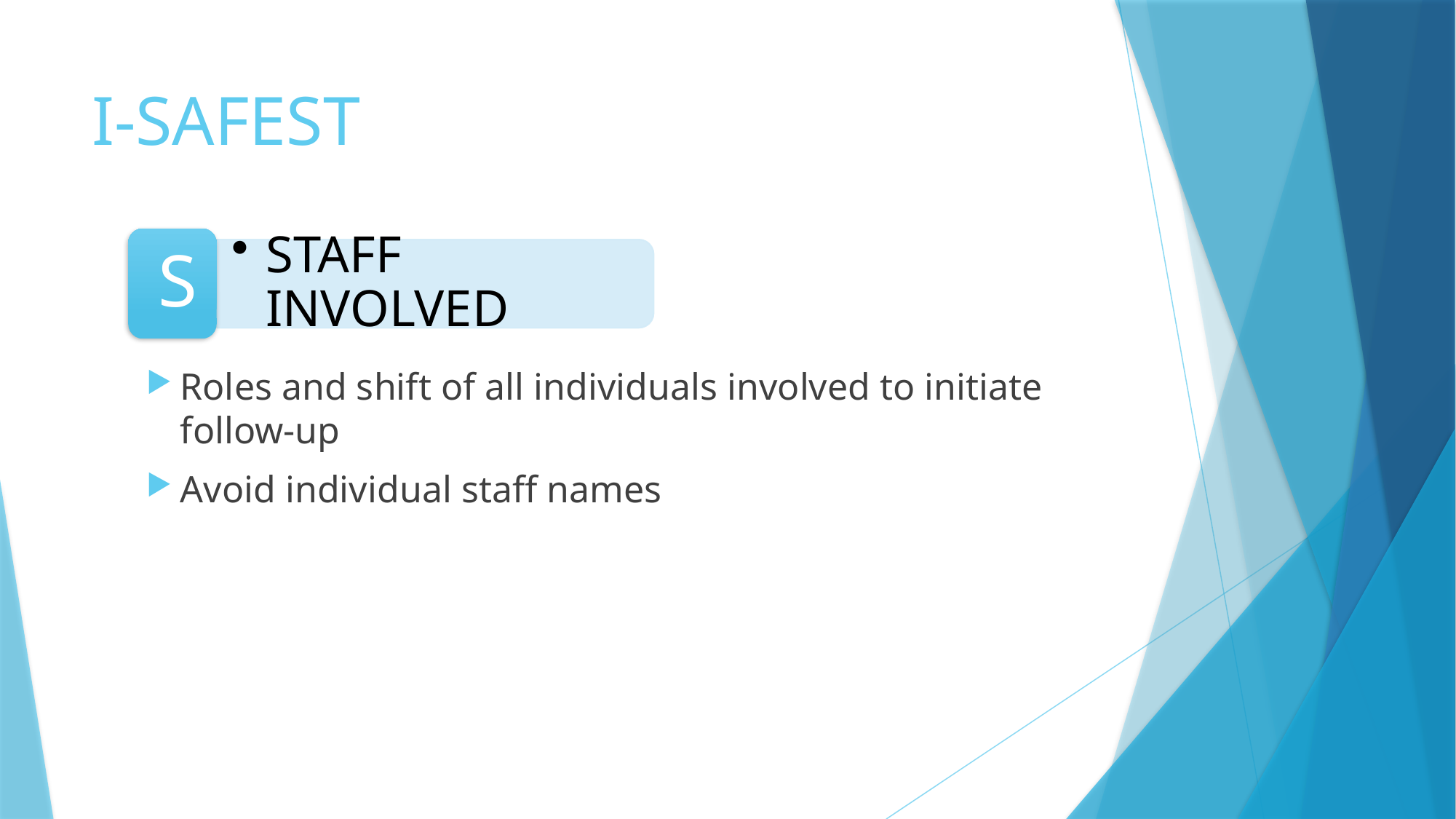

# I-SAFEST
Roles and shift of all individuals involved to initiate follow-up
Avoid individual staff names

## Slide 20
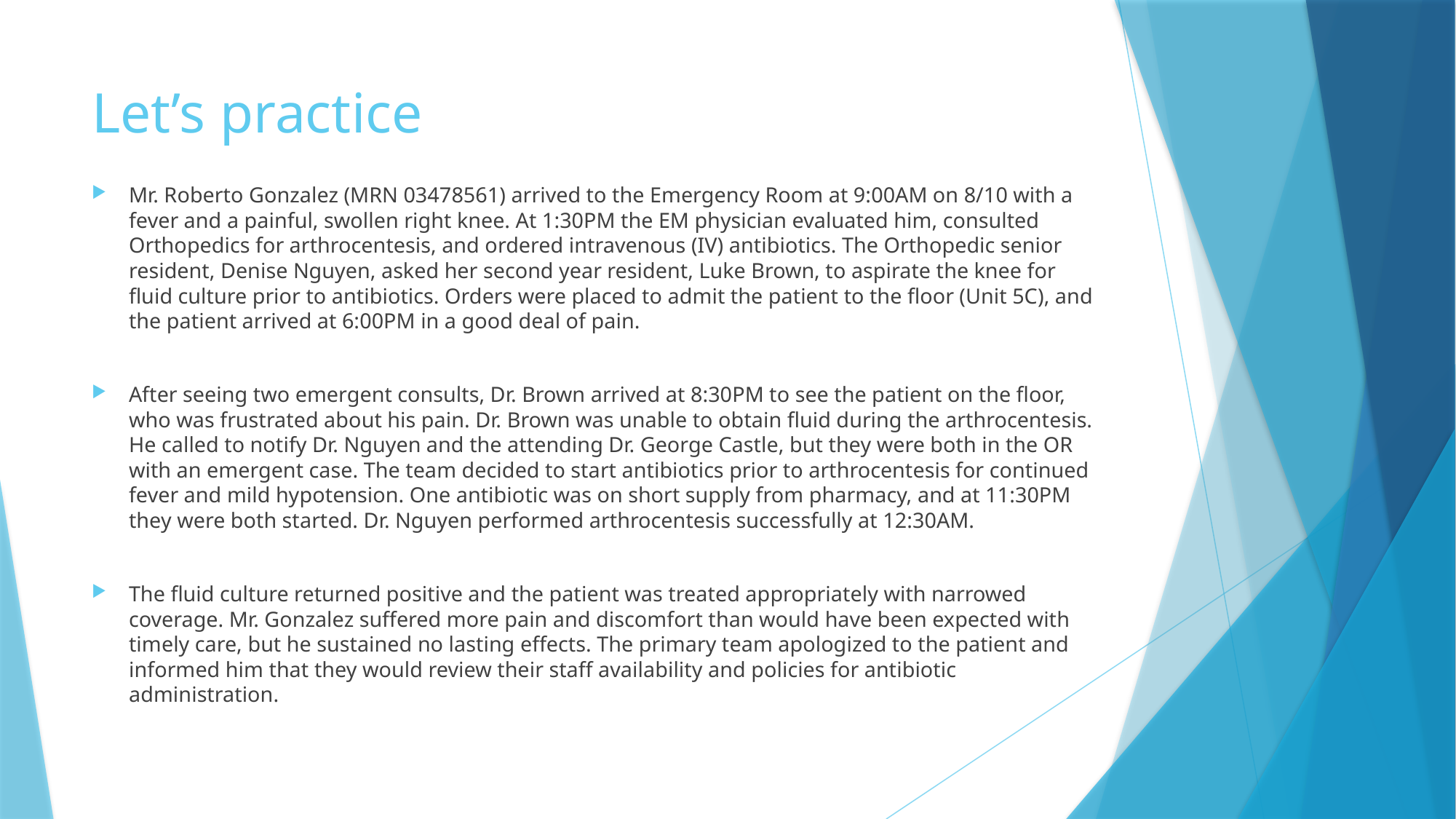

# Let’s practice
Mr. Roberto Gonzalez (MRN 03478561) arrived to the Emergency Room at 9:00AM on 8/10 with a fever and a painful, swollen right knee. At 1:30PM the EM physician evaluated him, consulted Orthopedics for arthrocentesis, and ordered intravenous (IV) antibiotics. The Orthopedic senior resident, Denise Nguyen, asked her second year resident, Luke Brown, to aspirate the knee for fluid culture prior to antibiotics. Orders were placed to admit the patient to the floor (Unit 5C), and the patient arrived at 6:00PM in a good deal of pain.
After seeing two emergent consults, Dr. Brown arrived at 8:30PM to see the patient on the floor, who was frustrated about his pain. Dr. Brown was unable to obtain fluid during the arthrocentesis. He called to notify Dr. Nguyen and the attending Dr. George Castle, but they were both in the OR with an emergent case. The team decided to start antibiotics prior to arthrocentesis for continued fever and mild hypotension. One antibiotic was on short supply from pharmacy, and at 11:30PM they were both started. Dr. Nguyen performed arthrocentesis successfully at 12:30AM.
The fluid culture returned positive and the patient was treated appropriately with narrowed coverage. Mr. Gonzalez suffered more pain and discomfort than would have been expected with timely care, but he sustained no lasting effects. The primary team apologized to the patient and informed him that they would review their staff availability and policies for antibiotic administration.

## Slide 21
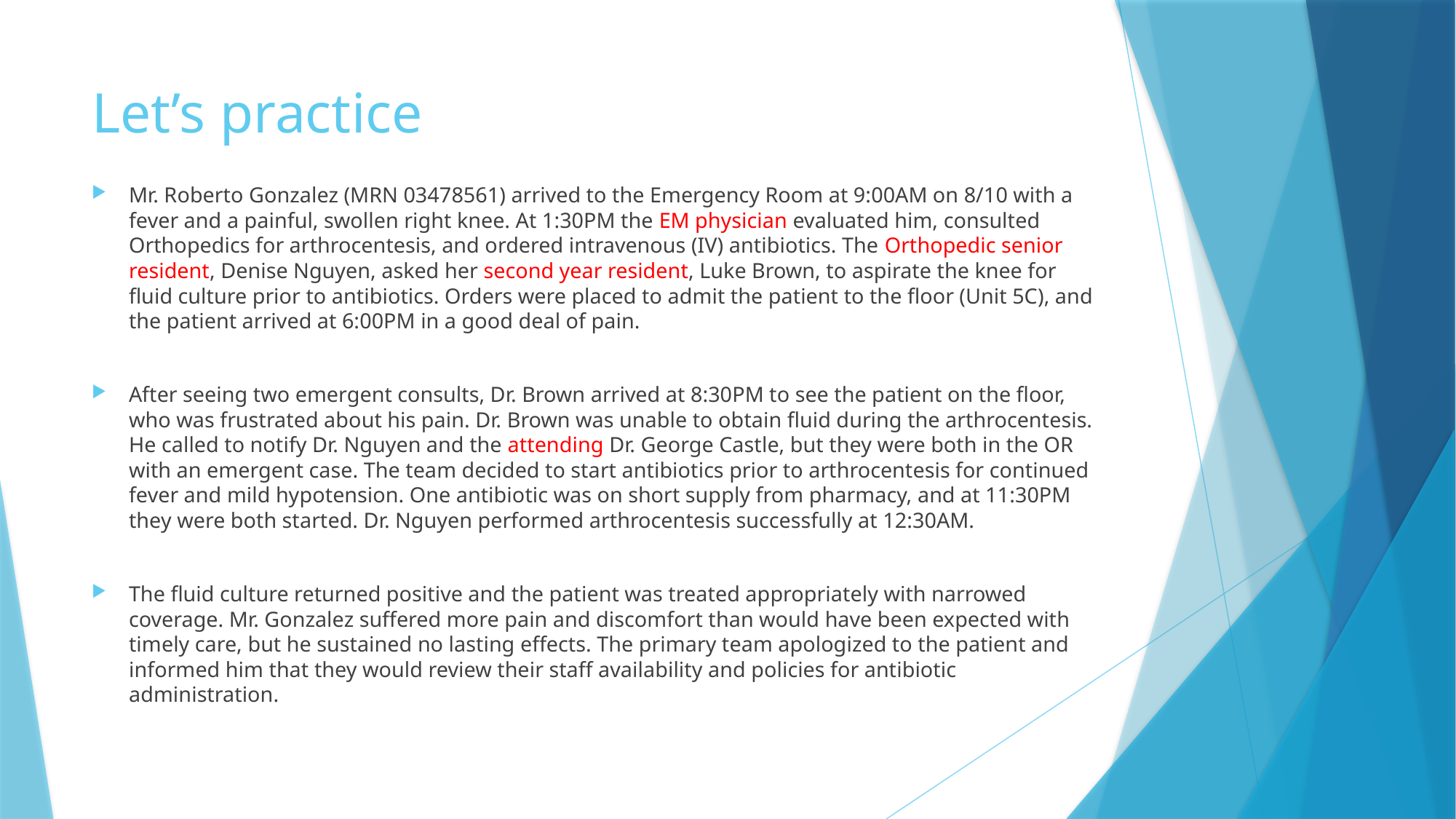

# Let’s practice
Mr. Roberto Gonzalez (MRN 03478561) arrived to the Emergency Room at 9:00AM on 8/10 with a fever and a painful, swollen right knee. At 1:30PM the EM physician evaluated him, consulted Orthopedics for arthrocentesis, and ordered intravenous (IV) antibiotics. The Orthopedic senior resident, Denise Nguyen, asked her second year resident, Luke Brown, to aspirate the knee for fluid culture prior to antibiotics. Orders were placed to admit the patient to the floor (Unit 5C), and the patient arrived at 6:00PM in a good deal of pain.
After seeing two emergent consults, Dr. Brown arrived at 8:30PM to see the patient on the floor, who was frustrated about his pain. Dr. Brown was unable to obtain fluid during the arthrocentesis. He called to notify Dr. Nguyen and the attending Dr. George Castle, but they were both in the OR with an emergent case. The team decided to start antibiotics prior to arthrocentesis for continued fever and mild hypotension. One antibiotic was on short supply from pharmacy, and at 11:30PM they were both started. Dr. Nguyen performed arthrocentesis successfully at 12:30AM.
The fluid culture returned positive and the patient was treated appropriately with narrowed coverage. Mr. Gonzalez suffered more pain and discomfort than would have been expected with timely care, but he sustained no lasting effects. The primary team apologized to the patient and informed him that they would review their staff availability and policies for antibiotic administration.

## Slide 22
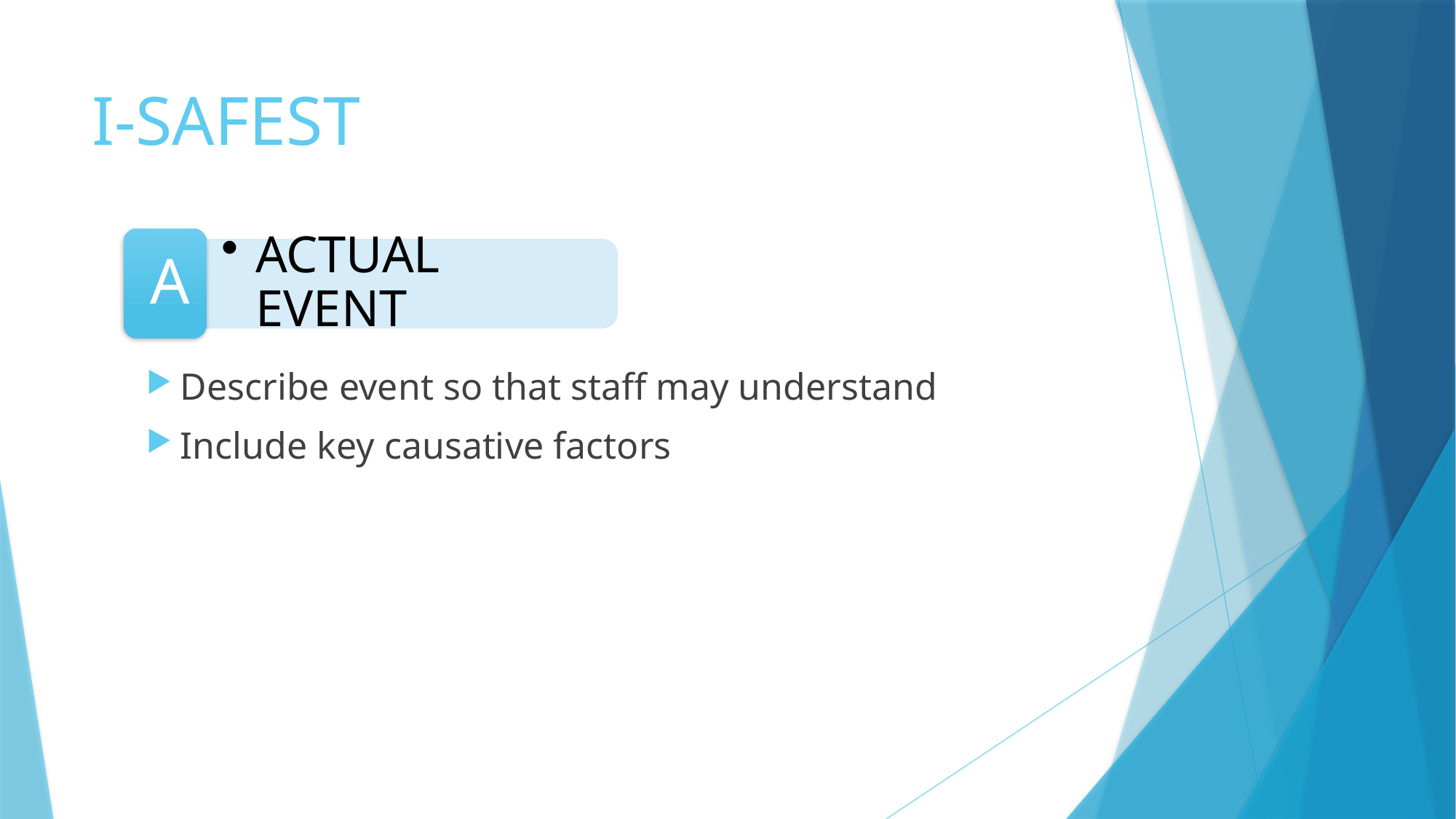

# I-SAFEST
Describe event so that staff may understand
Include key causative factors

## Slide 23
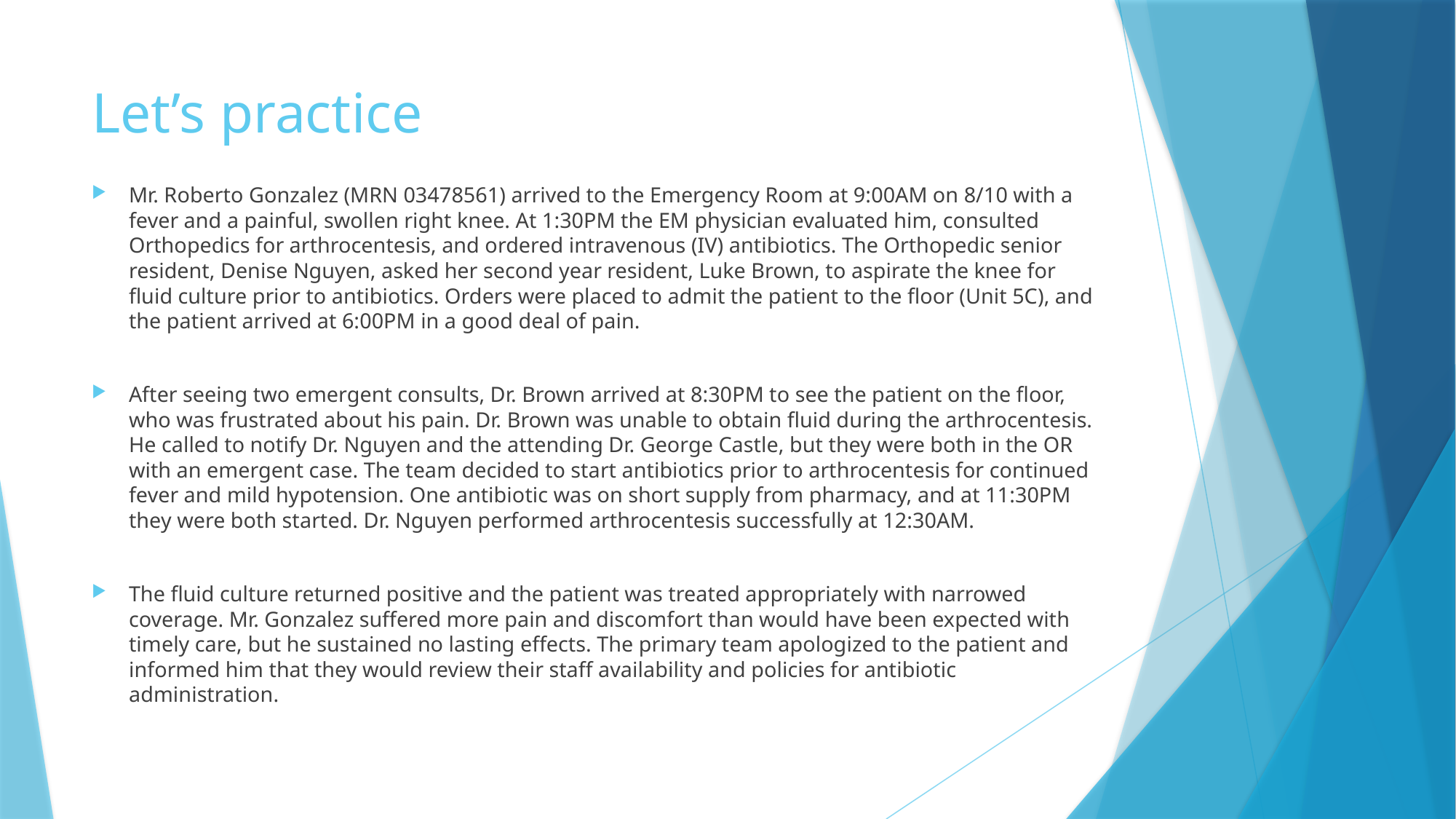

# Let’s practice
Mr. Roberto Gonzalez (MRN 03478561) arrived to the Emergency Room at 9:00AM on 8/10 with a fever and a painful, swollen right knee. At 1:30PM the EM physician evaluated him, consulted Orthopedics for arthrocentesis, and ordered intravenous (IV) antibiotics. The Orthopedic senior resident, Denise Nguyen, asked her second year resident, Luke Brown, to aspirate the knee for fluid culture prior to antibiotics. Orders were placed to admit the patient to the floor (Unit 5C), and the patient arrived at 6:00PM in a good deal of pain.
After seeing two emergent consults, Dr. Brown arrived at 8:30PM to see the patient on the floor, who was frustrated about his pain. Dr. Brown was unable to obtain fluid during the arthrocentesis. He called to notify Dr. Nguyen and the attending Dr. George Castle, but they were both in the OR with an emergent case. The team decided to start antibiotics prior to arthrocentesis for continued fever and mild hypotension. One antibiotic was on short supply from pharmacy, and at 11:30PM they were both started. Dr. Nguyen performed arthrocentesis successfully at 12:30AM.
The fluid culture returned positive and the patient was treated appropriately with narrowed coverage. Mr. Gonzalez suffered more pain and discomfort than would have been expected with timely care, but he sustained no lasting effects. The primary team apologized to the patient and informed him that they would review their staff availability and policies for antibiotic administration.

## Slide 24
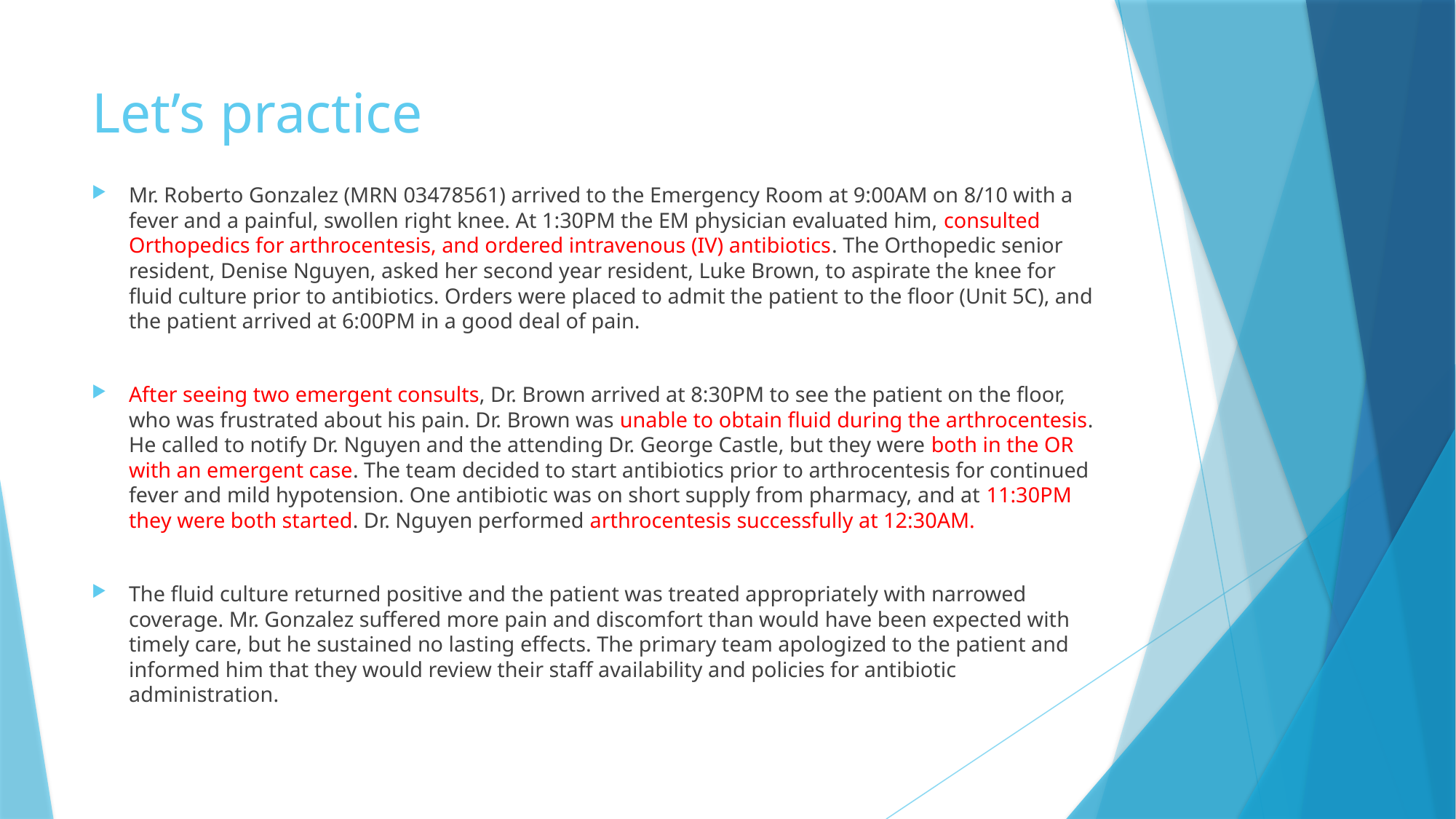

# Let’s practice
Mr. Roberto Gonzalez (MRN 03478561) arrived to the Emergency Room at 9:00AM on 8/10 with a fever and a painful, swollen right knee. At 1:30PM the EM physician evaluated him, consulted Orthopedics for arthrocentesis, and ordered intravenous (IV) antibiotics. The Orthopedic senior resident, Denise Nguyen, asked her second year resident, Luke Brown, to aspirate the knee for fluid culture prior to antibiotics. Orders were placed to admit the patient to the floor (Unit 5C), and the patient arrived at 6:00PM in a good deal of pain.
After seeing two emergent consults, Dr. Brown arrived at 8:30PM to see the patient on the floor, who was frustrated about his pain. Dr. Brown was unable to obtain fluid during the arthrocentesis. He called to notify Dr. Nguyen and the attending Dr. George Castle, but they were both in the OR with an emergent case. The team decided to start antibiotics prior to arthrocentesis for continued fever and mild hypotension. One antibiotic was on short supply from pharmacy, and at 11:30PM they were both started. Dr. Nguyen performed arthrocentesis successfully at 12:30AM.
The fluid culture returned positive and the patient was treated appropriately with narrowed coverage. Mr. Gonzalez suffered more pain and discomfort than would have been expected with timely care, but he sustained no lasting effects. The primary team apologized to the patient and informed him that they would review their staff availability and policies for antibiotic administration.

## Slide 25
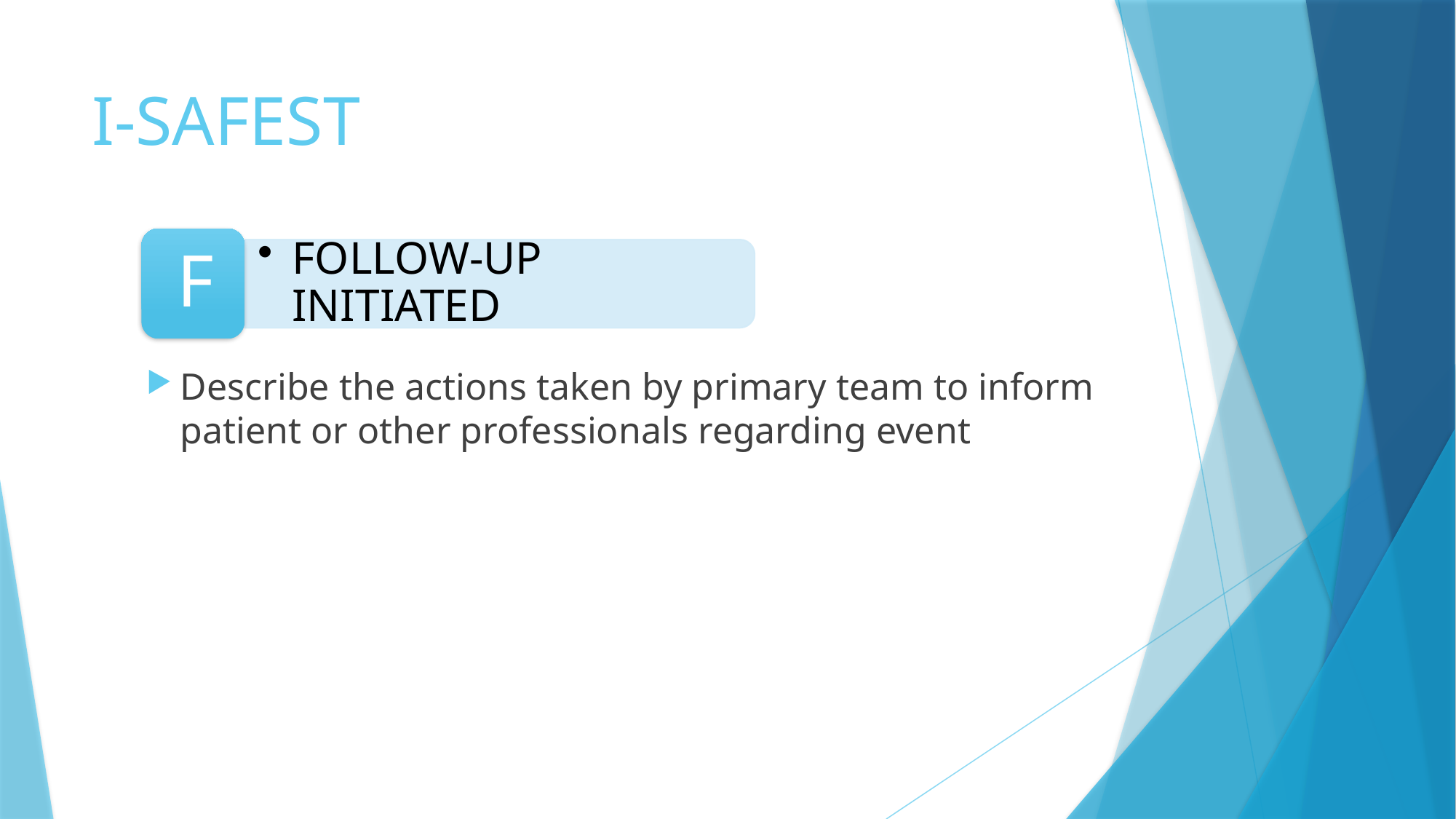

# I-SAFEST
Describe the actions taken by primary team to inform patient or other professionals regarding event

## Slide 26
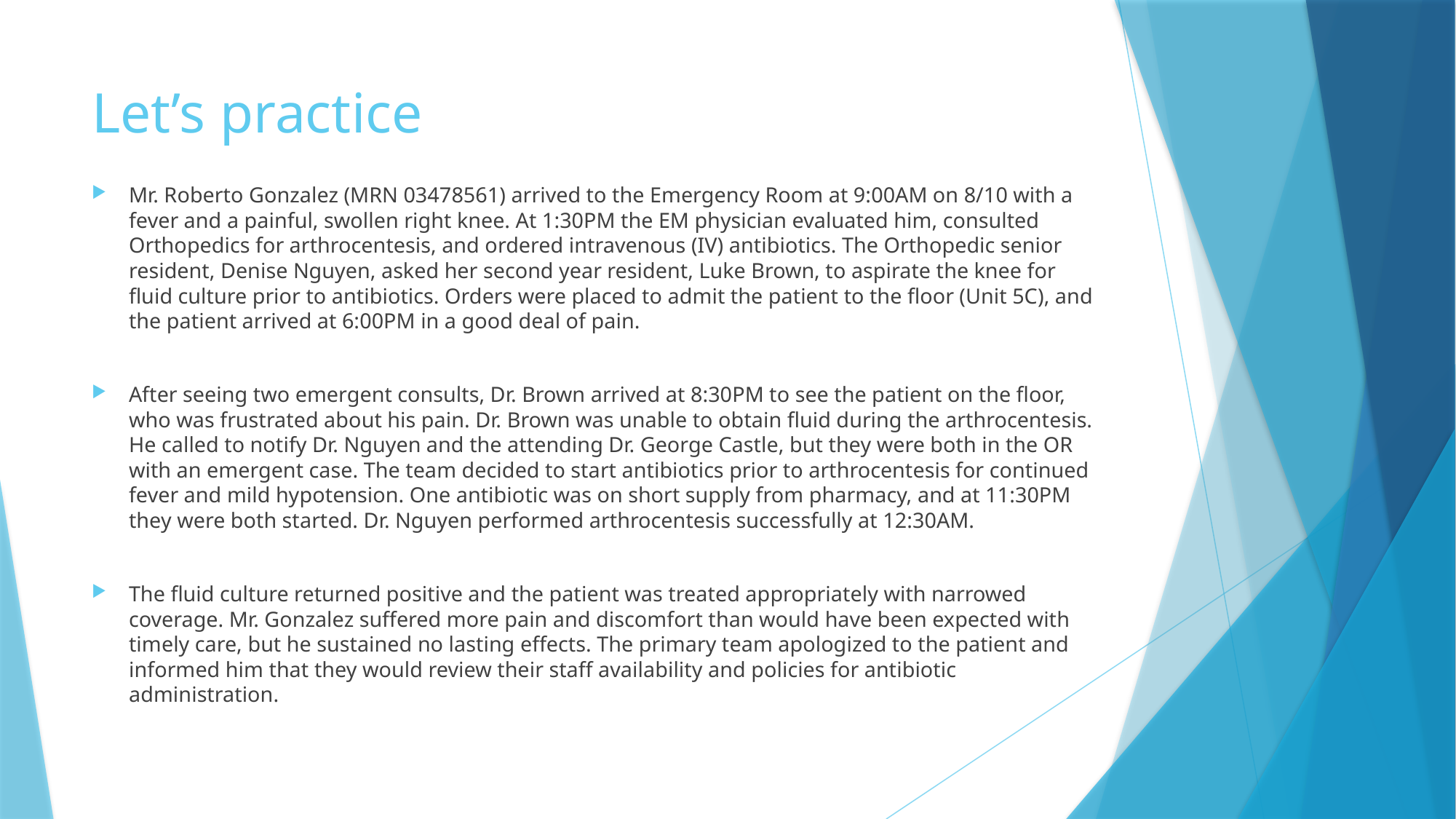

# Let’s practice
Mr. Roberto Gonzalez (MRN 03478561) arrived to the Emergency Room at 9:00AM on 8/10 with a fever and a painful, swollen right knee. At 1:30PM the EM physician evaluated him, consulted Orthopedics for arthrocentesis, and ordered intravenous (IV) antibiotics. The Orthopedic senior resident, Denise Nguyen, asked her second year resident, Luke Brown, to aspirate the knee for fluid culture prior to antibiotics. Orders were placed to admit the patient to the floor (Unit 5C), and the patient arrived at 6:00PM in a good deal of pain.
After seeing two emergent consults, Dr. Brown arrived at 8:30PM to see the patient on the floor, who was frustrated about his pain. Dr. Brown was unable to obtain fluid during the arthrocentesis. He called to notify Dr. Nguyen and the attending Dr. George Castle, but they were both in the OR with an emergent case. The team decided to start antibiotics prior to arthrocentesis for continued fever and mild hypotension. One antibiotic was on short supply from pharmacy, and at 11:30PM they were both started. Dr. Nguyen performed arthrocentesis successfully at 12:30AM.
The fluid culture returned positive and the patient was treated appropriately with narrowed coverage. Mr. Gonzalez suffered more pain and discomfort than would have been expected with timely care, but he sustained no lasting effects. The primary team apologized to the patient and informed him that they would review their staff availability and policies for antibiotic administration.

## Slide 27
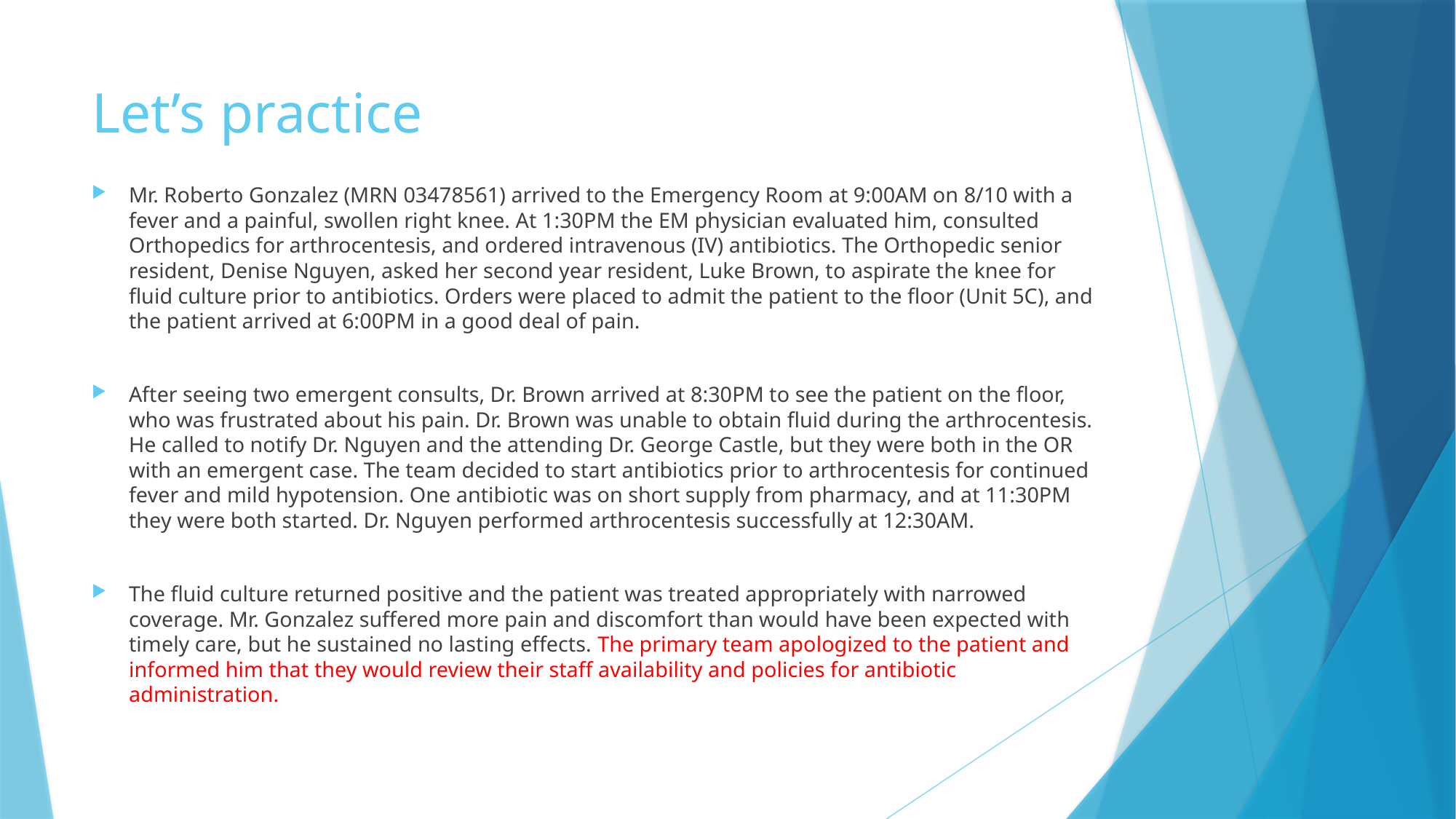

# Let’s practice
Mr. Roberto Gonzalez (MRN 03478561) arrived to the Emergency Room at 9:00AM on 8/10 with a fever and a painful, swollen right knee. At 1:30PM the EM physician evaluated him, consulted Orthopedics for arthrocentesis, and ordered intravenous (IV) antibiotics. The Orthopedic senior resident, Denise Nguyen, asked her second year resident, Luke Brown, to aspirate the knee for fluid culture prior to antibiotics. Orders were placed to admit the patient to the floor (Unit 5C), and the patient arrived at 6:00PM in a good deal of pain.
After seeing two emergent consults, Dr. Brown arrived at 8:30PM to see the patient on the floor, who was frustrated about his pain. Dr. Brown was unable to obtain fluid during the arthrocentesis. He called to notify Dr. Nguyen and the attending Dr. George Castle, but they were both in the OR with an emergent case. The team decided to start antibiotics prior to arthrocentesis for continued fever and mild hypotension. One antibiotic was on short supply from pharmacy, and at 11:30PM they were both started. Dr. Nguyen performed arthrocentesis successfully at 12:30AM.
The fluid culture returned positive and the patient was treated appropriately with narrowed coverage. Mr. Gonzalez suffered more pain and discomfort than would have been expected with timely care, but he sustained no lasting effects. The primary team apologized to the patient and informed him that they would review their staff availability and policies for antibiotic administration.

## Slide 28
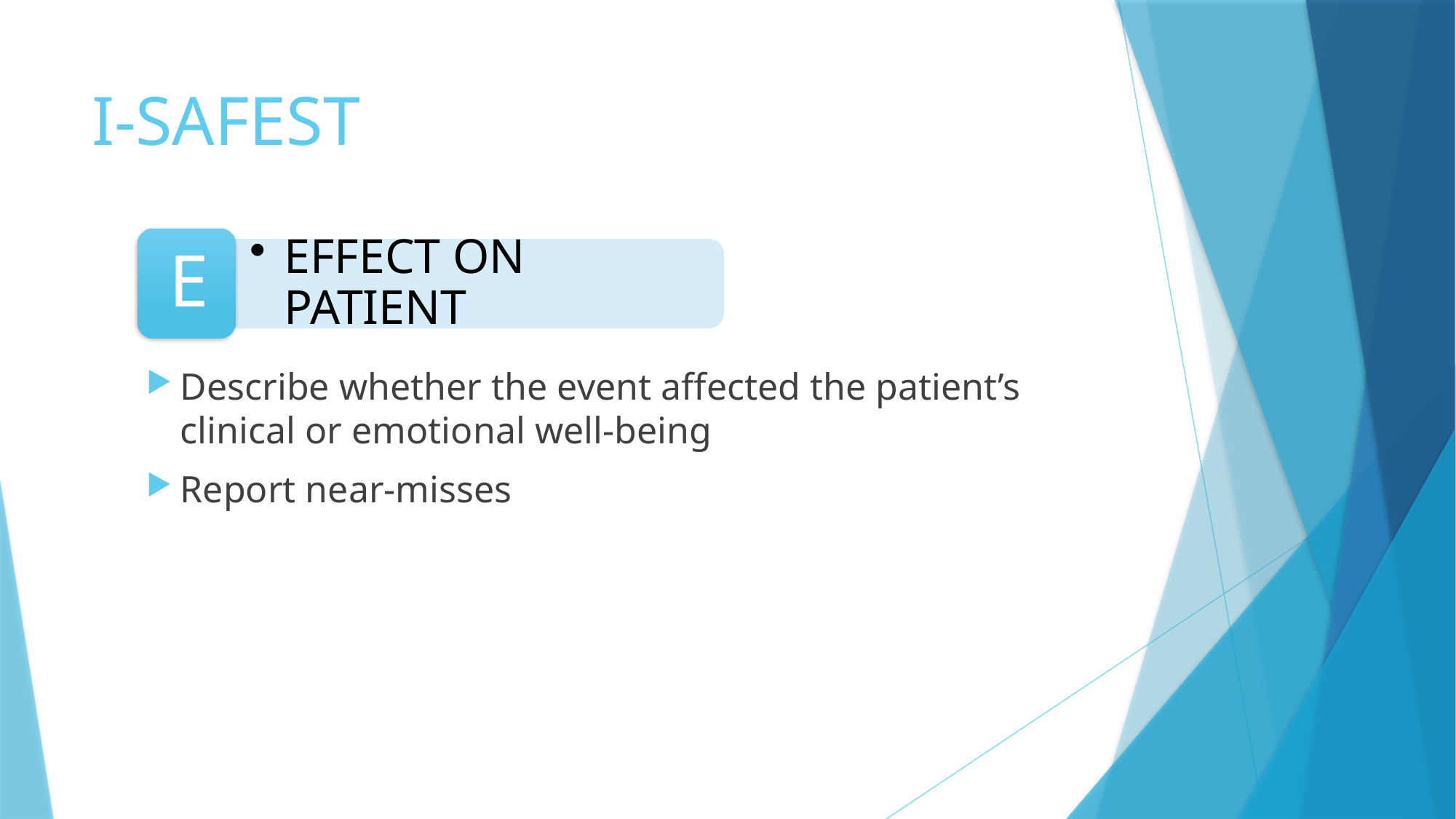

# I-SAFEST
Describe whether the event affected the patient’s clinical or emotional well-being
Report near-misses

## Slide 29
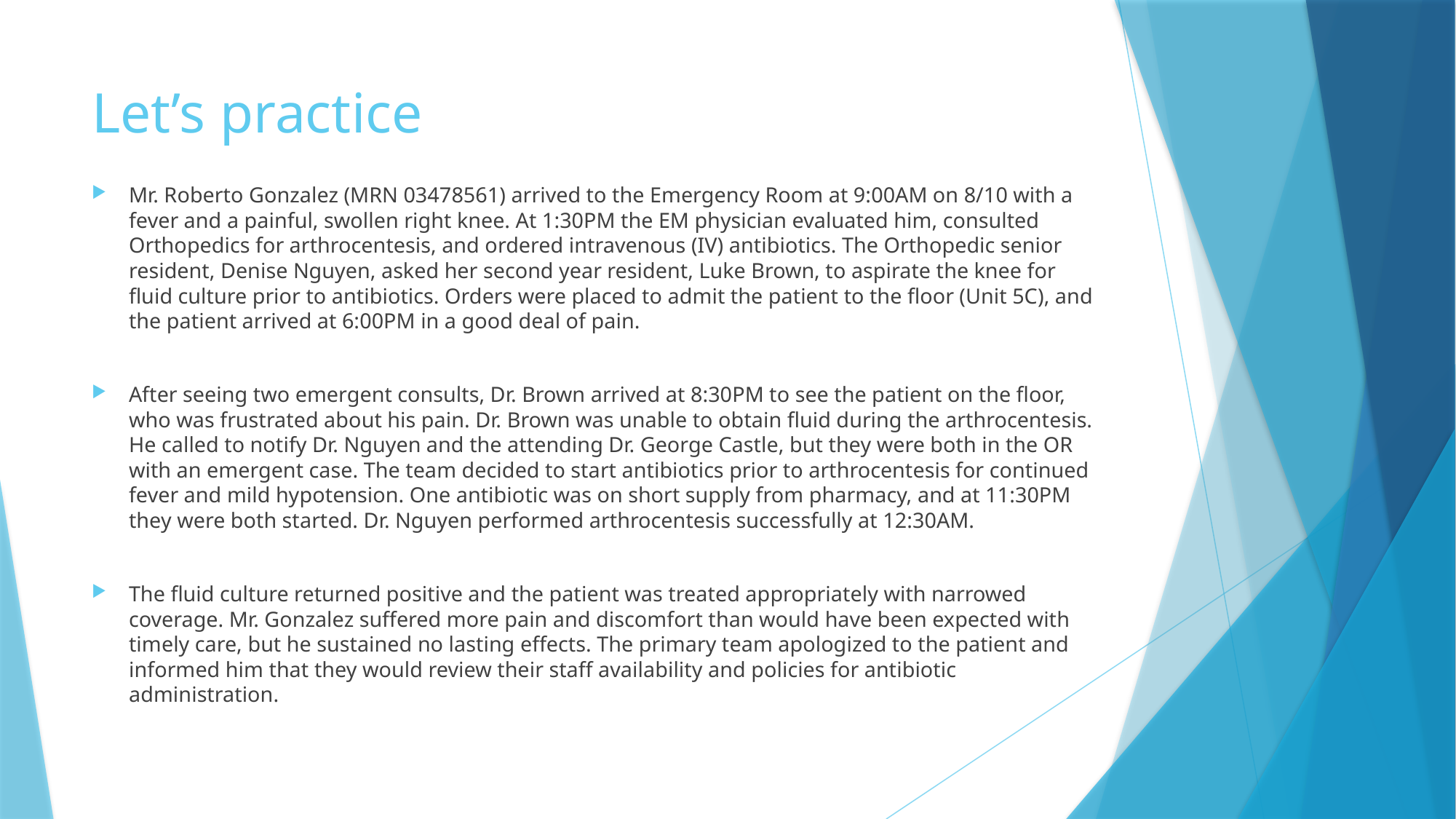

# Let’s practice
Mr. Roberto Gonzalez (MRN 03478561) arrived to the Emergency Room at 9:00AM on 8/10 with a fever and a painful, swollen right knee. At 1:30PM the EM physician evaluated him, consulted Orthopedics for arthrocentesis, and ordered intravenous (IV) antibiotics. The Orthopedic senior resident, Denise Nguyen, asked her second year resident, Luke Brown, to aspirate the knee for fluid culture prior to antibiotics. Orders were placed to admit the patient to the floor (Unit 5C), and the patient arrived at 6:00PM in a good deal of pain.
After seeing two emergent consults, Dr. Brown arrived at 8:30PM to see the patient on the floor, who was frustrated about his pain. Dr. Brown was unable to obtain fluid during the arthrocentesis. He called to notify Dr. Nguyen and the attending Dr. George Castle, but they were both in the OR with an emergent case. The team decided to start antibiotics prior to arthrocentesis for continued fever and mild hypotension. One antibiotic was on short supply from pharmacy, and at 11:30PM they were both started. Dr. Nguyen performed arthrocentesis successfully at 12:30AM.
The fluid culture returned positive and the patient was treated appropriately with narrowed coverage. Mr. Gonzalez suffered more pain and discomfort than would have been expected with timely care, but he sustained no lasting effects. The primary team apologized to the patient and informed him that they would review their staff availability and policies for antibiotic administration.

## Slide 30
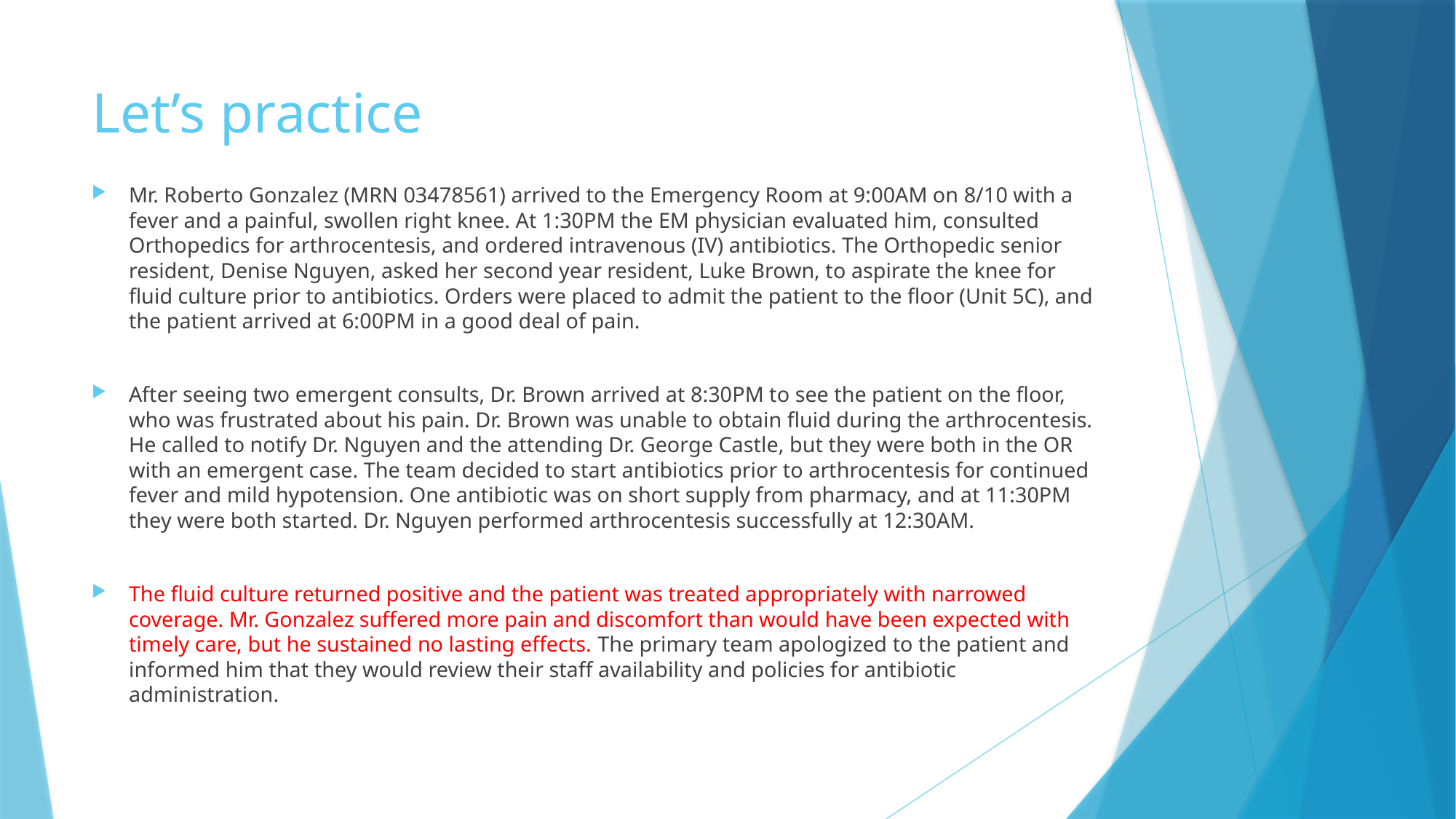

# Let’s practice
Mr. Roberto Gonzalez (MRN 03478561) arrived to the Emergency Room at 9:00AM on 8/10 with a fever and a painful, swollen right knee. At 1:30PM the EM physician evaluated him, consulted Orthopedics for arthrocentesis, and ordered intravenous (IV) antibiotics. The Orthopedic senior resident, Denise Nguyen, asked her second year resident, Luke Brown, to aspirate the knee for fluid culture prior to antibiotics. Orders were placed to admit the patient to the floor (Unit 5C), and the patient arrived at 6:00PM in a good deal of pain.
After seeing two emergent consults, Dr. Brown arrived at 8:30PM to see the patient on the floor, who was frustrated about his pain. Dr. Brown was unable to obtain fluid during the arthrocentesis. He called to notify Dr. Nguyen and the attending Dr. George Castle, but they were both in the OR with an emergent case. The team decided to start antibiotics prior to arthrocentesis for continued fever and mild hypotension. One antibiotic was on short supply from pharmacy, and at 11:30PM they were both started. Dr. Nguyen performed arthrocentesis successfully at 12:30AM.
The fluid culture returned positive and the patient was treated appropriately with narrowed coverage. Mr. Gonzalez suffered more pain and discomfort than would have been expected with timely care, but he sustained no lasting effects. The primary team apologized to the patient and informed him that they would review their staff availability and policies for antibiotic administration.

## Slide 31
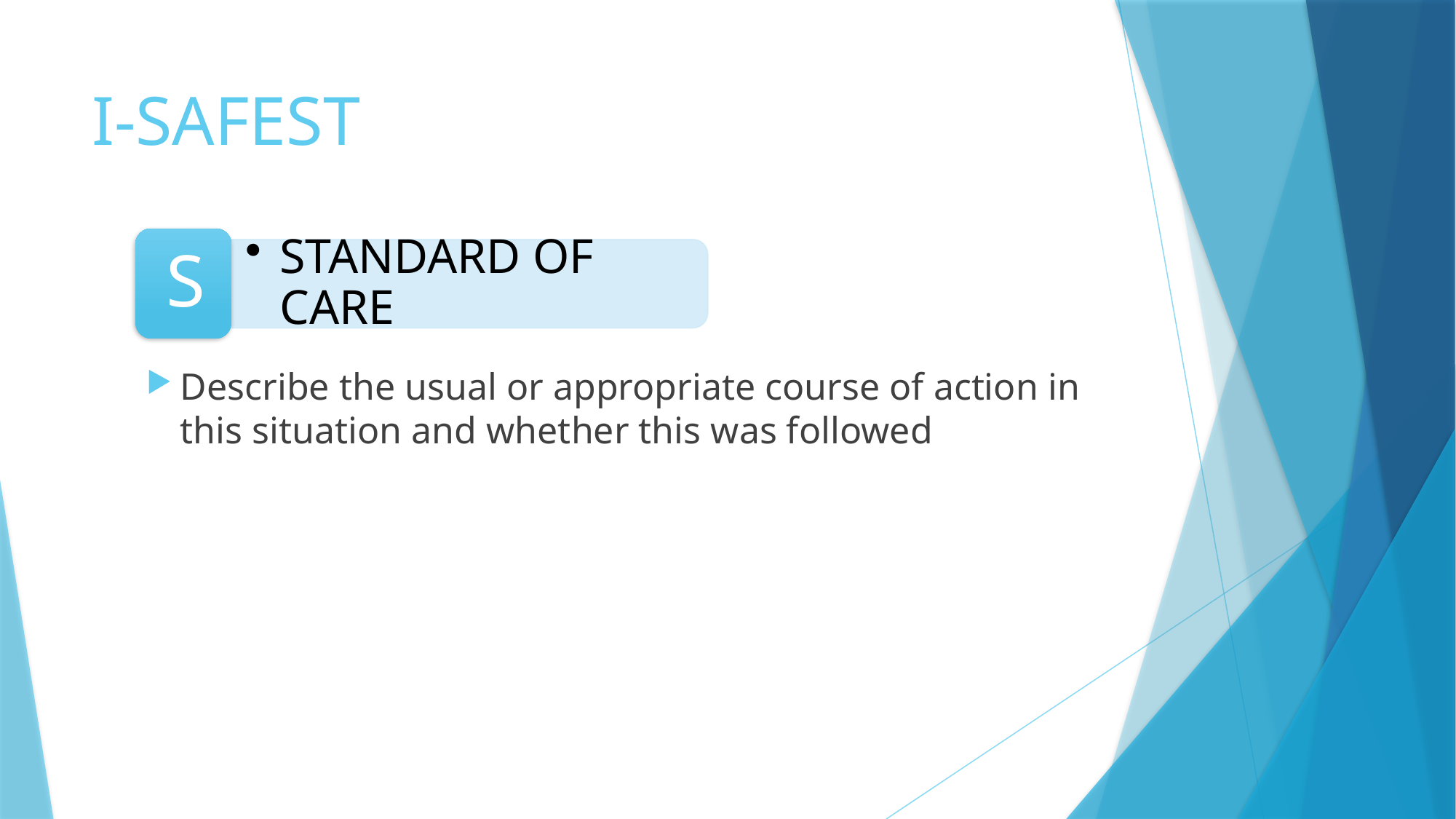

# I-SAFEST
Describe the usual or appropriate course of action in this situation and whether this was followed

## Slide 32
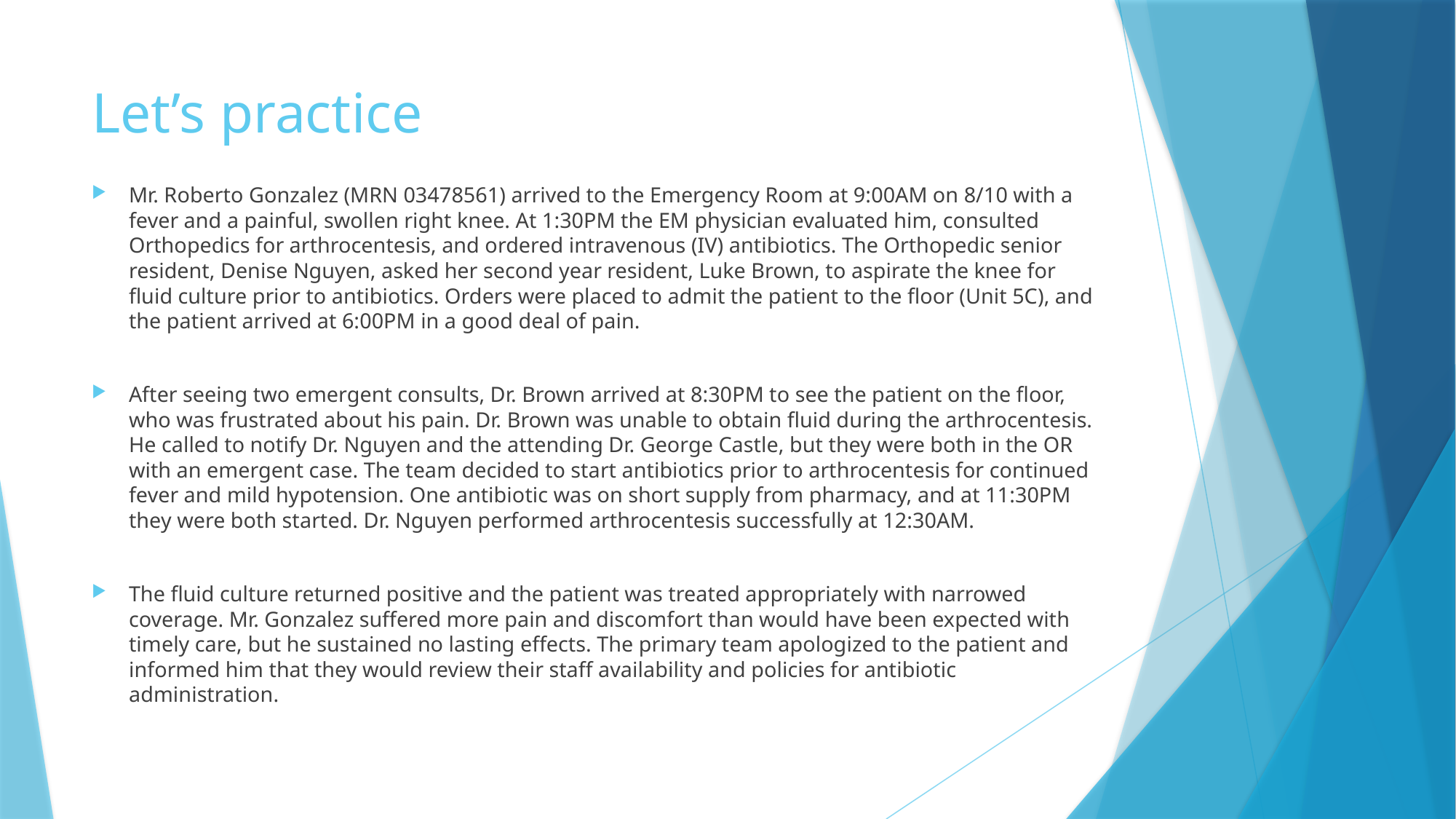

# Let’s practice
Mr. Roberto Gonzalez (MRN 03478561) arrived to the Emergency Room at 9:00AM on 8/10 with a fever and a painful, swollen right knee. At 1:30PM the EM physician evaluated him, consulted Orthopedics for arthrocentesis, and ordered intravenous (IV) antibiotics. The Orthopedic senior resident, Denise Nguyen, asked her second year resident, Luke Brown, to aspirate the knee for fluid culture prior to antibiotics. Orders were placed to admit the patient to the floor (Unit 5C), and the patient arrived at 6:00PM in a good deal of pain.
After seeing two emergent consults, Dr. Brown arrived at 8:30PM to see the patient on the floor, who was frustrated about his pain. Dr. Brown was unable to obtain fluid during the arthrocentesis. He called to notify Dr. Nguyen and the attending Dr. George Castle, but they were both in the OR with an emergent case. The team decided to start antibiotics prior to arthrocentesis for continued fever and mild hypotension. One antibiotic was on short supply from pharmacy, and at 11:30PM they were both started. Dr. Nguyen performed arthrocentesis successfully at 12:30AM.
The fluid culture returned positive and the patient was treated appropriately with narrowed coverage. Mr. Gonzalez suffered more pain and discomfort than would have been expected with timely care, but he sustained no lasting effects. The primary team apologized to the patient and informed him that they would review their staff availability and policies for antibiotic administration.

## Slide 33
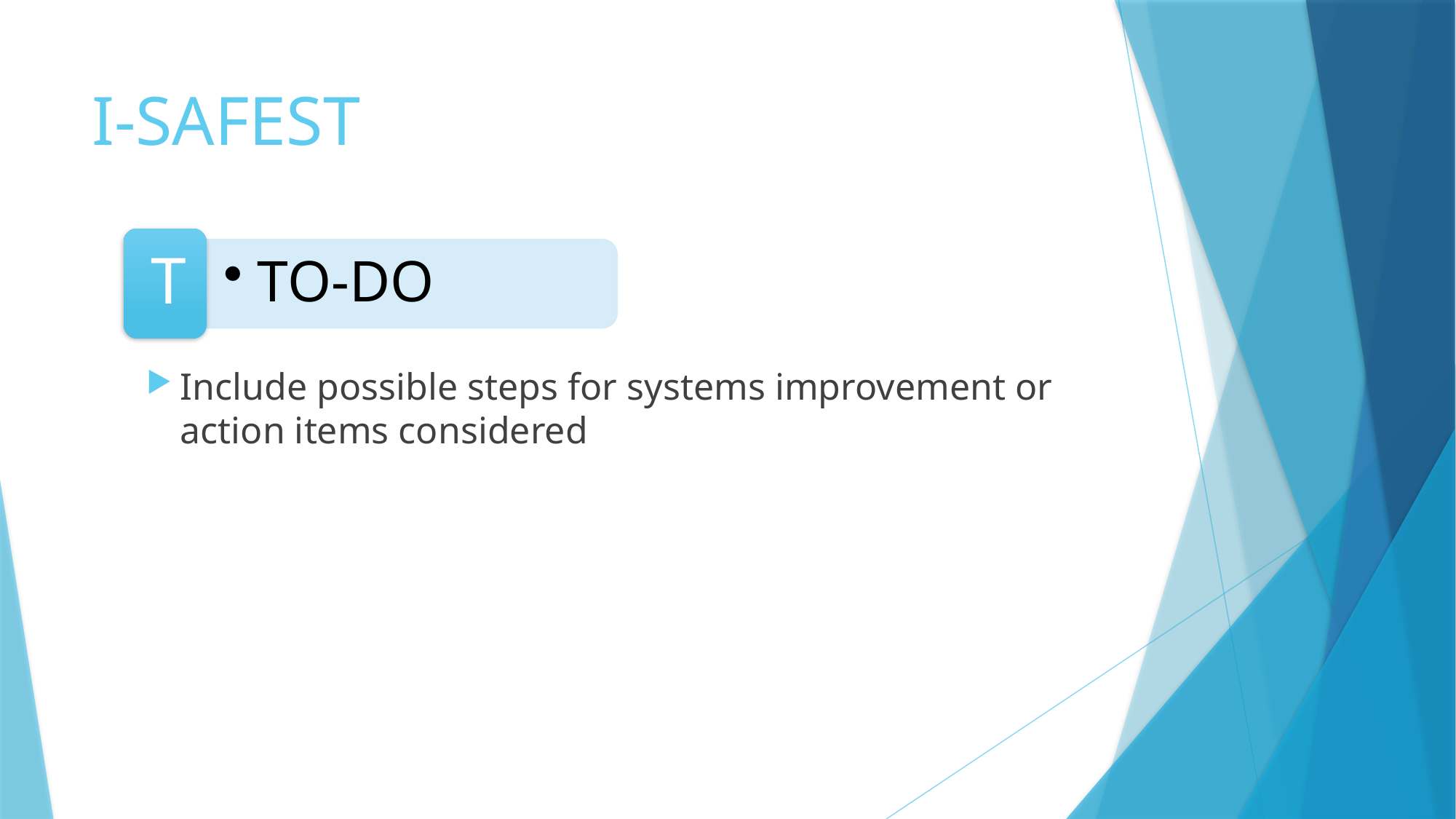

# I-SAFEST
Include possible steps for systems improvement or action items considered

## Slide 34
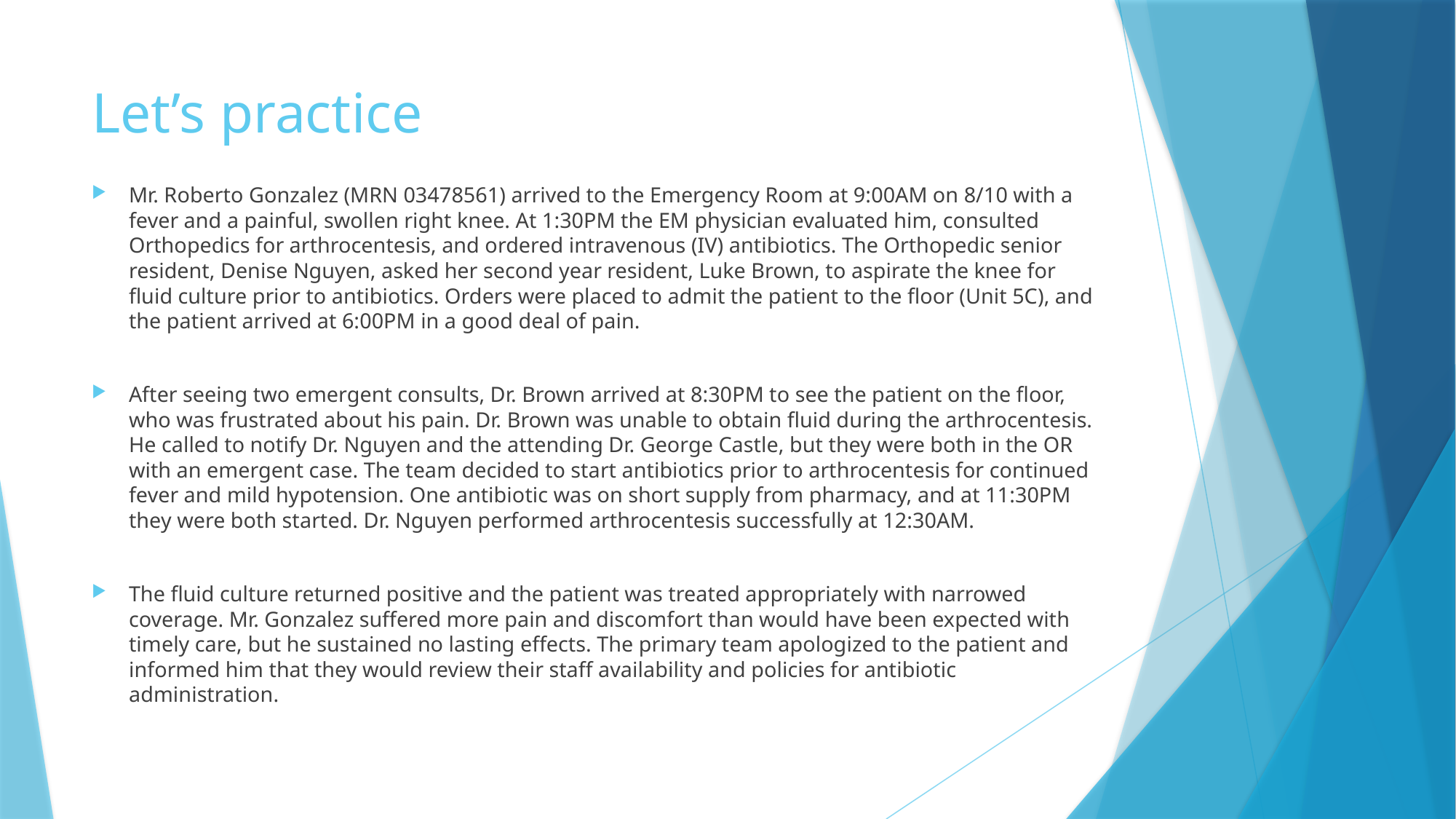

# Let’s practice
Mr. Roberto Gonzalez (MRN 03478561) arrived to the Emergency Room at 9:00AM on 8/10 with a fever and a painful, swollen right knee. At 1:30PM the EM physician evaluated him, consulted Orthopedics for arthrocentesis, and ordered intravenous (IV) antibiotics. The Orthopedic senior resident, Denise Nguyen, asked her second year resident, Luke Brown, to aspirate the knee for fluid culture prior to antibiotics. Orders were placed to admit the patient to the floor (Unit 5C), and the patient arrived at 6:00PM in a good deal of pain.
After seeing two emergent consults, Dr. Brown arrived at 8:30PM to see the patient on the floor, who was frustrated about his pain. Dr. Brown was unable to obtain fluid during the arthrocentesis. He called to notify Dr. Nguyen and the attending Dr. George Castle, but they were both in the OR with an emergent case. The team decided to start antibiotics prior to arthrocentesis for continued fever and mild hypotension. One antibiotic was on short supply from pharmacy, and at 11:30PM they were both started. Dr. Nguyen performed arthrocentesis successfully at 12:30AM.
The fluid culture returned positive and the patient was treated appropriately with narrowed coverage. Mr. Gonzalez suffered more pain and discomfort than would have been expected with timely care, but he sustained no lasting effects. The primary team apologized to the patient and informed him that they would review their staff availability and policies for antibiotic administration.

## Slide 35
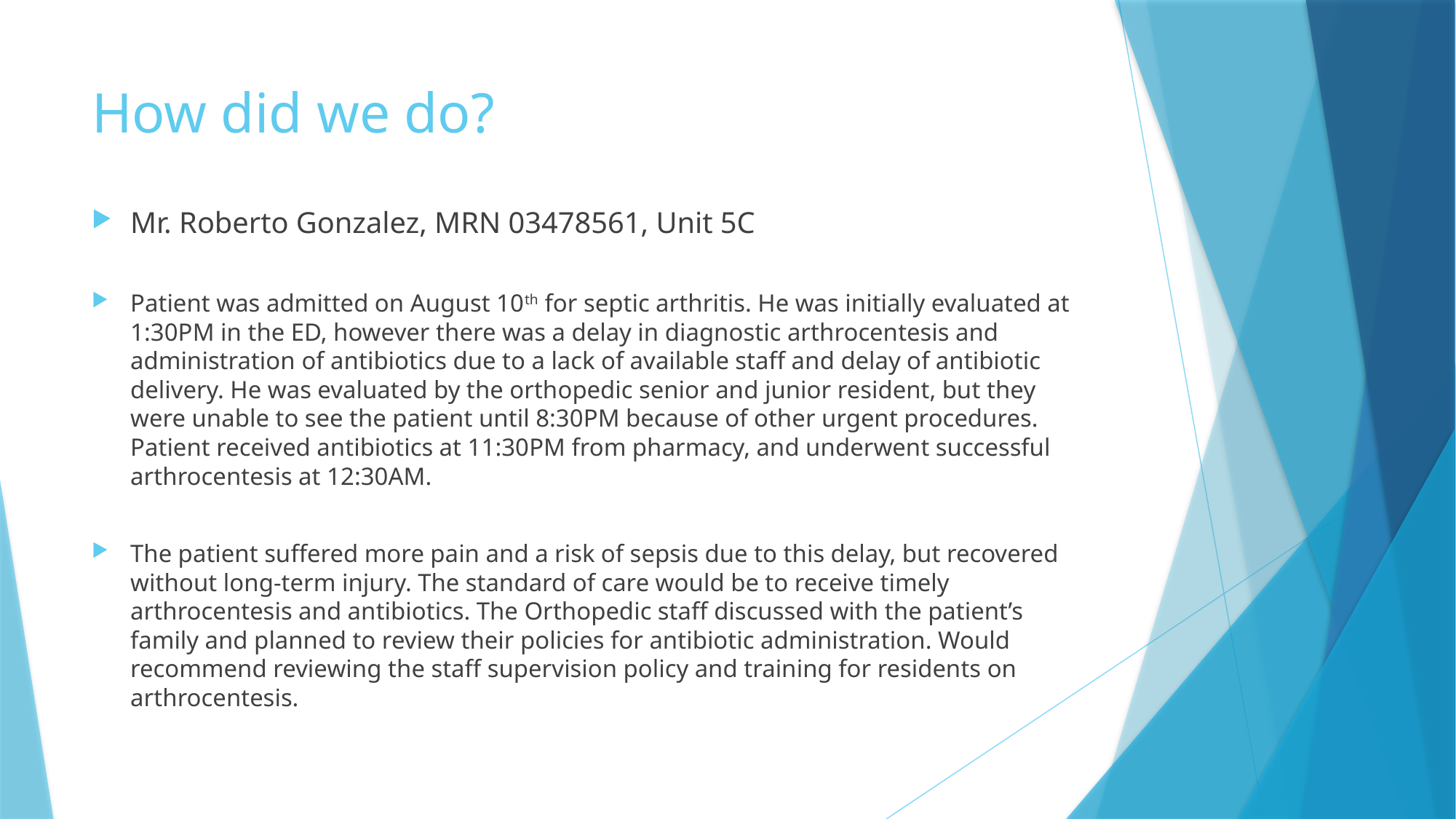

# How did we do?
Mr. Roberto Gonzalez, MRN 03478561, Unit 5C
Patient was admitted on August 10th for septic arthritis. He was initially evaluated at 1:30PM in the ED, however there was a delay in diagnostic arthrocentesis and administration of antibiotics due to a lack of available staff and delay of antibiotic delivery. He was evaluated by the orthopedic senior and junior resident, but they were unable to see the patient until 8:30PM because of other urgent procedures. Patient received antibiotics at 11:30PM from pharmacy, and underwent successful arthrocentesis at 12:30AM.
The patient suffered more pain and a risk of sepsis due to this delay, but recovered without long-term injury. The standard of care would be to receive timely arthrocentesis and antibiotics. The Orthopedic staff discussed with the patient’s family and planned to review their policies for antibiotic administration. Would recommend reviewing the staff supervision policy and training for residents on arthrocentesis.

## Slide 36
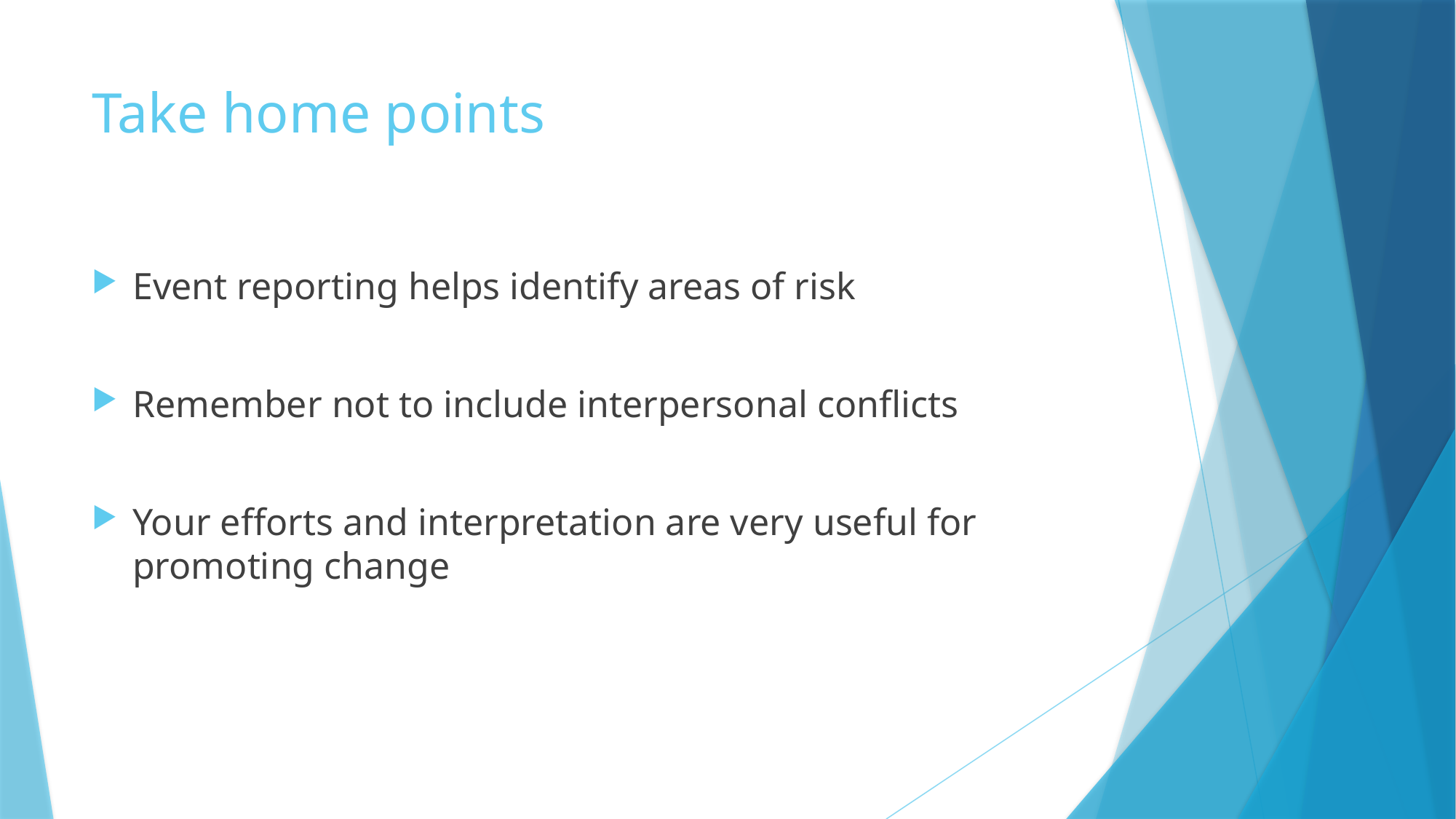

# Take home points
Event reporting helps identify areas of risk
Remember not to include interpersonal conflicts
Your efforts and interpretation are very useful for promoting change

## Slide 37
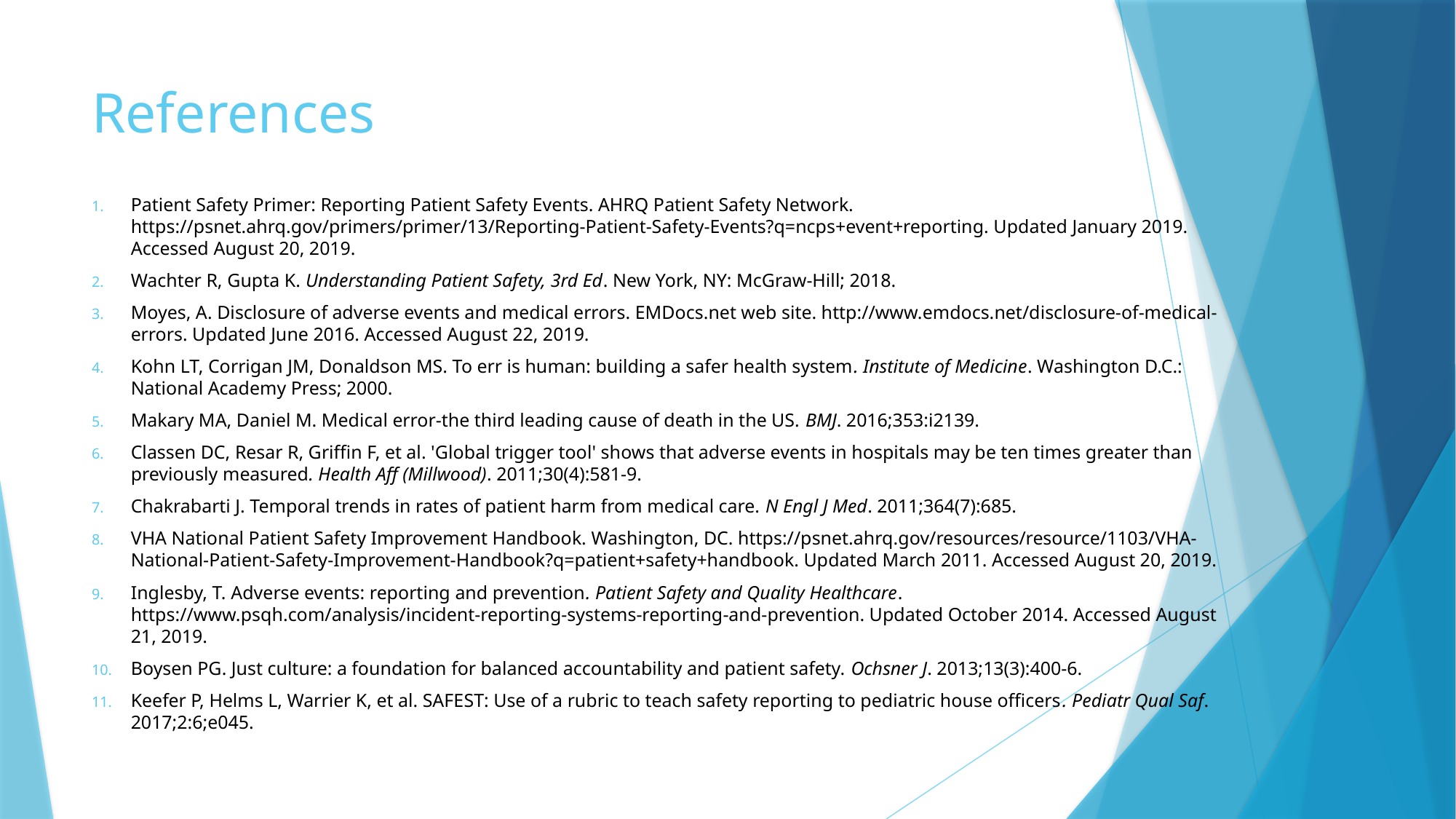

# References
Patient Safety Primer: Reporting Patient Safety Events. AHRQ Patient Safety Network. https://psnet.ahrq.gov/primers/primer/13/Reporting-Patient-Safety-Events?q=ncps+event+reporting. Updated January 2019. Accessed August 20, 2019.
Wachter R, Gupta K. Understanding Patient Safety, 3rd Ed. New York, NY: McGraw-Hill; 2018.
Moyes, A. Disclosure of adverse events and medical errors. EMDocs.net web site. http://www.emdocs.net/disclosure-of-medical-errors. Updated June 2016. Accessed August 22, 2019.
Kohn LT, Corrigan JM, Donaldson MS. To err is human: building a safer health system. Institute of Medicine. Washington D.C.: National Academy Press; 2000.
Makary MA, Daniel M. Medical error-the third leading cause of death in the US. BMJ. 2016;353:i2139.
Classen DC, Resar R, Griffin F, et al. 'Global trigger tool' shows that adverse events in hospitals may be ten times greater than previously measured. Health Aff (Millwood). 2011;30(4):581-9.
Chakrabarti J. Temporal trends in rates of patient harm from medical care. N Engl J Med. 2011;364(7):685.
VHA National Patient Safety Improvement Handbook. Washington, DC. https://psnet.ahrq.gov/resources/resource/1103/VHA-National-Patient-Safety-Improvement-Handbook?q=patient+safety+handbook. Updated March 2011. Accessed August 20, 2019.
Inglesby, T. Adverse events: reporting and prevention. Patient Safety and Quality Healthcare. https://www.psqh.com/analysis/incident-reporting-systems-reporting-and-prevention. Updated October 2014. Accessed August 21, 2019.
Boysen PG. Just culture: a foundation for balanced accountability and patient safety. Ochsner J. 2013;13(3):400-6.
Keefer P, Helms L, Warrier K, et al. SAFEST: Use of a rubric to teach safety reporting to pediatric house officers. Pediatr Qual Saf. 2017;2:6;e045.

## Slide 38
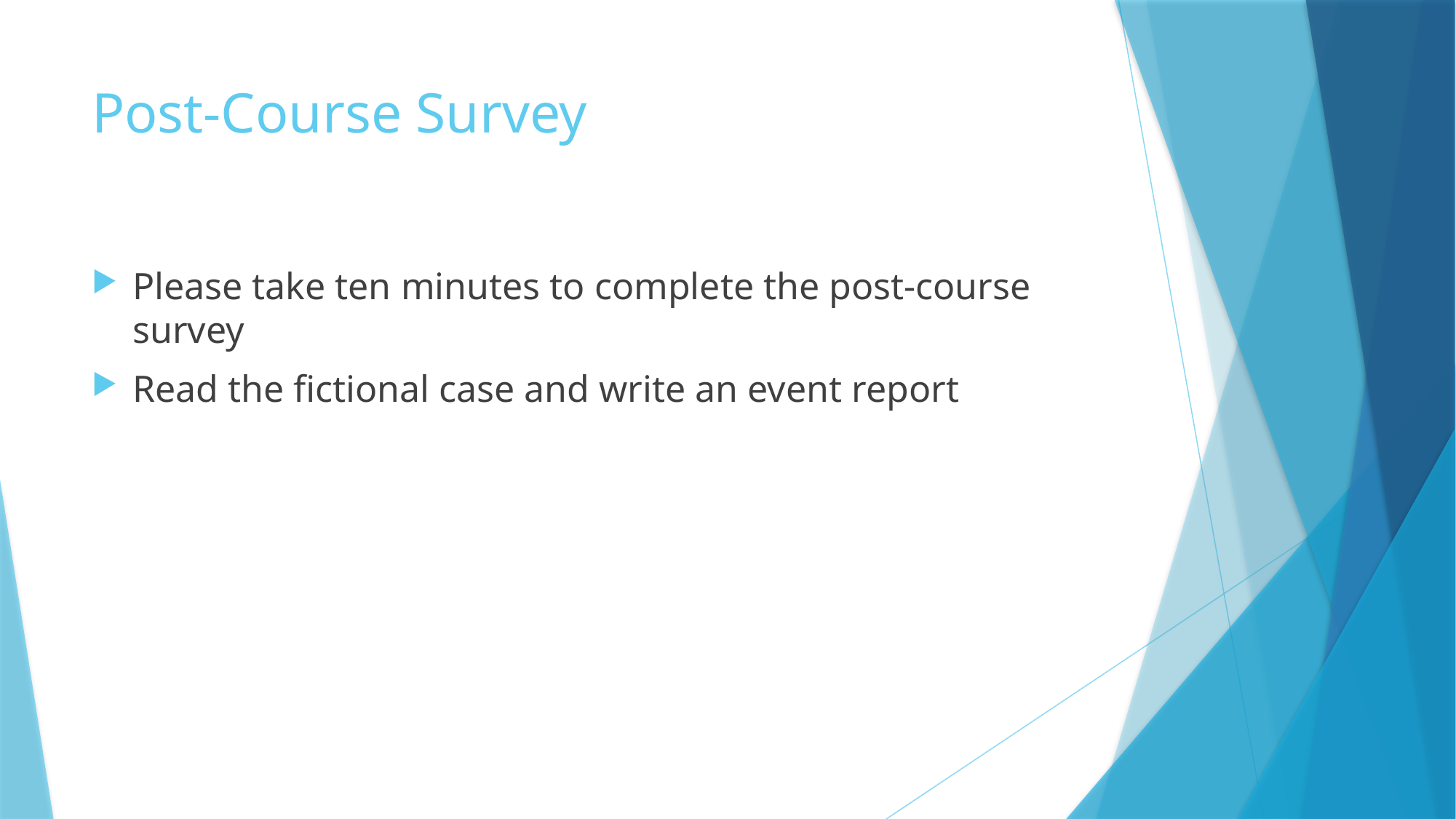

# Post-Course Survey
Please take ten minutes to complete the post-course survey
Read the fictional case and write an event report

## Slide 39
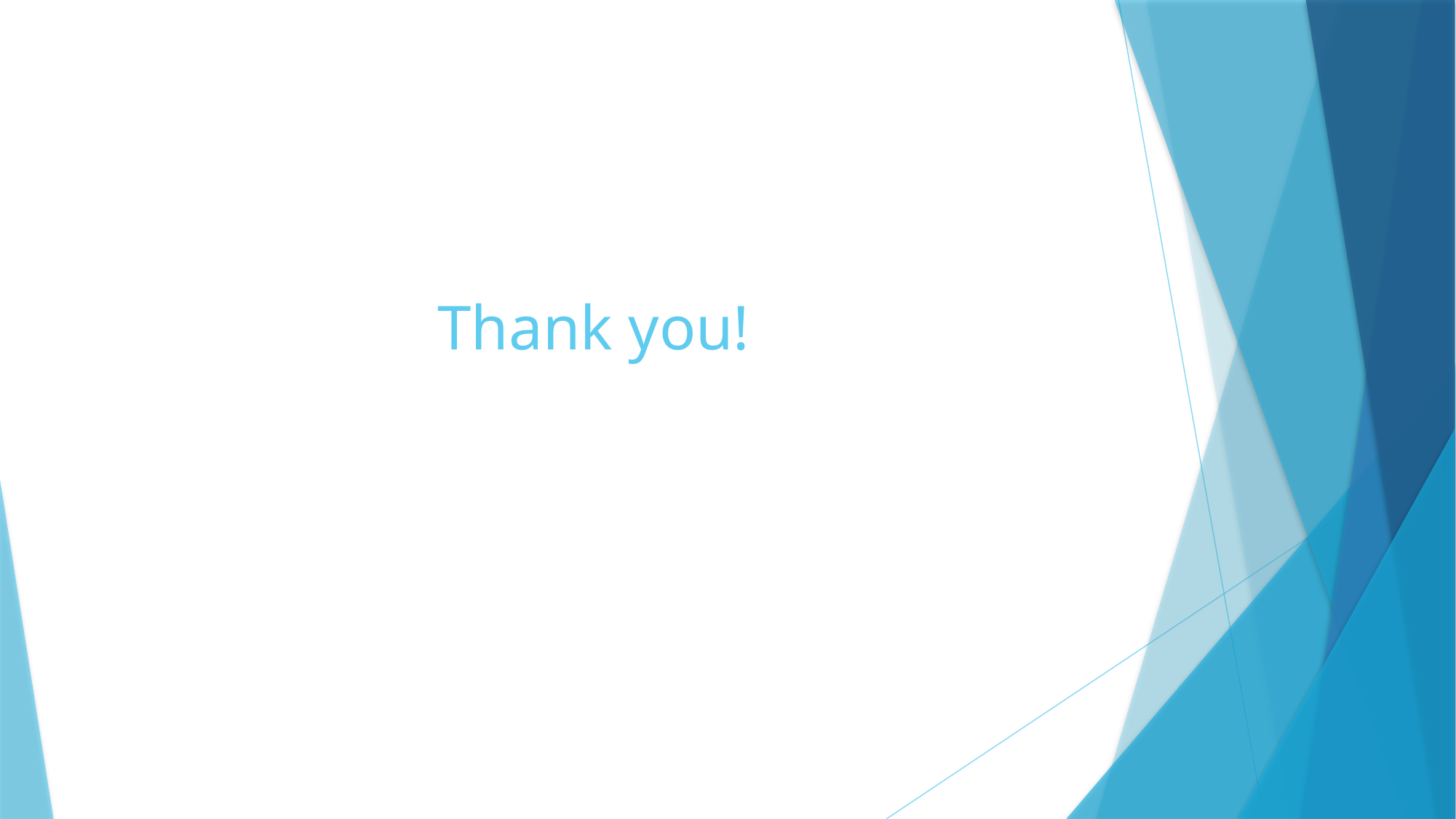

# Thank you!
