# Supplementary material for: Use of a Rubric to Improve the Quality of Internal Medicine Resident Event Reporting
Source: MedEdPORTAL. 2021 Oct 11;17:11189. doi: 10.15766/mep_2374-8265.11189 (PMC8502786; doi:10.15766/mep_2374-8265.11189)
Supplement: Supplementary file 1 — Pretraining Survey.docxPosttraining Survey.docxResident Training Module.pptxInstructor Guide.docxResident Training Module Script.docxI-SAFEST Scoring Sheet.docx [file mep_2374-8265.11189-s001.zip › E. Resident Training Module Script.docx]

**Appendix E- Presentation Template Script**

Below is a script to accompany the slide show template (Appendix C). We recommend reviewing this script before facilitating the presentation.

**Introduction Slide #1**

- “Welcome to Event Reporting: A guide to effective reporting habits.” Introduce yourself and any QI/PS qualifications. “This training will be interactive. We will be reviewing errors and adverse events that you have experienced working with patients and discussing what we can do to make medical care safer at our hospitals. Please remember that all personal information shared today is confidential. This session will include 10 minutes for a short survey and practice event report, followed by 40 minutes of discussion about the value of reporting, and we will write an event report as a group. Afterwards, you will have 10 minutes to complete a post-course survey and to practice what you have learned by completing an event report for a fictional case. Please be respectful of your patients and colleagues. Remember that we as providers often become the second victim in medical error, meaning that the guilt or loss in a medical error affects us as well.”
- “Do you have any questions before we begin?”

**Introduction Slide #2**

- Pass out the pre-training survey (Appendix A). “Please take ten minutes to complete the pre-course survey. Read the fictional patient case and write a brief report of this event using the information you would deem necessary for the patient safety team at your hospital.”
- *(Allow 10 minutes for survey completion. If survey is completed in less than 10 minutes, engage residents in a discussion about their experience writing the event report)*
- Example discussion questions:
  - “What did you find challenging about writing the event report?”
  - “How confident are you that your report includes all the information patient safety officers need?”

**Introduction Slide #3**

- “The objectives for today’s conference are to 1) understand the process and value of event reporting for our patients, 2) write an effective report using the I-SAFEST model, and 3) improve your confidence in your ability to report the events you encounter.”

**Discussion Slide #4**

- Question: “What types of errors or patient safety events have you seen in your experience with patients?”
  - Probing Questions:
    - “Did these errors result in patient harm?”
    - “Did you report them? Why or why not?”
    - “Was it difficult to review or disclose of these errors in your experience?”
- If there are no responses, consider probing for types of events. For example:
  - “Are there times when your patients have had delays of care, or did not receive lab work on time? Are there times when your patients have almost received the wrong medication, or you have entered the wrong chart by accident due to a name mix-up?
- If residents volunteer an example, encourage discussion from the group. Example:
  - “Your example of a catheter-associated UTI due to a prolonged catheter use reminds me of a similar situation that I encountered with a patient. Have the rest of you ever had a patient with a catheter-associated UTI? Do you think it could have been prevented in your case?”
  - “That’s a good example of an error that thankfully did not reach the patient. Have any of you had similar situations with medication administration?”
- Please provide additional examples from the facilitator’s perspective. It is helpful for residents to hear about error from the chief resident or faculty. For example:
  - “I recently encountered an error that involved ___, during which my patient suffered no harm, but I was very worried that he or she would be affected by ___.”
- “Now that we’ve shared some of our experiences, let’s put them into a framework for event reporting.”
- *(Allow 5 minutes for discussion)*

**Didactic and** **Discussion Slide #5**

- “What is a patient safety event? A patient safety event is defined as an event or action that either leads to, or has the potential to lead to, a worsened patient outcome. There are several different types of events that occur in the hospital. These can be:
- Medication errors:
- These may be reported as a delay in receipt of medication, such as an issue with obtaining the medication from pharmacy or administration. These may also be categorized by the incorrect patient, dosage, or administration of medication, among others.
- Have you encountered a medication error with one of your patients? *(Pause for discussion).*
- Clinical care:
- These errors may be related to a delay, such as difficulty in transferring patient from the Emergency Room to the inpatient floor, or to higher level of care. They may also result from an incorrect diagnosis, a failure to recognize the correct diagnosis, or an incorrect treatment.
- Have your patients encountered a delay of care or the incorrect diagnosis? *(Pause for discussion).*
- Equipment issues:
- These may be related to faulty surgical instruments or mislabeled specimens. These are important to recognize because event reports allow us to look into the inventory and handling of equipment and specimens.
- Have you encountered a problem with labeled specimens or broken equipment? Was it reported? *(Pause for discussion).*
- Surgical or procedural errors:
- These may be due to wrong site surgeries, retained objects, the lack of timeout, or issues during a procedure.
- Have you experienced errors such as this in your patient care? *(Pause for discussion).*
- Communication errors:
- These errors may include communication errors from physician to physician, physician to nurse, or provider to patient, among others. Communication errors often occur during handoffs.
- Have you ever experienced a miscommunication during a handoff? *(Pause for discussion).*
- Hospital-acquired infections include catheter-associated, central line infections, and hospital acquired pneumonia, among others.”
- Have you ever had a patient with a hospital-acquired infection? Do you think it could have been avoided during the patient’s care? *(Pause for discussion).*

*- (Allow 5 mins for discussion regarding different types of safety events)*

**Didactic and Discussion Slide #6**

- “How do we categorize or think about these patient safety events? Let’s define a few terms that are often used in the patient safety domain.
- Medical Error:
- This is a broad term referring to an act of either commission (doing something wrong) or omission (failing to do something right) that may expose a patient to a potentially hazardous situation.
- Adverse Event:
- This is an unintended physical injury resulting from or contributed to by medical care (including the absence of treatment) that requires additional monitoring, treatment, hospitalization, or results in death. This has also been defined as an injury that was caused by medical management, rather than the underlying disease. These adverse events can be classified as either unpreventable or preventable, meaning that they could have been avoided by the current standard of care.
- A classic example involves allergies to medications: if a patient has never received a medication before and has an allergic reaction, this would be unpreventable adverse event. If the patient had an allergy warning for that medication listed in the chart and still received it, this would be preventable adverse event.
  - Can you think of other types of adverse events that are unpreventable? *(Pause for discussion).*
- Near Miss:
- A near miss is an event that may have had adverse consequences but did not, and was indistinguishable apart from outcome. Our goal is to seek out those events which are preventable, as well as near misses, to prevent future events.”
- Have you caught or found an error before it reached your patient? Did you report the near miss? *(Pause for discussion).*

**Didactic and Discussion Slide #7**

- - “Why should we report these events? Over the past twenty years, there has been increasing awareness for the burden of medical error in the United States. Per the British Medical Journal’s report in 2013, over 250 thousand deaths per year can be attributed to medical error in the United States. This places medical error as the third leading cause of death in the United States behind heart disease and cancer.
    - Do you think this is an over- or under estimate? Why or why not?” *(Pause for discussion).*

**Didactic Slide #8**

- “Despite our increased awareness of medical error, studies show that our overall rates of events have not significantly decreased in recent years. The Institute for Healthcare Improvement (IHI) developed the Global Trigger Tool (GTT) to assist in event detection. This tool estimates event rates based on triggers, such as naloxone administration and medication stop orders.
- Using the Global Trigger Tool, it is estimated that one in every three inpatient admissions includes an adverse event. Of these events, it is estimated that 4-15% may cause severe harm.
- Most hospital systems currently do not have sufficient automated detection tools to determine true event rates and must rely on voluntary event reporting. Unfortunately, voluntary event reporting may detect as little as one percent of adverse events.”

**Didactic Slide #9**

- “How do we report? Most hospitals have an online reporting system, which is accessed on the hospital home page. Event reports are often reviewed by the patient safety committee, assigned physician reviewers, and are scored based on severity. Based on the severity score, adverse events may be presented for a root cause analysis, reviewed for actionable recommendations, or presented before a peer review committee. Action items based on systems lapses are then reported to the hospital leadership for review and assigned to a responsible party.

**Didactic and Discussion Slide #10**

- “How are we protected when reporting? You are protected under US code 5705 by penalty of fine for any unauthorized disclosure of the event outside of the review process. If a need for peer review is discovered during proceedings such as an RCA, the RCA process is stopped until the peer review or legal process is completed. When reporting, you can remove your name and contact information in order to remain anonymous in most institutions, especially if you are concerned about retaliation or repercussion. If you do include your name, you, or those involved, may be contacted for more information by the patient safety officers, however this information remains confidential.
- Discussion Questions:
  - Have you ever refrained from reporting an error due to the fear of repercussion or a lack of anonymity?
  - Did you know that you can report anonymously if you are concerned?
  - Were you aware that you are legally protected when reporting an event? *(Pause for discussion).*

**Didactic Slide #11**

- “What good has come from reporting? Examples of processes that have resulted from patient safety events and reporting include the following:
  - Medication order sets, for medications like narcotics with hold precautions
  - Surgical and procedural timeouts
  - Pharmacy review of medication administration and schedules
  - Individual bar code scanning for patient medications
  - Fall precautions for patient protection
  - Imaging for retained foreign objects
  - And order set bundles such as a sepsis protocol”
- Optional discussion questions:
  - Were you aware that these processes have resulted from patient safety events?
  - Have you been involved in a patient safety event that resulted in a change in policy?
  - Did you receive feedback after reporting that changes were made to policy as a result?

**Discussion Slide #12**

- “In your experience, what do you think makes a good event report? (*Allow time for the group to discuss their ideas for a good event report)*
- A good report includes the following:
  - A thorough, concise description of the event: Please provide patient identifiers in the appropriately designated boxes. Please ensure HIPAA compliance when completing.
  - A brief discussion of potential contributing factors to the event
  - The lapse of care that was not provided to patient during the event
  - And potential steps to prevent recurrence, or action items”

**Discussion Slide #13**

- “What would you want to avoid in reporting, or what information would not be helpful for the patient safety team? (*Pause for discussion)*
- Reporting the following is not helpful or useful:
  - Interpersonal conflict: Do not report interpersonal concerns, such as disagreements with nursing staff or pharmacy, unless these have had an effect on patient care or safety. If you have concerns regarding a colleague’s ability to perform their job function, there are avenues to report at your institution, including discussing with your supervisor.
  - Stigmatizing language: Avoid words such as fault, guilt, criticism, incompetence, and punishment, as these lead to blaming individuals. Please refrain from emotional or inflammatory language.
  - Statements of blame: Please help to prevent accusing the individual for not completing an action, rather than the system that put the individual at risk for not completing the action.
  - Names of individual staff: Please include roles and shifts for the patient safety committee to identify and discuss with all involved, but do not include individual names.

**Didactic Slide #14**

- “For the rest of this session, we will focus on teaching the I-SAFEST model for event reporting. I-SAFEST is a rubric for quality content included in event reports. It was developed with a multidisciplinary team, including patient safety officers, residents, and nurses.
- The model includes: the patient’s information, the staff involved in the event, a brief description of the actual event, the follow-up initiated by the primary team, the effect on the patient, the standard of care that was not met, and any potential to-do’s or action items that you would recommend for future prevention. We will now review these components in detail using an interactive case-based discussion.”
- *(Allow 20 mins for the following case discussion section, slides 15-35)*

**Discussion Slide #15**

- “Let’s practice by reading this case.”
- (Resident or facilitator)- “Mr. Roberto Gonzalez (MRN 03478561) arrived to the Emergency Room at 9:00AM on 8/10 with a fever and a painful, swollen right knee. At 1:30PM the EM physician evaluated him, consulted Orthopedics for arthrocentesis, and ordered intravenous (IV) antibiotics. The Orthopedic senior resident, Denise Nguyen, asked her second year resident, Luke Brown, to aspirate the knee for fluid culture prior to antibiotics. Orders were placed to admit the patient to the floor (Unit 5C), and the patient arrived at 6:00PM in a good deal of pain.
- After seeing two emergent consults, Dr. Brown arrived at 8:30PM to see the patient on the floor, who was frustrated about his pain. Dr. Brown was unable to obtain fluid during the arthrocentesis. He called to notify Dr. Nguyen and the attending Dr. George Castle, but they were both in the OR with an emergent case. The team decided to start antibiotics prior to arthrocentesis for continued fever and mild hypotension. One antibiotic was on short supply from pharmacy, and at 11:30PM they were both started. Dr. Nguyen performed arthrocentesis successfully at 12:30AM.
- The fluid culture returned positive and the patient was treated appropriately with narrowed coverage. Mr. Gonzalez suffered more pain and discomfort than would have been expected with timely care, but he sustained no lasting effects. The primary team apologized to the patient and informed him that they would review their staff availability and policies for antibiotic administration.”
- (Facilitator)- “Is the case clear, or do you have any questions?”
  - What type of error does this case fall into? Was this an adverse event or a near miss?
  - Do you think this type of error was preventable? *(Pause for discussion).*
- Let’s review the case in more detail.

**Didactic Slide #16**

- “The first section is information. This refers to the patient’s information, such as identifiers, location, and time of the event. Most electronic forms have a blank for this protected patient information. Make sure to use the appropriate blanks, as some forms require the patient information be removed from the description of the event itself.”

**Discussion Slide #17**

- “Please state the important identifying information about the event. Make sure to think about which pieces of information might be most important to identify this particular case.”
- Residents answer question while viewing case.

**Discussion Slide #18**

- “Very good, the patient’s information in this case is highlighted in red. Mr. Roberto Gonzalez, MRN 03478561. The event occurred in the emergency room at 9:00AM on 8/10.”
  - If residents miss one of the four major points (name, MRN, location, and time), make sure to point out the importance. For example:
    - “I see that you included all of the information except for the time. It may be difficult for reviewers to identify the error without the time and date, such as an incorrect insulin order or medication administration.”
    - “Do you see how you may need two identifiers for each patient? There may be two patients named Mr. Gonzalez in the ED at any given time and another piece of information will confirm.”

**Didactic Slide #19**

- “Next we will discuss the staff involved. This section describes the roles and shifts of all individual staff involved in order to initiate follow-up review of the event.
- Make sure to avoid individual names of staff for protection and safety.”

**Discussion Slide #20**

- “Please identify the staff involved. Remember to include positions but not individual staff names.”
- Residents answer question while viewing case.

**Discussion Slide #21**

- “The staff involved included the emergency room physician, the orthopedic senior resident, and second year resident, as well as the orthopedic attending physician, highlighted in red.”
  - If residents answer incorrectly by providing names, make sure to emphasize the protection of these individuals. For example:
    - “I see that you included the second year resident’s name, Luke Brown. Patient safety teams seek to identify the position in order to understand the event, but not to punish any individual involved. If the team needs to review the case and speak with the individual, they will ask and identify the team member needed.”

**Didactic Slide #22**

- “Next we will discuss the actual event description. The goal of this portion is to briefly describe the event so that the patient safety team may understand what happened.
- Be sure to include key causative factors or contributors to the event.”

**Discussion Slide #23**

- “How would you describe the actual event in the event report.”
- Residents answer question while viewing case.

**Discussion Slide #24**

- “A brief description would include that the patient presented with fever and a painful knee, orthopedics was consulted and ordered antibiotics, however there were several emergent consults that occupied staff. After an unsuccessful procedure, both diagnostics and treatment were significantly delayed but eventually administered.”
  - If residents left out a crucial part, emphasize its importance. For example:
    - “I see that you have mentioned a delay in antibiotics but not any potential causative factors. It would be very helpful for patient safety teams to have the frontline provider’s insight into what other obstacles (i.e. emergent consults, understaffing) may have contributed to the delay.”

**Discussion Slide #25**

- “Next we will discuss the follow-up initiated. This section describes the actions taken by the primary team to inform the patient or other professionals regarding the event.
- This step is crucial to both the patient’s and physician’s confidence and an attempt to remedy or improve upon the error.”

**Discussion Slide #26**

- “Please describe the follow-up initiated by the primary team.”
- Residents answer question while viewing case.

**Discussion Slide #27**

- “The follow-up included the team’s apology to the patient and review of staff policies for antibiotic administration. If the patient had not yet been made aware, make note of this in the event report.”
  - If residents included another item such as the effect on the patient, clarify this item.
  - Optional discussion question: Have you or your team members had to disclose an error or make an apology to a patient? How was the experience? Did this step help your relationship with your patient, or was there difficulty in the process?

**Didactic Slide #28**

- “Next we will discuss the effect on the patient. This effect may be clinical, psychological, or a near-miss that did not reach the patient.”

**Discussion Slide #29**

- “Please identify the effect on the patient.”
- Residents answer question while viewing case.

**Discussion Slide #30**

- “The effect included that the patient suffered more pain and discomfort than would have been expected with timely care, but sustained no lasting effects. If the event was caught prior to reaching the patient, consider calling it a near miss.”
  - Optional discussion question: Have you encountered a negative effect on a patient due to a medical error? How did this event make you feel?

**Didactic Slide #31**

- “Next we will discuss the standard of care. This includes the appropriate course of action that was or was not taken during the event.
- For example, if a patient who underwent surgery six hours ago is required to have a post-operative check by a physician and this does not occur, that would be the standard of care that was missed during the event.”

**Discussion Slide #32**

- “Please describe the standard of care that was or was not provided to the patient.”
- Residents answer question while viewing case.
- “The standard of care that was not met was the timely administration of diagnostic testing and treatment for septic arthritis.”
  - This topic may require further explanation or examples, such as: “Consider guidelines-based care, such as obtaining a rapid troponin and EKG in the ED for a patient with chest pain, or repeating a lactate at 12 hours for a patient with sepsis. These would be the standard that physicians who practice evidence-based care would follow.”

**Didactic Slide #33**

- “Next we will discuss the last section, to-do’s or action items. This includes possible steps for systems improvement or action items considered by the reporter.”

**Discussion Slide #34**

- “Please identify potential action items that may prevent this error from happening again.”
- Residents answer question while viewing case.
- “Action items may include revision to the staff supervision policy or training for residents regarding arthrocentesis protocols.”
  - Residents may answer differently as there are several possible action items.
  - “Can you think of any other action items that may help prevent cases like this from happening in the future?” *(Pause for discussion).*

**Discussion Slide #35**

- “How did we do? A quick event report may appear like the following:
  - Mr. Roberto Gonzalez, MRN 03478561, Unit 5C
  - Patient was admitted on August 10^th^ for septic arthritis. He was initially evaluated at 1:30PM in the ED, however there was a delay in diagnostic arthrocentesis and administration of antibiotics due to a lack of available staff and delay of antibiotic delivery. He was evaluated by the orthopedic senior and junior resident, but they were unable to see the patient until 8:30PM because of other urgent procedures. Patient received antibiotics at 11:30PM from pharmacy, and underwent successful arthrocentesis at 12:30AM.
  - The patient suffered more pain and a risk of sepsis due to this delay, but recovered without long-term injury. The standard of care would be to receive timely arthrocentesis and antibiotics. The Orthopedic staff discussed with the patient’s family and planned to review their policies for antibiotic administration.
  - Would recommend reviewing the staff supervision policy and training for residents on arthrocentesis.”
- Does this framework for reporting errors make sense to you?
- Is there anything you would include or exclude from this framework for your future reporting? *(Pause for discussion).*

**Conclusion Slide #36**

- “The key take home points to remember are that event reporting helps to identify areas of risk for patient safety in the hospital, remember to provide clear information without interpersonal conflicts, and that your efforts can be very useful for promoting change.”

**Conclusion Slide #37**

- “These are the references for the topics discussed today.”

**Conclusion Slide #38**

- Pass out the post-training survey (Appendix B).
- “Please take 10 minutes to complete the post-course survey. Read the fictional patient case and apply what you have learned by writing a brief report of this event using the information you would deem necessary for the patient safety team at your hospital to review.”
- *(Allow 10 minutes for survey completion)*

**Conclusion Slide #39**

- “Are there any questions or comments on the patient safety event report you just completed?” *(Pause for questions or discussion).* “Thank you for your participation.” Dismiss the training conference.
