# Supplementary material for: Use of a Rubric to Improve the Quality of Internal Medicine Resident Event Reporting
Source: MedEdPORTAL. 2021 Oct 11;17:11189. doi: 10.15766/mep_2374-8265.11189 (PMC8502786; doi:10.15766/mep_2374-8265.11189)
Supplement: Supplementary file 1 — Pretraining Survey.docxPosttraining Survey.docxResident Training Module.pptxInstructor Guide.docxResident Training Module Script.docxI-SAFEST Scoring Sheet.docx [file mep_2374-8265.11189-s001.zip › F. I-SAFEST Scoring Sheet.docx]

| Name: | - Pre-Course Report - Post-Course Report |
| --- | --- |
| Date: |  |

Instructions: Each item receives one point if the response adequately addresses the item listed, and zero points if it does not. Total possible score is 10 points for each report.

| **Category** | **Scoring Instructions** | **Score** |
| --- | --- | --- |
| **Information** | 0 points: No descriptor from either category  1 point: One descriptor from either category:  A) Patient name or MRN, OR B) Location or date of event  2 points: One descriptor from both categories:  A) Patient name or MRN, AND 2) location or date of event |  |
| **Staff Involved** | 0 points: Roles of no staff members involved  1 point: Role of at least one staff member  2 points: Roles of at least three staff members |  |
| **Actual Event Description** | 0 points: No description  1 point: Brief description of event without key causative factors (i.e. medication given to incorrect patient)  2 points: Description of event with at least one immediate or remote causative factor (i.e. medication given to incorrect patient due to verification error or shift change) |  |
| **Follow-up Initiated** | 0 points: No description  1 point: Description of follow-up or reparations initiated by team (i.e. discussed with family, reviewed with nursing staff, etc.) |  |
| **Effect on the Patient** | 0 points: No description  1 point: Description of effect or harm to patient (may be immediate or long term), or near-miss |  |
| **Standard of Care** | 0 points: No description  1 point: Description of best practice that was not met |  |
| **To-Do** | 0 points: No description  1 point: Notation of at least one potential action item or area needing review |  |
| **Total (0-10)** | |  |
