# Supplementary material for: Use of a Rubric to Improve the Quality of Internal Medicine Resident Event Reporting
Source: MedEdPORTAL. 2021 Oct 11;17:11189. doi: 10.15766/mep_2374-8265.11189 (PMC8502786; doi:10.15766/mep_2374-8265.11189)
Supplement: Supplementary file 1 — Pretraining Survey.docxPosttraining Survey.docxResident Training Module.pptxInstructor Guide.docxResident Training Module Script.docxI-SAFEST Scoring Sheet.docx [file mep_2374-8265.11189-s001.zip › A. Pretraining Survey.docx]

**Error Reporting Pre-Survey**

**PGY Level ___________ Last 4 digits of phone number ________________**

**(For matching purposes only)**

| **1. During your last inpatient rotation, how many errors per month did you notice?**  **Please include close calls and near misses as well.**  Hospital Pavilion: ____________________________ Number of errors: _______________ | | | | | |
| --- | --- | --- | --- | --- | --- |
| **2. How many of these errors did you report?** Number of reports: ______________ | | | | | |
| **3. How important is event reporting to your practice?** | | | | | |
| Not at all important | | | Not very important | Somewhat important | Extremely Important |
| **4. How knowledgeable are you about the content that should be included in an effective event report?** | | | | | |
| Not at all knowledgeable | | | Not very knowledgeable | Somewhat knowledgeable | Extremely knowledgeable |
| **5. How confident are you about your skills in writing an effective event report?** | | | | | |
| Not at all confident | | | Not very confident | Somewhat confident | Extremely confident |
| **6. Check all of the following barriers that you have encountered while reporting an event.** | | | | | |
| Yes | No |  | | | |
| ⃝ | ⃝ | Time required | | | |
| ⃝ | ⃝ | Inadequate knowledge of what to report | | | |
| ⃝ | ⃝ | Lack of feedback | | | |
| ⃝ | ⃝ | Fear of retaliation | | | |
| ⃝ | ⃝ | Difficulty of using interface | | | |
| ⃝ | ⃝ | Other: | | | |

Please read the following case and write an event report in the blank below, including all relevant and important data points that you would deem necessary for the Patient Safety Department to review.

**Patient Case**

Mr. Stephen Davis (MRN 80054783) is an 89 year-old man who was admitted to the hospital two days ago for a hip fracture. He underwent an open reduction internal fixation (ORIF) without complication and was transferred to the medicine floor on unit 7A. On postoperative day 1, the patient was weaned off of a morphine drip, but his pain was difficult to control with PRN hydrocodone-acetaminophen. After failing several pain medications, the primary team decided to schedule his hydrocodone. The intern, Jason Smith, wrote for hydrocodone scheduled every six hours.

On postoperative day 3 (June 5), the patient returned in the afternoon from an ultrasound in severe pain, as he had not received his morning dose of hydrocodone. The nurse covering that afternoon, Sarah Evans, was not familiar with the patient. She gave his AM dose several hours late at 12PM, and then again at 2PM on schedule, though he looked a little sleepy. The pharmacist, Andrew Shaw, verified that the order was scheduled and approved it.

A rapid response was called at 3PM for inability to rouse the patient. He was given two doses of naloxone, placed on a drip, and admitted to the MICU for treatment. Mr. Davis improved clinically and did not suffer long term harm, however the family was upset. The team met with the patient and his family, apologized for the error, and reported that they would discuss with pharmacy and nursing to prevent this from happening again.
