# Supplementary material for: Use of a Rubric to Improve the Quality of Internal Medicine Resident Event Reporting
Source: MedEdPORTAL. 2021 Oct 11;17:11189. doi: 10.15766/mep_2374-8265.11189 (PMC8502786; doi:10.15766/mep_2374-8265.11189)
Supplement: Supplementary file 1 — Pretraining Survey.docxPosttraining Survey.docxResident Training Module.pptxInstructor Guide.docxResident Training Module Script.docxI-SAFEST Scoring Sheet.docx [file mep_2374-8265.11189-s001.zip › B. Posttraining Survey.docx]

**Error Reporting Post-Survey**

**PGY Level ___________ Last 4 digits of phone number ________________**

**(For matching purposes only)**

| **1. How enjoyable did you find this teaching module for event reporting?** | | | |
| --- | --- | --- | --- |
| Not at all enjoyable | Not very enjoyable | Somewhat enjoyable | Extremely enjoyable |
| **2. How important is event reporting to your practice?** | | | |
| Not at all important | Not very important | Somewhat important | Extremely important |
| **3.** **How knowledgeable are you about the content that should be included in an effective event report?** | | | |
| Not at all knowledgeable | Not very knowledgeable | Somewhat knowledgeable | Extremely knowledgeable |
| **4.** **How confident are you about your skills in writing an effective event report?** | | | |
| Not at all confident | Not very confident | Somewhat confident | Extremely confident |
| **5. How likely are you to apply the I-SAFEST model to event reporting in your practice?** | | | |
| Not at all likely | Not very likely | Somewhat likely | Extremely likely |

Please read the following case and write an error report in the blank below, including all relevant and important data points that you would deem necessary for the Patient Safety Department to review.

**Patient Case**

Mrs. Kristen Jones (MRN 27002145) is a 70 year-old female who was admitted to general medicine floor unit 8B two days ago with community-acquired pneumonia. During her second hospital night, the lab technician, John Cheng, called the medicine floor with a critical lab value at 10PM. The nurse who was caring for the patient was out on break, and the covering nurse, Tim Adams, took the message. Mrs. Jones’s glucose was elevated to 456. Mr. Adams confirmed the value and called the medicine hospitalist, Dr. Maureen Bowers, with the results. Dr. Bowers was seeing another patient at the time, but she logged into the electronic medical record (EMR) and ordered 12 units of regular insulin. She asked the nurse to give the medication, which he did promptly, and said that she would see the patient shortly.

On assessment at 11PM, Mrs. Jones was lethargic. A finger stick was performed with her glucose level at 30 mg/dL. D50 was quickly given, and Dr. Bowers checked the EMR. She found that there was another patient on unit 8B with the same last name who was an uncontrolled diabetic, and for whom the panic value was called.

Mrs. Jones improved quickly with treatment, and the other patient was treated appropriately. The patient was informed of the mistake, and Dr. Bowers discussed her concern regarding the mistake with the charge nurse on 8B and the lab administrator.
