# Supplementary material for: Use of a Rubric to Improve the Quality of Internal Medicine Resident Event Reporting
Source: MedEdPORTAL. 2021 Oct 11;17:11189. doi: 10.15766/mep_2374-8265.11189 (PMC8502786; doi:10.15766/mep_2374-8265.11189)
Supplement: Supplementary file 1 — Pretraining Survey.docxPosttraining Survey.docxResident Training Module.pptxInstructor Guide.docxResident Training Module Script.docxI-SAFEST Scoring Sheet.docx [file mep_2374-8265.11189-s001.zip › D. Instructor Guide.docx]

**Appendix D: Instructor Guide**

**Instructors:**

One chief resident or faculty facilitator

*Facilitator –* The facilitator should ideally be a faculty member or chief resident with basic quality improvement and patient safety (QI/PS) training. At our institution, the training workshop is led by the Chief Residents in Quality and Safety (CRQS). In general, we have found it advantageous to have personnel who have reviewed event reports at their institution or participated on patient safety teams. If there is a lack of personnel with this type of PS and QI training, the basic background knowledge for teaching this session can be obtained through resources such as the Institute for Healthcare Improvement (IHI) or VA National Center for Patient Safety (NCPS). During the session, facilitators help review pertinent details regarding the process of reporting and guide residents through a patient case, providing feedback on their assessment of a safety event. These sessions were held in a small-group format to promote interactive discussion between residents and facilitator; thus the role of chief resident or CRQS was particularly useful for making residents feel more comfortable with sharing.

**Learners:**

All internal medicine and combined Medicine-Pediatrics residents during protected educational time; however this can be easily adapted to any learner group (e.g. students, fellows)

**Setting:**

Small-group sessions for facilitated discussion. At our institution, we held eight separate sessions for 16-18 trainees each. We found that a round-table set up was most beneficial for facilitating group discussion among the residents. The limited number of trainees promoted psychological safety and community that allowed residents to feel comfortable telling their own stories of de-identified patient experiences, commenting on their colleagues’ experiences, and answering questions about the example cases.

If a small-group or roundtable setting is not available, an alternative to consider would be splitting the residents into groups of 6-8 at the beginning of the module and asking them to discuss patient safety events that they or others have encountered in order to bring them forward to the group. In this setting, it would be useful to have more than one facilitator so that the individual groups may ask questions more easily and ensure ongoing dialogue.

**Teaching methods:**

Case-based didactics with interactive roundtable discussion and practical application

**Media requirements:**

Computer with PowerPoint capabilities, projector. Learners can participate in survey via distribution of paper format, or via laptop or smartphone entry.

**Time allotment:**

60 minutes for workshop

**Required pre-work:**

For the conference, facilitators will typically spend approximately 30 minutes reviewing the PowerPoint presentation. It is helpful to consider examples of patient safety events from the facilitator’s own experiences to provide insight during the session. There is no other pre-work required.

**Session Content:**

See Appendix E for a sample script that can be used to present the slides provided in the PowerPoint file entitled “Resident Training Module” (Appendix C). The slides and script can be modified to meet the needs and preferences of other institutions. We invite the training attendees to participate in discussion throughout the session. See the pre- and post-training surveys available in Appendices A and B, respectively.

**Curriculum Implementation:**

*Resource Requirements* – This section describes the resources allotted for this curriculum at our institution. This can be easily adapted based on resources available at a particular institution.

- Resident time- One hour of time is required for the training session in total. At our institution, each resident has protected time for education every eighth week. To train all residents in the program, we provide this workshop once a week over the span of eight weeks to small groups of residents during their protected educational time. The one-hour training can also be implemented during a noon conference format, morning report, or a standard onboarding for residents.
- Material costs- Costs include provision of a computer with projector. These items are available at our institution without additional cost. Other costs include printing copies of the surveys. However the survey may also be implemented via an online platform and residents may bring a laptop or smartphone to complete the surveys.
- Space- Requirements include a room with a computer and projector set up, and with tables and chairs to hold up to 20 people. We arranged the tables and chairs into a circle to facilitate discussion amount residents.

*Scoring and Feedback* – The I-SAFEST Scoring Sheet (Appendix F) provides the detailed instructions for grading both pre- and post-training event reports. Reports can be matched to the same participant by asking residents to fill out the last four digits of their cell phone number on both the pre- and post-course surveys. At our institution, we used the matched pre- and post-training event report scores to reflect on the baseline information that residents report and how they improved. These scores may be used for individual feedback on patient safety reporting. They may also be implemented to demonstrate active training in patient safety initiatives for the Accreditation Council for Graduate Medical Education (ACGME) Clinical Learning Environment Review requirements.

**Alternatives:**

This module could alternatively and very easily be used to accommodate for physical distancing and/or remote learning. For physical distancing, the PowerPoint format can be used by individuals on separate laptops and the survey can be sent by email to personal devices. For remote learning, this digital format can similarly be used at home with online communication programs such as Zoom® or Microsoft® Teams. Remote learning would allow for the same quick collection of survey results via online submission.

If a projector is not available, the case-based review can be read aloud and discussed orally with the residents. If paper handouts are not available, the survey can be easily reformatted to any electronic survey program, such as SurveyMonkey®, and distributed online by email to participants.
