# Supplementary material for: Use of a Rubric to Improve the Quality of Internal Medicine Resident Event Reporting
Source: MedEdPORTAL. 2021 Oct 11;17:11189. doi: 10.15766/mep_2374-8265.11189 (PMC8502786; doi:10.15766/mep_2374-8265.11189)
Supplement: Supplementary file 1 — Pretraining Survey.docxPosttraining Survey.docxResident Training Module.pptxInstructor Guide.docxResident Training Module Script.docxI-SAFEST Scoring Sheet.docx [file mep_2374-8265.11189-s001.zip › Educational_Summary_Report_11189.pdf]

MedEdPORTAL® | The AAMC Journal of Teaching and Learning Resources

Original Publication Open Access

Use of a Rubric to Improve the Quality of Internal Medicine Resident Event Reporting

Monica Arnell, MD*, Rosemary Demet, MPH, Lindsay Vaclavik, MD, Xiaofan Huang, MS, Kristen A. Staggers, MS, Cecilia Y. Cai, MD, Molly J. Horstman, MD, MS

*Corresponding author: mjarnell@gmail.com

Abstract

Introduction: As frontline providers, residents report patient safety events and provide crucial safety feedback. Specific ACGME and AAMC requirements for graduating residents include active participation in event reporting and patient safety investigations. However, formal training on what information a quality event report should include to effect real change in the health care system is lacking. **Methods:** This practical, interactive, case-based workshop educates residents on the key components of a quality event report in a 1-hour time frame. The scoring rubric offers quantitative feedback on the quality of information provided in residents' own event reports. The materials include a presentation template, sample teaching points, pre- and posttraining patient safety cases for residents to complete their own event reports about, and a standardized rubric to score event reports for feedback. **Results:** During the fall of 2019, 198 internal medicine residents completed the workshop, and 143 matched pre- and postcourse surveys were reviewed. Residents' ability to correctly identify the key concepts of an event report improved from a median score of 4 to 8 ($p < .001$). After completion of training, residents reported increased knowledge regarding the content of an effective event report ($p < .001$) and increased confidence in their ability to write one ($p < .001$). **Discussion:** Residents' knowledge of key event-reporting concepts and confidence in reporting improved after completion of the workshop. This brief interactive training and its novel rubric can be used as a standardized tool for patient safety curricula in academic training programs.

Keywords
Quality Improvement/Patient Safety, Event Reporting, Error Report, Case-Based Learning, Internal Medicine

Educational Objectives

By the end of this activity, learners will be able to:

1. Describe the process and value of event reporting.
2. Write an effective event report using the I-SAFEST model.
3. Improve their confidence in their own reporting skills.

Introduction

Patient safety and quality improvement training are critical aspects of resident education. The Accreditation Council for Graduate Medical Education (ACGME) highlights this in its updated Clinical Learning Environment Review (CLER) pathways to excellence.[1] These pathways state that residents must demonstrate the ability to report safety events at their institutions and that continued patient safety education is essential to a culture of safety.[1] The American Association of Medical Colleges likewise accentuates resident event reporting in its Quality Improvement and Patient Safety (QIPS) competencies.[2] Furthermore, the Joint Commission emphasizes that a learning organization must document every patient safety event to promote a culture of safety and improve patient outcomes.[3]

Citation:
Arnell M, Demet R, Vaclavik L, et al. Use of a rubric to improve the quality of internal medicine resident event reporting. *MedEdPORTAL*. 2021;17:11189. https://doi.org/10.15766/mep_2374-8265.11189

Many barriers inhibit adequate reporting, including a lack of knowledge regarding what information to include and how to submit incident reports.[4,5] Even faculty and resident physicians who endorse the importance of event reporting often lack this knowledge.[6] Confusion about what to report is linked to the absence of standardized event-reporting training during medical education.[7] To date, the event-reporting literature has focused primarily on interventions to improve the quantity of event reporting.[8-10] Aaron, Webb, and Luhanga's 2020 narrative review found that both modeling appropriate reporting behavior and reinforcing the importance of reporting through targeted messaging sustainably increased reporting rates.[11] While these

Copyright © 2021 Arnell et al. This is an open-access publication distributed under the terms of the Creative Commons Attribution license.

interventions have been associated with an increase in the number of reports submitted, few studies have utilized such strategies to improve the quality of information included in event reports. Patient safety officers commonly receive event reports with incomplete information, leading to increased workload for patient safety teams and delays in responding to patient safety events.[8] Furthermore, event reports that include inappropriate information, such as interpersonal conflicts or professionalism concerns, take time away from the investigation of sentinel events that lead to patient harm.[12] There are few guidelines that discuss the quality of the content included in event reports, limiting the efficacy and impact of event reporting.[1,13]

As frontline providers, residents frequently witness patient safety events, reports of which require complete and accurate information to be useful for patient safety officers and to effect change. Reviewing *MedEdPORTAL* publications, we found several modules addressing the mechanics of submitting an event report.[14-18] However, no publications outlined a curriculum aimed at improving the quality of event reports. In 2017, Keefer, Helms, Warrier, Vredeveld, Burrows, and Orringer developed a model for patient safety education in the field of pediatrics.[9] With the help of an interprofessional team and patient safety officers, we adapted Keefer and colleagues' model to create a 10-point I-SAFEST rubric to evaluate the quality of event reporting. The rubric detailed what should be included in a high-quality event report: patient information (I), staff involved (S), actual event description (A), follow-up initiated (F), effect on the patient (E), standard of care (S), and action items for prevention or to-dos (T). We incorporated this rubric into an interactive module describing the exact information that should be included in an event report. Residents were asked to apply their skills by completing an event report for fictional patient safety events both before and after module delivery. The rubric allowed for quantitative scoring of event reports to standardize resident assessment and feedback.

This resource includes an interactive module for effective event reporting and a pre- and posttraining survey with sample case scenarios for residents to complete their own event reports about. The resource also contains the I-SAFEST scoring rubric used to evaluate event-report quality and a comprehensive guide for facilitators.

Methods

We designed this module to communicate the key components of a quality event report to residents through interactive formal training and practice cases. Our primary aim was to increase knowledge regarding what components of an event should be reported. We also aimed to increase residents' sense of self-efficacy in completing event reports.

Training Setting

We created this training for internal medicine and combined medicine-pediatrics residents at Baylor College of Medicine, postgraduate year (PGY) 1-4. We implemented the module in the fall of 2019 and delivered it during protected academic time, when residents had no clinical responsibilities and participated in learning activities on clinical, ethical, and procedural concepts. We delivered the material as a 1-hour interactive session with one facilitator over the course of 8 weeks to groups of 15-20 residents. We found that limiting our training group to no more than 20 learners allowed for meaningful discussion between all participants.

A chief resident in quality and patient safety served as the facilitator for each session. The chief residents in quality and patient safety received comprehensive training in QIPS concepts. Learners were not required to complete any modules prior to the training. We provided the training in a roundtable conference room setting via a PowerPoint presentation and facilitated discussion. We delivered pre- and postcourse surveys using SurveyMonkey and advised learners to bring a computer or cell phone to the session to complete the surveys (Appendices A and B). Although residents were not asked to provide their name on the surveys, we did ask that they include the last four digits of their phone number to link pre- and posttraining surveys. Using published guidelines for academic survey creation,[19] two of the authors created the pre- and postcourse surveys de novo. We conducted a literature review to assess common barriers to reporting cited in the literature and provided these as options for residents to report.[20] The surveys went through two rounds of revisions by coauthors with expertise in patient safety for validation, and pilot testing was completed by second-year medical students taking a patient safety course and by chief residents.

Training Overview

The training consisted of a precourse survey and practice event report, a 40-minute interactive case-based discussion on event reporting, and a postcourse survey and practice event report. Patient safety leaders, including faculty, residents, nurses, patient safety managers, and medical students, collaborated to develop the module. We reviewed the current literature and hospital guidelines delineating the key elements of an event report. Two of the authors created three fictional teaching cases

Copyright © 2021 Arnell et al. This is an open-access publication distributed under the terms of the Creative Commons Attribution license.

inspired by real reported events reviewed on the patient safety committee as well as personal experience. We anonymized the event details and created three common adverse event themes: medication error, treatment delay, and patient misidentification. These cases were independently reviewed by coauthors with expertise in patient safety and were felt to have face validity for commonly reported events. The cases went through two rounds of revision. Pilot testing was then performed on two of the coauthors, second-year medical students, and chief residents, who provided comments and feedback on the cases.

Training Layout

Precourse survey and event report (10 minutes): Residents completed a precourse survey to collect information on their experience and prior exposure to patient safety events, reporting habits, and barriers to reporting. Additionally, they ranked their knowledge and confidence regarding event reporting on a 4-point Likert scale. Residents were asked to write an event report for a fictitious patient safety event of a narcotic overdose (Appendix A). Residents submitted their event report as free text.

Interactive case-based didactics (40 minutes):

1. Open-ended questions (10 minutes): We expressly stated at the beginning of the session that the information provided was confidential and nonpunitive and was being presented in order to inform residents about safety events. To begin the session, we asked residents to share their experiences regarding patient safety and event reporting (Appendix C, slides 1-4). We found this format of starting with open-ended questions to be engaging and interactive. We have provided instructions for presenters and a training module script (Appendices D and E) in the current publication.
2. Event-reporting education and discussion (10 minutes): The didactic presentation detailed key patient safety terms and concepts, including defining medical error, near misses, and preventable events, and reviewed patient safety event rates from the literature. We explained the life cycle of an event report from initial submission to committee review, such as a root cause analysis or peer review, and resulting action items for hospital systems and providers. Additionally, we emphasized the value of reporting by frontline providers and legal protection for reporters (Appendix C, slides 5-13).
3. Interactive case-based discussion (20 minutes): We walked through the I-SAFEST framework for the essential components of successful, high-quality reporting. Residents read a fictional case aloud involving a delay of antibiotic administration. Residents then worked together to enumerate the components of the framework relevant to the case with feedback (Appendix C, slides 14-35).

Postcourse survey and event report (10 minutes): Residents completed a postcourse survey with matched questions regarding their knowledge of and confidence in reporting, as well as their likelihood of using the information learned in their future practice. We asked them to apply their knowledge by writing a postcourse event report for a case involving patient misidentification (Appendix B). Residents submitted their event report as free text.

Grading

No formal grades were delivered for participation in this course. Attendance and participation in scheduled sessions during protected academic time were mandatory for residents.

Assessment

We evaluated residents' perceptions of event reporting by survey using a 4-point Likert scale (1 = *not at all,* 4 = *extremely*). This included residents' own assessment of the importance of event reporting before and after training, as well as their self-reported knowledge and confidence regarding their own reporting. These measures aligned with the New World Kirkpatrick model's first level of reaction to a training module and second level of learning from training.[21] Residents were asked if they were likely to use the behaviors learned in the module in their daily practice, reaching the New World Kirkpatrick model's third level of changing self-reported behaviors.[21] We scored the pre- and posttest event reports using a 10-point I-SAFEST rubric and scoring sheet (Appendix F). Although the components of the I-SAFEST framework were introduced during the interactive case-based didactics, residents did not have access to the rubric or the scoring system when they completed the posttraining event report.

Statistical Analysis

Two independent reviewers graded the event reports from the first two modules, and we assessed interrater reliability using the kappa statistic.[22] Kappas were .9100 and .8417 for pre- and postevent reports, respectively. Given the strong level of agreement, the two reviewers independently graded the remaining event reports. We analyzed paired pre- and

Copyright © 2021 Arnell et al. This is an open-access publication distributed under the terms of the Creative Commons Attribution license.

posttraining survey responses and written event-report scores using Wilcoxon signed rank tests. We performed a subgroup analysis by PGY level.

Institutional Review Board Approval

The evaluation was reviewed and approved by the Baylor College of Medicine Institutional Review Board with a waiver of written informed consent.

Results

Participant Characteristics

A total of 198 categorical residents participated in the training exercise. From these participants, 143 matched pre- and posttraining surveys were completed and reviewed. Respondents were composed of 39% PGY 1 ($n = 55$), 30% PGY 2 ($n = 43$), 30% PGY 3 ($n = 43$), and 1% PGY 4 ($n = 2$) residents. Participants encountered an average of 3.4 events per month, ranging from 0 to 30, but they completed an event report for only 21% of the events they encountered.

Quantitative Results

After completion of training, 89% of residents ($n = 127$) found the training either somewhat or extremely enjoyable. Most residents reported that they were either somewhat likely (55%, $n = 79$) or very likely (36%, $n = 52$) to use the knowledge gained from the course in their practice.

Matched pre- and posttraining survey data on resident attitudes and behaviors were measured using a 4-point Likert scale (Table). With training, residents reported increased confidence in their ability to write an effective event report ($p < .001$). They endorsed having improved knowledge regarding the content of an effective event report ($p < .001$). Residents expressed an increase in the importance of event reporting in their own practice after the teaching module ($p = .008$). In a subgroup analysis for each PGY level, there were significant improvements in reported knowledge and confidence after the intervention for PGY levels 1, 2, and 3. The number of PGY 4 residents who participated was not sufficient to draw a conclusion for that group.

Table. Attitudes Toward Event Reporting[a]

	Median (25th, 75th Percentile)		
**Self-Reported Response**	**Pretraining**	**Posttraining**	***p***
Importance of reporting	3 (3, 4)	3 (3, 4)	.008
Knowledge of reporting	3 (2, 3)	3 (3, 4)	<.001
Confidence in reporting	2 (2, 3)	3 (3, 3)	<.001

[a]Self-reported attitudes, including the importance of reporting, resident knowledge, and resident confidence in the practice of reporting, improved significantly.

Residents' ability to correctly identify the key concepts of an event report improved from a median score of 4 to 8 ($p < .001$; see Figure 1). There were significant improvements in total scores after the intervention for PGY levels 1-3. The PGY 4 sample size was not sufficient to make conclusions regarding significance. Individual category scores showed improvement in all areas, including patient information ($p < .001$), staff involved ($p < .001$), and to-do or action items ($p < .001$). The only category that did not show improvement was the effect on the patient ($p = .6$). For individual categories, high-quality reports were defined as a perfect score in that category (Figure 2).

Discussion

While residency programs strive to meet ACGME patient safety standards, the lack of knowledge surrounding what information to report often hinders event-report submissions.[1,5] Our training improved residents' knowledge and confidence regarding reporting. The participation rates and positive feedback indicated that the residents appreciated this systematic training to reduce confusion surrounding how to report adverse events and what to include. Our subgroup analysis showed that PGY levels 1-3 reported improvement, highlighting the importance of such training at all education levels.

Residents act as frontline providers, uniquely exposed to medical errors and positioned to report them.[1,2] Current training programs rarely address what to include in reports, which may impede resident efforts to report. Although a prompted electronic event report may address this issue, residents often rotate through multiple institutions, each with its own reporting system with different reporting forms and structures. Thus, it is important that residents understand what information is needed for a complete event report. This module helps residents navigate the important aspects of reporting and provides training programs with a means to assess the quality of submitted reports. The training includes a posttraining case, inviting residents to apply their newly acquired knowledge to the practice of reporting. Although we have been unable to assess the impact of any behavior change (Kirkpatrick's fourth level), we hope that the standardized training for event reporting will improve overall report quality and patient safety at participating institutions.

Introducing standardized content of event reporting is an important step in meeting ACGME CLER pathway standards of residency training.[1] The length of this module—a single hour—makes it an ideal training to incorporate into a residency program, whether in a noon conference or a designated patient safety

Copyright © 2021 Arnell et al. This is an open-access publication distributed under the terms of the Creative Commons Attribution license.

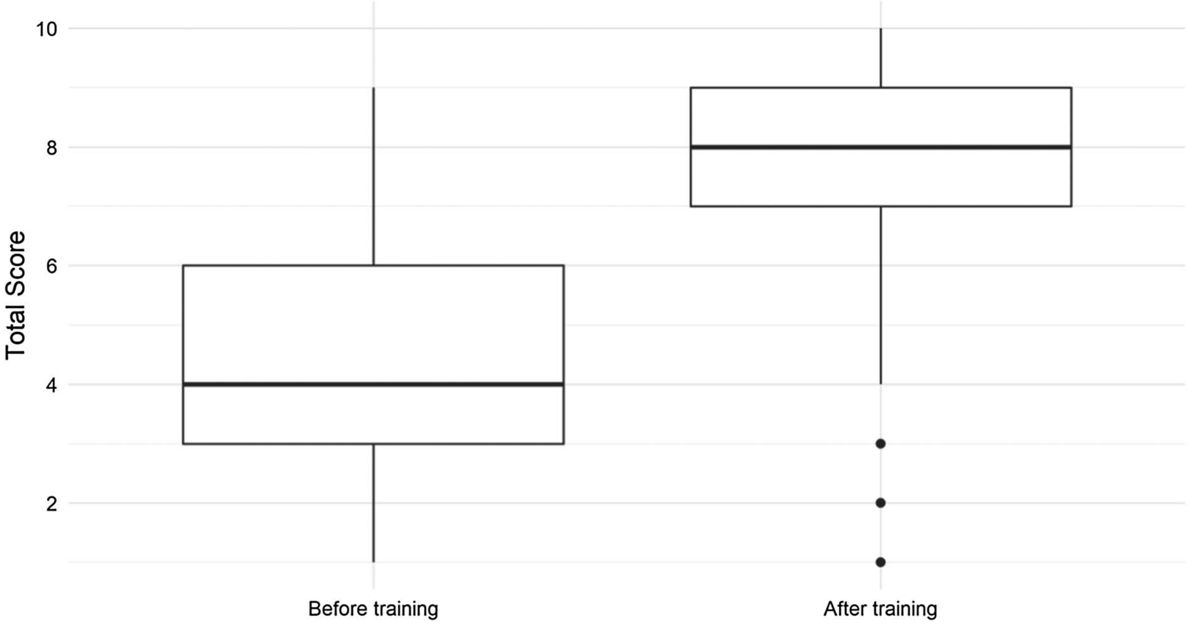

Figure 1. Total score on the I-SAFEST rubric. Scores improved from a median of 4 (interquartile range: 3) to 8 (interquartile range: 2, $p < .001$) for all residents after training. The horizontal bar above the thick black line is the 75th percentile, and the bar below is the 25th percentile. The ends of the vertical lines indicate maximum and minimum. The thick black line in the middle of the boxes indicates the median. The three black dots below the box in the "After training" column indicate outliers.

curriculum. Our cases are designed specifically for internal medicine residents, but the fictional adverse events could easily be altered to align with any area of training and still maintain the integrity of the module.

One of the challenges we encountered was that we were not able to obtain real event-report data submitted by internal medicine residents. Real-world event reports are protected information and are often submitted anonymously, without identifying the name or type of trainee. Therefore, simulated cases were necessary to assess the quality of reporting by residents, as we could not use real event reports to assess their application of learning. We are continuing to work with the patient safety offices at the different teaching hospitals to determine whether we can access these data in the future. The use of hypothetical cases does, however, support the widespread distribution of the module. Another challenge during module development was to create fictional cases that reflected the

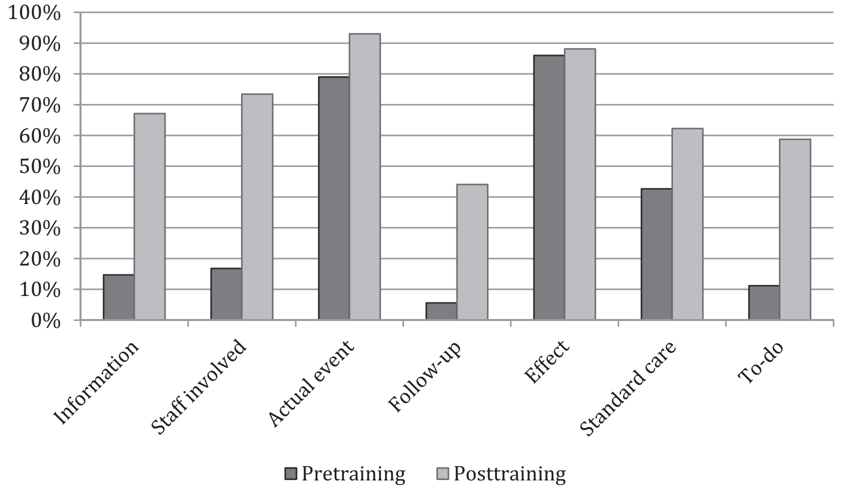

Figure 2. Percentage of high-quality reports before and after training. High-quality reports were defined as a perfect score in that category.

Copyright © 2021 Arnell et al. This is an open-access publication distributed under the terms of the Creative Commons Attribution license.

complexity of real adverse events but also remained succinct as practice cases. Residents gave helpful feedback during pilot testing to ensure the cases were realistic and engaging.

The evaluation of this training was limited by its scope of implementation at a single residency program. Its successful implementation at a large, multihospital academic center, however, has demonstrated its relevance to similar academic institutions. Additionally, we were unable to obtain follow-up data months later to assess retention. Thus, the extent of decay in the residents' ability to write a complete event report is unknown.

Finally, it is important to mention that the number of surveys required during the 1-hour training may introduce survey fatigue, potentially compromising the validity of responses later in the course. While this cannot be entirely eliminated, posttraining surveys allow residents to practice event reporting using a learned model, and our results demonstrate improved knowledge and confidence.

Incorporating this module into protected educational time allowed for high levels of attendance, meaningful participation, and positive feedback. The training can be easily adapted to meet the needs of other institutions and training programs. The novel I-SAFEST assessment has the potential to standardize reporting, streamlining quality improvement efforts and clarifying instruction offered to residents during their patient safety training.

Appendices

A. Pretraining Survey.docx

B. Posttraining Survey.docx

C. Resident Training Module.pptx

D. Instructor Guide.docx

E. Resident Training Module Script.docx

F. I-SAFEST Scoring Sheet.docx

All appendices are peer reviewed as integral parts of the Original Publication.

Monica Arnell, MD: Clinical Instructor, Department of Medicine, Houston Methodist; ORCID: https://orcid.org/0000-0001-8730-3204

Rosemary Demet, MPH: Second-Year Medical Student, Baylor College of Medicine

Lindsay Vaclavik, MD: Assistant Professor of Medicine, Department of Medicine, Baylor College of Medicine

Xiaofan Huang, MS: Biostatistician, Institute for Clinical and Translational Research, Baylor College of Medicine

Kristen A. Staggers, MS: Biostatistician, Institute for Clinical and Translational Research, Baylor College of Medicine

Cecilia Y. Cai, MD: Clinical Fellow, Department of Medicine, Johns Hopkins University School of Medicine

Molly J. Horstman, MD, MS: Assistant Professor of Medicine, Department of Medicine, Baylor College of Medicine; Investigator, Center for Innovations in Quality, Effectiveness, and Safety, Michael E. DeBakey VA Medical Center

Disclosures
None to report.

Funding/Support
This work is supported in part by the Department of Veterans Affairs, Veterans Health Administration, Office of Academic Affiliations VA Quality Scholars Program Coordinating Center Grant and the Office of Research and Development, Houston Health Services Research and Development Center for Innovations in Quality, Effectiveness and Safety (IQuESt) grant (CIN 13-413) at the Michael E. DeBakey VA Medical Center, Houston, Texas. This work was also supported by American Heart Association Grant 17MCPRP33390011 (Horstman).

The authors declare no conflict of interest.

Prior Presentations
Arnell MJ, Pfaff R, Cai C, et al. Patient safety in action: improving the quality of resident event reporting. Presented at: Baylor College of Medicine Academy of Distinguished Educators Annual Showcase of Educational Scholarship; September 18, 2019; Houston, TX.

Arnell MJ, Pfaff R, Cai C, et al. Patient safety in action: improving the quality of resident event reporting. Poster presented online at: Society of Hospital Medicine HM20 Virtual Poster Competition; July 15, 2020.

Ethical Approval
The Baylor College of Medicine Institutional Review Board approved this project.

Disclaimer
The views expressed in this publication are those of the authors and do not necessarily reflect the position or policy of the Department of Veterans Affairs, the United States government, or other affiliated institutions.

References

1. CLER Evaluation Committee. *CLER Pathways to Excellence: Expectations for an Optimal Clinical Learning Environment to Achieve Safe and High-Quality Patient Care, Version 2.0.* Accreditation Council for Graduate Medical Education; 2019. Accessed May 5, 2020. https://acgme.org/Portals/0/PDFs/CLER/1079ACGME-CLER2019PTE-BrochDigital.pdf

Copyright © 2021 Arnell et al. This is an open-access publication distributed under the terms of the Creative Commons Attribution license.

2. AAMC QIPS competencies. Association of American Medical Colleges. February 20, 2019. Accessed May 5, 2020. https://www.aamc.org/system/files/c/2/493150-qipscompetencies.pdf
3. Joint Commission. *2021 Comprehensive Accreditation Manual for Hospitals (CAMH)*. JCR Publishing; 2021:PS-4. Accessed August 9, 2021. https://www.jointcommission.org/-/media/tjc/documents/standards/ps-chapters/camh_04a_ps_all_current.pdf
4. Hatoun J, Suen W, Liu C, et al. Elucidating reasons for resident underutilization of electronic adverse event reporting. *Am J Med Qual*. 2016;31(4):308-314. https://doi.org/10.1177/1062860615574504
5. Schectman JM, Plews-Ogan ML. Physician perception of hospital safety and barriers to incident reporting. *Jt Comm J Qual Patient Saf*. 2006;32(6):337-343. https://doi.org/10.1016/S1553-7250(06)32043-0
6. Kaldjian LC, Jones EW, Wu BJ, Forman-Hoffman VL, Levi BH, Rosenthal GE. Reporting medical errors to improve patient safety: a survey of physicians in teaching hospitals. *Arch Intern Med*. 2008;168(1):40-46. https://doi.org/10.1001/archinternmed.2007.12
7. Logio LS, Ramanujam R. Medical trainees' formal and informal incident reporting across a five-hospital academic medical center. *Jt Comm J Qual Patient Saf*. 2010;36(1):36-42. https://doi.org/10.1016/S1553-7250(10)36007-7
8. Macrae C. The problem with incident reporting. *BMJ Qual Saf*. 2016;25(2):71-75. https://doi.org/10.1136/bmjqs-2015-004732
9. Keefer P, Helms L, Warrier K, Vredeveld J, Burrows H, Orringer K. SAFEST: use of a rubric to teach safety reporting to pediatric house officers. *Pediatr Qual Saf*. 2017;2(6):e045. https://doi.org/10.1097/pq9.0000000000000045
10. Howell AM, Burns EM, Hull L, Mayer E, Sevdalis N, Darzi A. International recommendations for national patient safety incident reporting systems: an expert Delphi consensus-building process. *BMJ Qual Saf*. 2017;26(2):150-163. https://doi.org/10.1136/bmjqs-2015-004456
11. Aaron M, Webb A, Luhanga U. A narrative review of strategies to increase patient safety event reporting by residents. *J Grad Med Educ*. 2020;12(4):415-424. https://doi.org/10.4300/JGME-D-19-00649.1
12. Noble DJ, Pronovost PJ. Underreporting of patient safety incidents reduces health care's ability to quantify and accurately measure harm reduction. *J Patient Saf*. 2010;6(4):247-250. https://doi.org/10.1097/PTS.0b013e3181fd1697
13. Gong Y, Kang H, Wu X, Hua L. Enhancing patient safety event reporting: a systematic review of system design features. *Appl Clin Inform*. 2017;8(3):893-909. https://doi.org/10.4338/ACI-2016-02-R-0023
14. Goolsarran N. Patient safety education curriculum for medicine residents. *MedEdPORTAL*. 2015;11:10208. https://doi.org/10.15766/mep_2374-8265.10208
15. Szymusiak J, Fox MD, Polak C, et al. An inpatient patient safety curriculum for pediatric residents. *MedEdPORTAL*. 2018;14:10705. https://doi.org/10.15766/mep_2374-8265.10705
16. Diemer G, Jaffe R, Papanagnou D, Zhang XC, Zavodnick J. Patient safety escape room: a graduate medical education simulation for event reporting. *MedEdPORTAL*. 2019;15:10868. https://doi.org/10.15766/mep_2374-8265.10868
17. Mirza A, Winer J, Garber M, Makker K, Maraqa N, Alissa R. Primer in patient safety concepts: simulation case-based training for pediatric residents and fellows. *MedEdPORTAL*. 2018;14:10711. https://doi.org/10.15766/mep_2374-8265.10711
18. Halbach J, Sullivan L. Medical errors and patient safety: a curriculum guide for teaching medical students and family practice residents. *MedEdPORTAL*. 2005;1:101. https://doi.org/10.15766/mep_2374-8265.101
19. Gehlbach H, Artino AR Jr. The survey checklist (manifesto). *Acad Med*. 2018;93(3):360-366. https://doi.org/10.1097/ACM.0000000000002083
20. Lawton R, Parker D. Barriers to incident reporting in a healthcare system. *Qual Saf Health Care*. 2002;11(1):15-18. https://doi.org/10.1136/qhc.11.1.15
21. Kirkpatrick JD, Kirkpatrick WK. *Kirkpatrick's Four Levels of Training Evaluation*. ATD Press; 2016.
22. McHugh ML. Interrater reliability: the kappa statistic. *Biochem Med (Zagreb)*. 2012;22(3):276-282. https://doi.org/10.11613/BM.2012.031

Received: March 2, 2021
Accepted: July 26, 2021
Published: October 11, 2021

Copyright © 2021 Arnell et al. This is an open-access publication distributed under the terms of the Creative Commons Attribution license.
